# Tumor-activated lymph node fibroblasts suppress T cell function in diffuse large B cell lymphoma

**DOI:** 10.1172/JCI166070

**Published:** 2023-07-03

**Authors:** Benedetta Apollonio, Filomena Spada, Nedyalko Petrov, Domenico Cozzetto, Despoina Papazoglou, Peter Jarvis, Shichina Kannambath, Manuela Terranova-Barberio, Rose-Marie Amini, Gunilla Enblad, Charlotte Graham, Reuben Benjamin, Elisabeth Phillips, Richard Ellis, Rosamond Nuamah, Mansoor Saqi, Dinis P. Calado, Richard Rosenquist, Lesley A. Sutton, Jon Salisbury, Georgios Zacharioudakis, Anna Vardi, Patrick R. Hagner, Anita K. Gandhi, Marina Bacac, Christina Claus, Pablo Umana, Ruth F. Jarrett, Christian Klein, Alexander Deutsch, Alan G. Ramsay

**Affiliations:** 1 School of Cancer and Pharmaceutical Sciences, Faculty of Life Sciences & Medicine, King’s College London, London, United Kingdom.; 2 BRC Advanced Cytometry Platform and; 3 BRC Translational Bioinformatics at Guy’s and St. Thomas’s NHS Foundation Trust and King’s College London, London, United Kingdom.; 4 Division of Digestive Diseases, Faculty of Medicine, Imperial College London, London, United Kingdom.; 5 BRC Genomics Research Platform at Guy’s and St. Thomas’s NHS Foundation Trust and King’s College London, London, United Kingdom.; 6 Department of Immunology, Genetics and Pathology, Uppsala University and Hospital, Uppsala, Sweden.; 7 Immunity & Cancer Laboratory, Francis Crick Institute, London, United Kingdom.; 8 Department of Molecular Medicine and Surgery, Karolinska Institutet, Stockholm, Sweden.; 9 Department of Haematology, King’s College Hospital NHS Foundation Trust, London, United Kingdom.; 10 5th Surgical Department, Aristotle University of Thessaloniki, Thessaloniki, Greece.; 11 Hematology Department and HCT Unit, G. Papanikolaou Hospital, Thessaloniki, Greece.; 12 Bristol-Myers Squibb, Summit, New Jersey, USA.; 13 Roche Innovation Center Zurich, Schlieren, Switzerland.; 14 MRC–University of Glasgow Centre for Virus Research, Glasgow, United Kingdom; 15 Division of Hematology, Medical University of Graz, Graz, Austria.

**Keywords:** Immunology, Oncology, Cancer immunotherapy, Lymphomas

## Abstract

Recent transcriptomic-based analysis of diffuse large B cell lymphoma (DLBCL) has highlighted the clinical relevance of LN fibroblast and tumor-infiltrating lymphocyte (TIL) signatures within the tumor microenvironment (TME). However, the immunomodulatory role of fibroblasts in lymphoma remains unclear. Here, by studying human and mouse DLBCL-LNs, we identified the presence of an aberrantly remodeled fibroblastic reticular cell (FRC) network expressing elevated fibroblast-activated protein (FAP). RNA-Seq analyses revealed that exposure to DLBCL reprogrammed key immunoregulatory pathways in FRCs, including a switch from homeostatic to inflammatory chemokine expression and elevated antigen-presentation molecules. Functional assays showed that DLBCL-activated FRCs (DLBCL-FRCs) hindered optimal TIL and chimeric antigen receptor (CAR) T cell migration. Moreover, DLBCL-FRCs inhibited CD8^+^ TIL cytotoxicity in an antigen-specific manner. Notably, the interrogation of patient LNs with imaging mass cytometry identified distinct environments differing in their CD8^+^ TIL-FRC composition and spatial organization that associated with survival outcomes. We further demonstrated the potential to target inhibitory FRCs to rejuvenate interacting TILs. Cotreating organotypic cultures with FAP-targeted immunostimulatory drugs and a bispecific antibody (glofitamab) augmented antilymphoma TIL cytotoxicity. Our study reveals an immunosuppressive role of FRCs in DLBCL, with implications for immune evasion, disease pathogenesis, and optimizing immunotherapy for patients.

## Graphical abstract

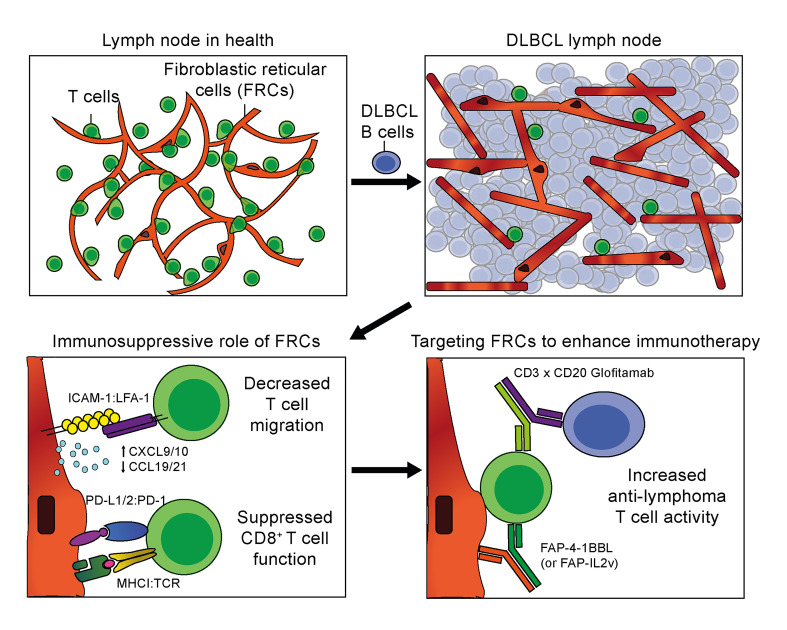

## Introduction

Diffuse large B cell lymphoma (DLBCL) is an aggressive tumor of mature B cells that arises in LNs (1). DLBCL remains incurable for approximately 40% of patients who experience treatment failure after standard R-CHOP (cyclophosphamide, doxorubicin, prednisone, rituximab, and vincristine) immunochemotherapy. In-depth molecular studies of malignant B cells have revealed extensive heterogeneity and increased the understanding of tumor cell–intrinsic pathogenesis to aid the development of personalized targeted therapy (2). DLBCL is classified into 2 major molecular subtypes relating to the developmental cell of origin (COO) of tumor B cells: the germinal center (GC) B cell–like (GCB) and the activated B–cell like (ABC) subtypes, with the latter having inferior outcomes (3–5). More recently, genetic subtypes with distinct outcomes have been identified within these COO subgroups (6–10).

DLBCL tumors are heavily packed with tumor B cells that efface normal tissue and mask the presence of nonmalignant cell types within the tumor microenvironment (TME) (11). However, the TME is a complex ecosystem, comprising not only malignant cells, but also immune and stromal cells. A seminal gene expression study of diagnostic whole-tissue biopsies alluded to the importance of the concealed TME: signatures derived from stromal cells significantly associated with patient outcomes, including the prognostically favorable stromal-1 signature containing myofibroblast- and extracellular matrix–associated (ECM-associated) genes (12). More recently, advanced transcriptome studies of bulk DLBCL tissues have again illuminated the relevance of the stromal and immune cell landscape in lymphoma, with the categorization of TME ecosystems that capture clinical heterogeneity and extend beyond COO and genotypic classifications (13, 14).

Stroma-immune crosstalk is highly relevant in the immunotherapy era. Immunotherapy for lymphoma has shown promise, as illustrated by programmed cell death protein 1 (PD-1) blockade in Hodgkin lymphoma. “Hot” tumors, which harbor relatively high numbers of tumor-infiltrating lymphocytes (TILs), are associated with superior response to checkpoint inhibitors and include a subset of DLBCL tumors (15). However, the majority of non-Hodgkin lymphomas (NHLs), including DLBCL, fall between currently ill-defined “immunosuppressed” and “cold” TME categories (16, 17), which likely contributes to suboptimal responses to anti–PD-1 or chimeric antigen receptor (CAR) T cell immunotherapies (18–20). Although fibroblasts were initially viewed as “immune neutral” structural determinants, studies have revealed that they play a critical role in regulating immune cells and influencing response to immunotherapy (21).

The hallmark feature of lymphoid organs is the highly ordered compartmentalization of T cells, B cells, and myeloid cells into specialized niches to generate effective immune responses. Immune-interacting specialized fibroblasts known as fibroblastic reticular cells (FRCs) underpin these distinct microenvironmental niches to provide structural integrity and crucially regulate innate and adaptive immune responses during homeostasis and immune activation (22, 23). During inflammation, FRCs remodel to accommodate reactive LN (rLN) expansion, before normalizing upon immune resolution. FRCs dynamically steer efficient immune responses by secretion of supporting factors, cytokines and chemokines, expression of activating molecules, and antigen presentation — to control immune crosstalk and activation states (24, 25). Importantly, FRCs play a dual role in enhancing T cell activation while also restraining excessive T cell inflammation via the expression or release of inhibitory molecules to prevent immunopathology (26–28). Despite the transcriptome-based descriptions of DLBCL ecosystems, the immunomodulatory role of fibroblasts in lymphoma is poorly defined.

Here, we studied primary samples from human patients and a murine model to unmask the phenotypical, transcriptional, and functional consequences for FRCs chronically exposed to DLBCL. In response to inflammatory signals from malignant B cells, FRCs acquired an activated phenotype and increased their expression of the cancer-associated fibroblast (CAF) marker fibroblast-activated protein (FAP). DLBCL transcriptionally reprogramed FRCs, altering immunoregulatory chemokine and antigen-presentation pathways. Functional assays demonstrate that tumor-altered expression of chemokines and adhesion molecules in FRCs leads to a reduced ability to promote TIL and CAR T cell migration. In addition, DLBCL-activated FRCs (DLBCL-FRCs) inhibited CD8^+^ TIL cytolytic activity via aberrant expression of PD-1 ligands (PD-L1 and PD-L2). Moreover, the interrogation of patient LNs identified distinct environments differing in their CD8^+^ TIL-FRC composition and spatial organization that associated with survival outcomes. Finally, tissue organotypic cultures provided proof of principle that FRCs can be targeted with FAP-targeted immunostimulatory fusion proteins to augment antitumor TIL cytotoxicity elicited by T cell-engaging bispecific antibody (TCB) immunotherapy.

## Results

### FRCs are expanded and remodeled in DLBCL.

**Figure 1:**
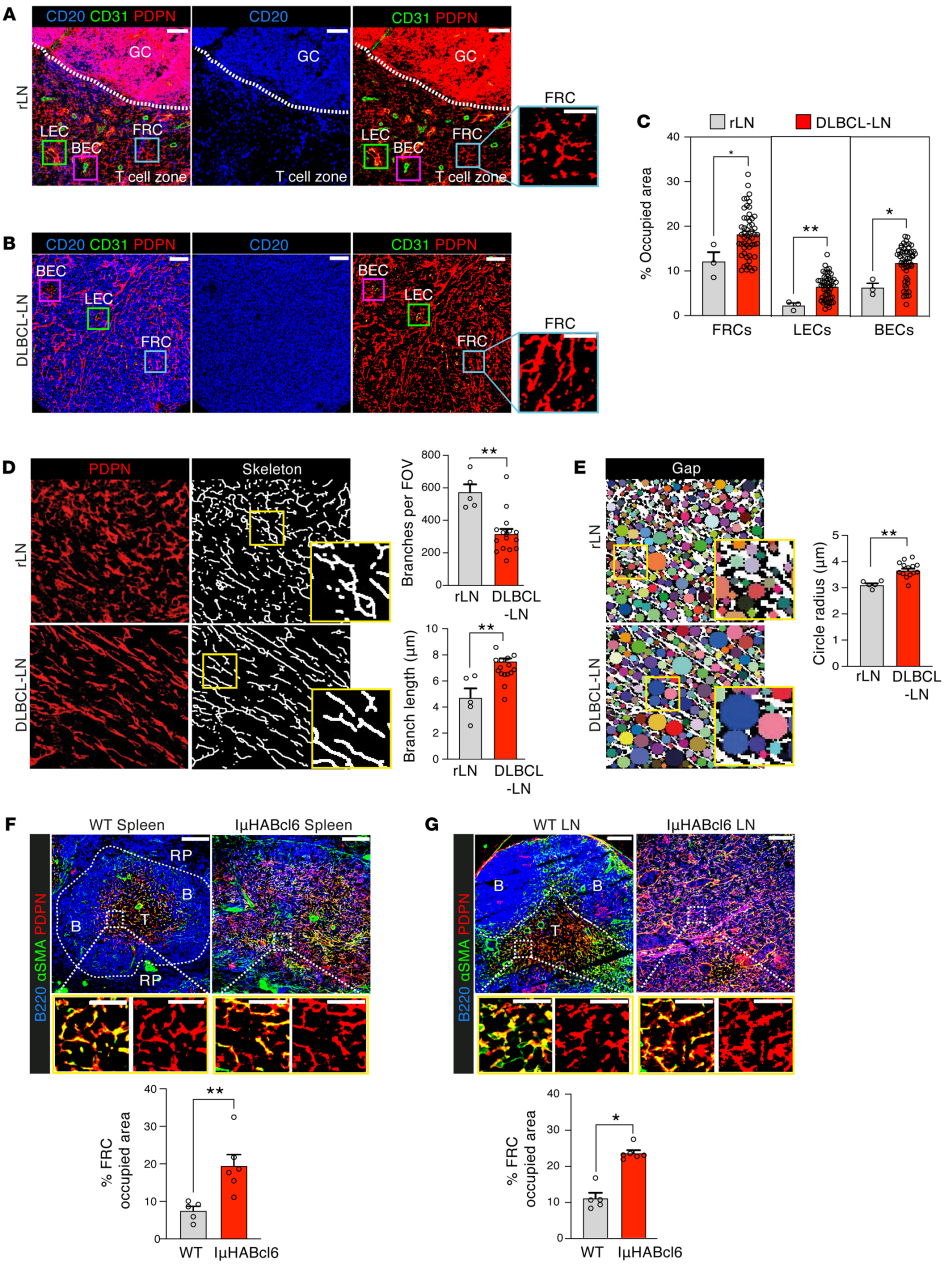
Aberrantly remodeled FRCs in human and murine DLBCL. (**A** and **B**) Representative IMC staining of B cells (CD20) and the LN stromal population FRCs (higher magnification insets), BECs, and LECs (CD31, PDPN) in (**A**) rLN (*n* = 3) and (**B**) DLBCL-LN TMA (*n* = 53). Scale bars: 100 μm. (**C**) Area occupied by FRCs, LECs, and BECs in rLN (*n* = 3) and DLBCL-LN tissues (*n* = 53) (IMC). Two distinct biopsy cores per patient sample (data points). (**D**) PDPN^+^ FRCs in rLN (*n* = 5) and DLBCL-LNs (*n* = 15) examined using skeleton analysis. Left, original PDPN signal; right, skeletonized images. Quantification of the mean number of branches and lengths per field of view. (**E**) Binary images of PDPN staining for gap analysis (colored circles) of the FRC network in rLN (*n* = 5) and DLBCL-LNs (*n* = 15). Gap (circle) radii analysis. Original magnification, ×20. (**F** and **G**) Representative confocal analysis of the FRC network in the spleens (**F**) and LNs (**G**) of WT and IμHABcl6 lymphoma mice. Scale bars: 100 μm (upper panels); 50 μm (lower panels). B, B cell zone; T, T cell zone; RP, red pulp. Area occupied analysis of PDPN^+^ FRCs in spleen (**F**) and LN tissue images (**G**) from WT (*n* = 5) and lymphoma IμHA*Bcl6* (*n* = 6) mice. Data are represented as mean ± SEM (**C**–**G**). *P < 0.05; **P < 0.01, Mann-Whitney *U* test (**C**–**G**).

To reveal the stromal landscape within DLBCL TMEs, we performed high-dimensional imaging mass cytometry (IMC) analysis of a tissue microarray (TMA) containing 53 patient LNs (2 core areas per biopsy) (Supplemental Table 1; supplemental material available online with this article; https://doi.org/10.1172/JCI166070DS1) (29), with nonmalignant rLNs acting as controls. The application of a stroma-identification pipeline (Supplemental Figure 1A and Supplemental Table 2) delineated the 3 major LN populations defined by the expression of podoplanin (PDPN) and endothelial cell marker CD31 (30, 31): FRCs (PDPN^+^CD31^–^), lymphatic endothelial cells (LECs: PDPN^+^CD31^+^), and blood endothelial cells (BECs: PDPN^–^CD31^+^). Interestingly, we observed a significant increase of these stromal populations in DLBCL compared with rLNs, with FRCs occupying the largest LN area, irrespective of the DLBCL COO (Figure 1, A–C, and Supplemental Figure 1B). IMC images revealed the highly ordered compartmentalization of B cells within GCs, adjacent to the T cell zone of rLNs containing an intricate and interconnected FRC network, a hallmark feature of secondary lymphoid organs (Figure 1A). In stark contrast, DLBCL biopsies showed loss of B/T cell zone compartmentalization and an LN structure that was effaced by CD20^+^ tumor B cells (Figure 1B).

Multicolor confocal imaging analysis confirmed the prominent expansion of a remodeled PDPN^+^ (CD31^–^) FRC network that coexpressed the myofibroblast marker SMA^+^ in an independent cohort of whole DLBCL-LNs in comparison with rLNs (Supplemental Figure 1, C–E). FRCs were diffusely distributed in DLBCL-LNs and appeared less organized and denser, while exhibiting a distinctly stretched morphology compared with rLNs. To exclude the possibility that increased PDPN^+^ CD31^–^ cells in DLBCL included follicular DCs (FDCs), we measured the expression of CD21/35 to distinguish this normally GC localized subset. We did not detect expression of this receptor on the expanded PDPN^+^ FRC network in DLBCL, except for residual GCs, detected in 3 of the 53 DLBCL-LNs examined (Supplemental Figure 1F). In addition, the coexpression of collagen I and desmin with PDPN demonstrated that myofibroblasts in DLBCL were of FRC origin (Supplemental Figure 1G). To investigate the magnitude of FRC network remodeling in DLBCL, we performed skeleton analysis, which revealed fewer branch points and increased branch lengths compared with rLNs (Figure 1D). Morphological classification of our IMC data set confirmed increased numbers of elongated and less complex reticular fibers in DLBCL (Supplemental Figure 1, H–L). In line with these observations, a gap-analysis algorithm showed larger spaces between reticular network branches in lymphoma LNs, consistent with a loosened FRC network (Figure 1E). This remodeled network is reminiscent of FRC activation and stretching triggered during an immune response (31, 32). However, our data reveal an aberrantly remodeled FRC state in DLBCL beyond what is detected in rLNs.

An expansion of stretched PDPN^+^, SMA^+^ FRCs was similarly detected in diseased spleens and LNs from IμHA*Bcl6* mice (Figure 1, F and G), a mouse model of spontaneous DLBCL in which *Bcl6* expression is targeted to mature B cells under the IgH I promoter (IμHA*Bcl6*), mimicking a common genetic lesion in DLBCL (33). Although the model has low penetrance and a long disease latency (from 10 months of age), it was chosen as it recapitulates the salient clinical and histopathological features and genetics of the human disease (Supplemental Figure 2, A–D). Confocal microscopy revealed effacement of tissue microarchitecture by DLBCL cells, with loss of B/T cell zone compartmentalization in expanded splenic white pulp and LNs from IμHA*Bcl6* mice compared with age-matched WT mice. The coexpression of the FRC-associated antigen ER-TR7 with PDPN confirmed the identity of expanded and remodeled FRCs in this mouse model (Supplemental Figure 2, E–H). Together, these data reveal an expansion of remodeled FRCs in the human DLBCL TME that is recapitulated in the IμHA*Bcl6* model.

### DLBCL B cell–activated FRCs upregulate expression of the CAF marker FAP.

**Figure 2:**
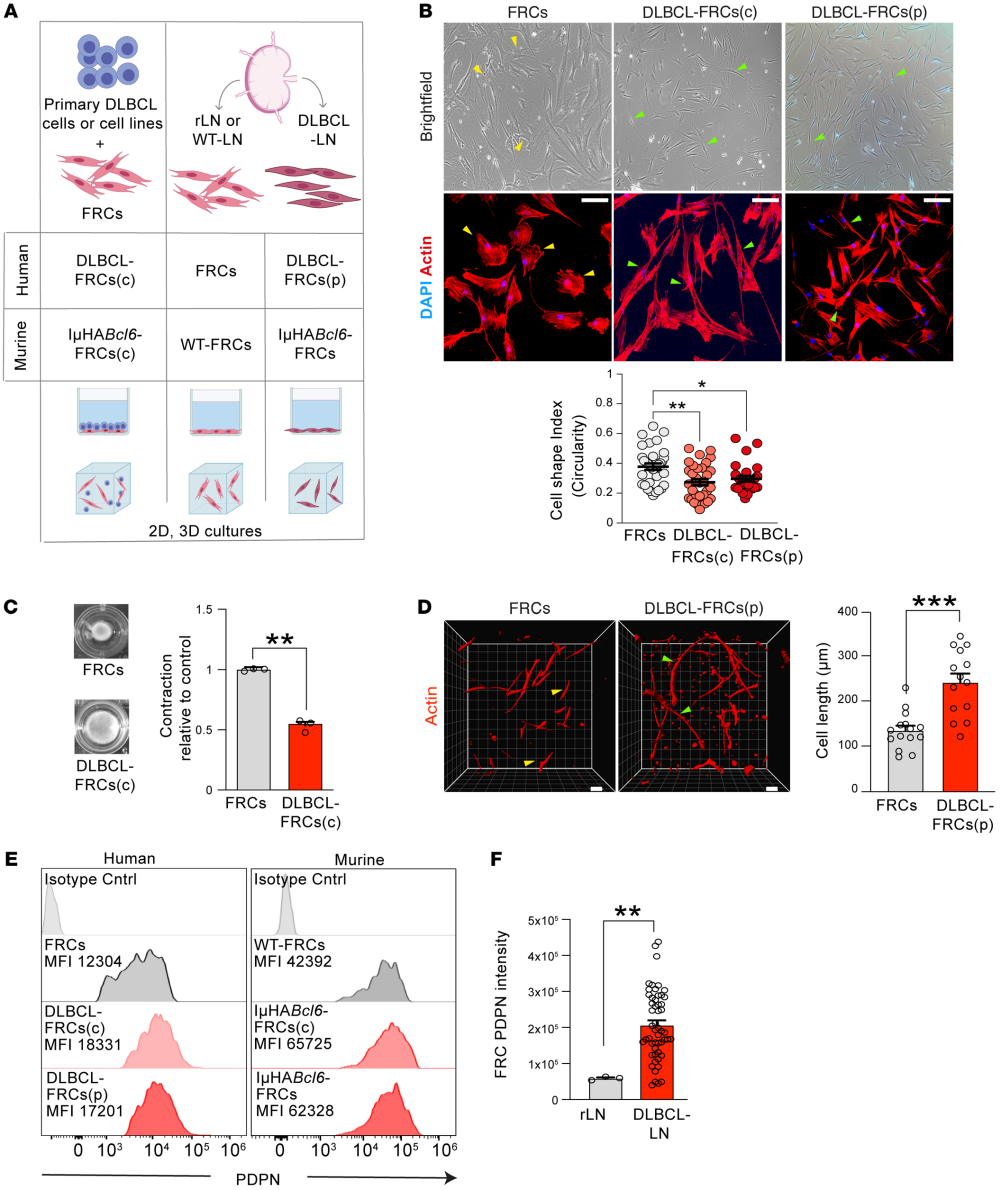
Coculture models recapitulate remodeled DLBCL-FRCs. (**A**) Schematic of 2D and 3D DLBCL-FRC crosstalk cultures. Primary FRCs were conditioned with DLBCL cell lines (5 days) or with primary DLBCL B cells (3 days) (human, DLBCL-FRCs[c]; murine, IμHA*Bcl6*-FRCs[c]). Primary FRCs were isolated from rLNs (human, FRCs; murine, WT-FRCs) or from DLBCL-LN patient biopsies (human, DLBCL-FRCs[p]); murine IμHA*Bcl6*-FRCs). (**B**) Representative brightfield (top), confocal images (bottom), and analysis (dot plot) of FRCs (*n* = 6), DLBCL-FRCs(c) (conditioned with primary DLBCL cells, *n* = 6 patients), and DLBCL-FRCs(p) (*n* = 2 patients) (ABC and GCB). Scale bars: 10 μm. (**C**) 3D contraction assays for FRCs and DLBCL-FRCs(c) (SU-DHL16). Brightfield gel images at 3 days. (**D**) 3D images and length analysis of FRCs (*n* = 3) and DLBCL-FRCs(p) (*n* = 2 patients, ABC and GCB). Scale bars: 10 μm. (**E**) PDPN expression histograms. Left, FRCs (gray, *n* = 3), DLBCL-FRCs(c) (primary DLBCL cells, light red, *n* = 3 patients) and DLBCL-FRCs(p) (dark red, *n* = 3 patients, 1 ABC and 2 GCB). Right, WT-FRCs (gray, *n* = 3), IμHA*Bcl6*-FRCs(c) (light red, *n* = 3), and IμHA*Bcl6*-FRCs (dark red, *n* = 3). (**F**) IMC quantification of PDPN expression on FRCs from rLN (*n* = 3) and DLBCL-LNs (*n* = 53). (**D**) Representative data from *n* = 3 independent experiments. Data are represented as mean ± SEM (**B**, **C**, **D**, and **F**). *P < 0.05; **P < 0.01; ***P < 0.001, 1-way ANOVA with Tukey’s test (**B**) or Mann-Whitney *U* test (**C**, **D**, and **F**).

FRCs are contractile during homeostasis, but rapidly respond to lymphocyte-derived proinflammatory stimuli and lose their contractility to facilitate LN swelling during an immune response (32). To investigate how FRCs respond to DLBCL, we established human and murine 2D and 3D culture platforms utilizing primary LN-FRCs (Figure 2A and Supplemental Figure 3, A–C) (30). Briefly, we refer to FRCs following direct contact coculture with primary DLBCL B cells (3 days) or cell lines (5 days) as DLBCL-conditioned FRCs (human: DLBCL-FRCs[c]; murine: IμHA*Bcl6*-FRCs[c]). We also studied early passage FRCs directly expanded from patient DLBCL-LNs (human: DLBCL-FRCs[p]; murine: IμHA*Bcl6*-FRCs). We found that DLBCL-FRCs(c) (irrespective of COO) and IμHA*Bcl6*-FRCs(c) lost their contractile shape and showed marked stretching compared with unconditioned FRCs (FRCs) (Figure 2B and Supplemental Figure 3, D–F). We further noted that primary DLBCL B cells induced greater FRC stretching compared with control rLN-derived primary B cells (Supplemental Figure 3G). Importantly, DLBCL-FRCs(p) and IμHA*Bcl6*-FRCs exhibited similar elongated morphology. To determine functional relevance, we utilized 3D collagen-based gels that allow FRCs to remodel the matrix and more closely simulate the LN microenvironment (34). We found that DLBCL-FRCs(c) also exhibited similar stretching in 3D culture (Supplemental Figure 3H) and a decreased ability to contract gel matrices compared with FRCs (Figure 2C). Importantly, these findings were mirrored when we assayed DLBCL-FRCs(p) (Figure 2D).

We next investigated the expression of PDPN that promotes contractile signaling in FRCs (32). We found that DLBCL-FRCs(c) and IμHA*Bcl6*-FRCs(c) upregulated surface expression of PDPN to levels comparable to those of DLBCL-FRCs(p) and IμHA*Bcl6*-FRCs, respectively (Figure 2E). We validated these findings and identified upregulation of *Pdpn* mRNA in DLBCL-FRCs(c) (Supplemental Figure 3I), while IMC analysis showed increased PDPN expression on FRCs in DLBCL biopsies (Figure 2F). These data prompted us to explore why DLBCL-FRCs(c) were noncontractile. We found that interaction with DLBCL cells induced the accumulation of PDPN into lipid rafts where it colocalized with its inhibitory partner CD44 (Supplemental Figure 3, J and K), reduced actin-dependent contractile signaling (Supplemental Figure 3L), and triggered a switch in Rho A–to–Rac-1 activation (Supplemental Figure 3, M and N), in keeping with FRC stretching (32). Collectively, these data support the utility of coculture systems to recapitulate the in situ findings of a remodeled PDPN^+^ FRC network within DLBCL TMEs.

**Figure 3:**
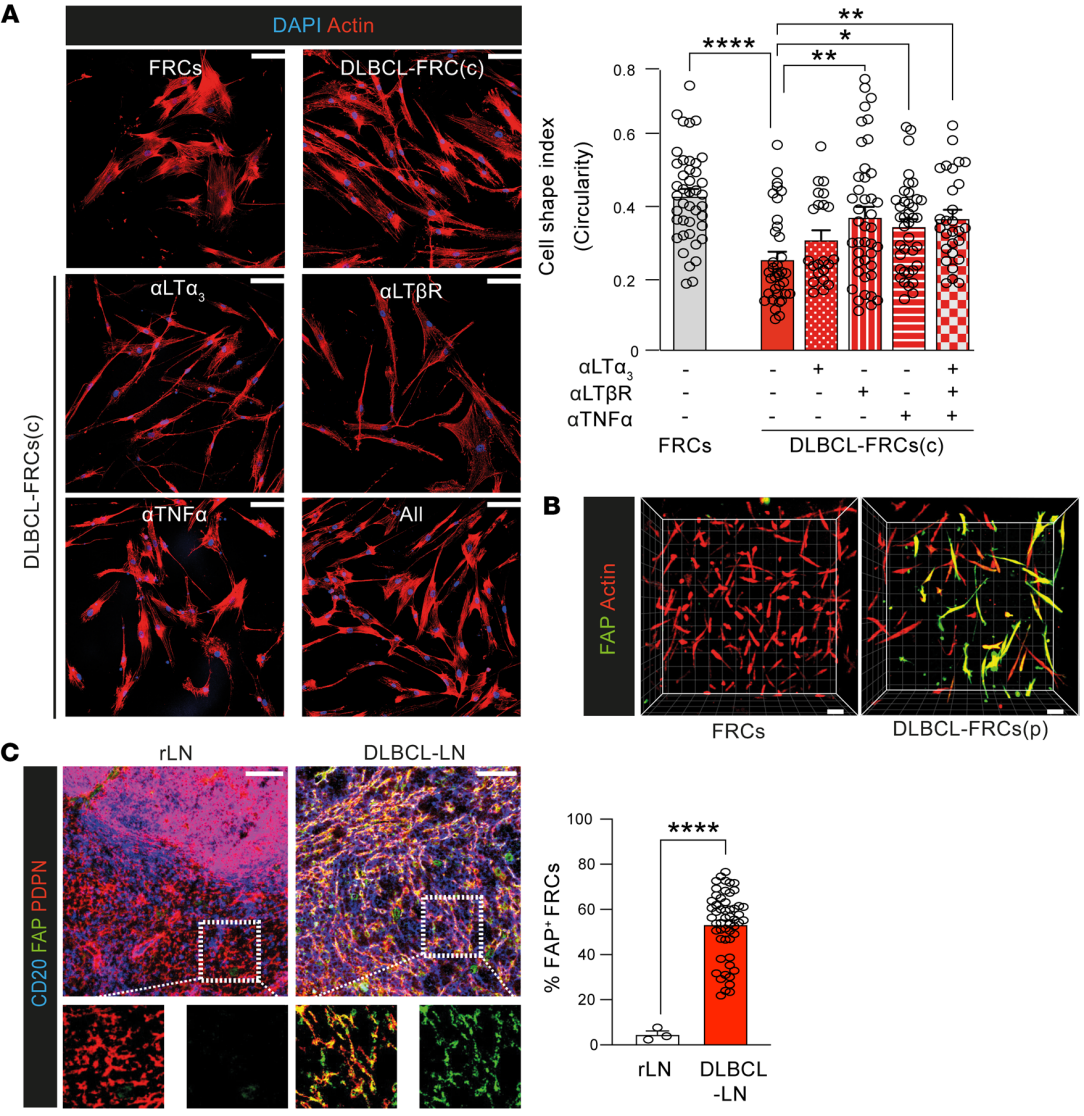
DLBCL-FRCs express elevated CAF marker FAP. (**A**) Cell-shape analysis of FRCs and DLBCL-FRCs(c) (SU-DHL16) treated with isotype or blocking/neutralizing antibodies. Scale bars: 10 μm, Right, quantification. (**B**) 3D images of FRC and DLBCL-FRC(p) gels stained as indicated. Scale bars: 10 μm. (**C**) IMC images of rLN (*n* = 3, same representative tissue presented in Figure 1A) and DLBCL-LNs (*n* = 53) stained for CD20 (B cells), PDPN (FRCs), and FAP. Scale bars: 100 μm. Frequency of FAP^+^ FRCs quantified using IMC. (**A**) Representative data from *n* = 3 independent experiments. Data are represented as mean ± SEM (**A** and **C**). *P < 0.05; **P < 0.01; ****P <.0001, 1-way ANOVA with Tukey’s test (**A**) or Mann-Whitney *U* test (**C**).

To investigate which DLBCL molecules contributed to FRC activation, we first compared direct contact coculture with a Transwell system (Supplemental Figure 4A). Although loss of FRC contractility was maximally induced following cell contact, Transwell cocultures also allowed significant FRC remodeling (Supplemental Figure 4B), indicating the involvement of both soluble and membrane-bound tumor factors. Since B cell–derived LT_12_, LT_3_, and TNF are known to activate FRCs (35, 36), we chose to examine expression of these cytokines and their receptors in DLBCL. We detected robust LT_12_, LT_3_, and TNF expression in primary DLBCL cells during coculture and in situ (Supplemental Figure 4C). Additionally, we detected LTR as well as TNFRI and TNFRII on human and murine FRCs (Supplemental Figure 4, D and E). To assess whether DLBCL-expressed LTs and TNF contributed to FRC remodeling, we tested the addition of blocking antibodies to cocultures. Our screening revealed that blockade of LTR or neutralization of TNF alone was sufficient to significantly prevent DLBCL-induced FRC elongation, while neutralizing LT3 showed partial attenuation (Figure 3A). Notably, we did not detect any additive effect of blocking all 3 molecules in this assay. Consistent with these findings, the addition of an LTR-activating antibody or recombinant LT_3_ or TNF to cultures induced FRC stretching and augmented PDPN expression (Supplemental Figure 4, F and G). Thus, these results suggest that membrane LT_12_ as well as soluble LT_3_ and TNF produced by DLBCL B cells contribute to FRC activation.

In keeping with a tumor-activated phenotype, we also detected increased expression of the CAF marker FAP (37, 38) by reverse-transcriptase PCR (RT-PCR) and immunofluorescence analysis of DLBCL-FRCs(c) (p) (Supplemental Figure 4, H and I, and Figure 3B). IMC (Figure 3C) and confocal analysis (Supplemental Figure 4J) of DLBCL-LNs revealed that FRCs expressed strikingly higher levels of FAP compared with rLNs. We similarly detected augmented expression of FAP on remodeled FRCs in DLBCL-LNs and spleens from IμHA*Bcl6* mice (Supplemental Figure 4K). Since FRCs support normal and follicular lymphoma B cell survival (36, 39), we explored whether they could also promote DLBCL survival. Coculturing serum-deprived DLBCL cells with FRCs in 2D and 3D cultures enhanced their survival compared with culturing tumor cells alone (Supplemental Figure 4, L and N). We next investigated whether FRCs in DLBCL-LNs expressed the B cell survival cytokine BAFF (39). Confocal analysis revealed an increased frequency of FRCs expressing BAFF in DLBCL compared with rLN interfollicular FRCs (Supplemental Figure 4M). Importantly, the addition of a BAFF-neutralizing antibody to DLBCL-FRC 3D cultures abrogated the tumor-protective effect of FRCs (Supplemental Figure 4N). Thus, these data identify FAP as a marker for DLBCL-FRCs that exhibit the hallmark CAF function of supporting tumor cell survival (38).

### Lymphoma transcriptionally reprograms FRCs altering immunoregulatory pathways.

**Figure 4:**
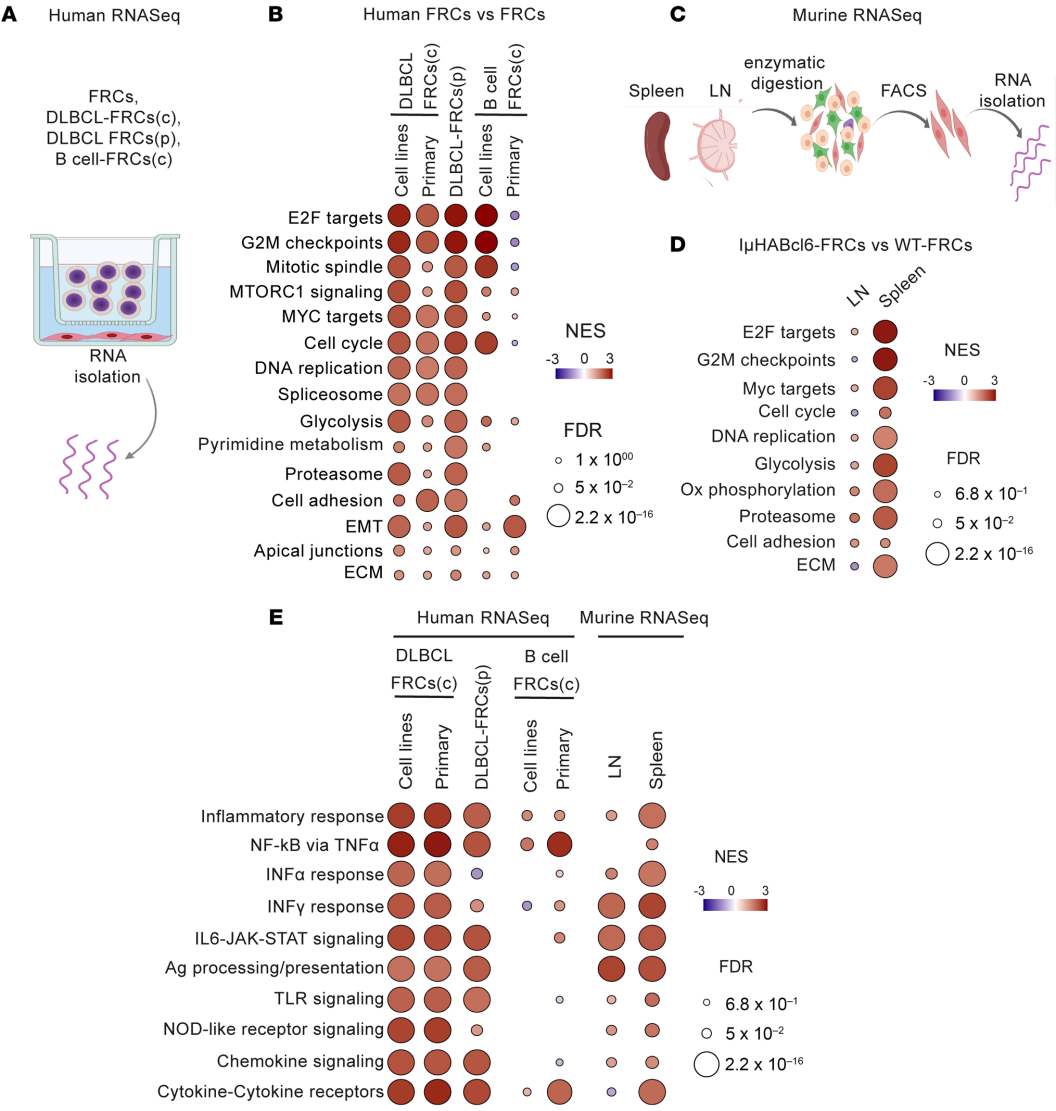
DLBCL B cells reprogram FRCs into an activated state. (**A**) Experimental strategy for human bulk RNA-Seq. RNA was extracted from FRCs (*n* = 3), DLBCL-FRCs(c) cocultured with DLBCL cell lines (*n* = 8), or primary DLBCL B cells (*n* = 4 patients) and B cell–FRCs(c) cocultured with B cell lines (*n* = 3) or primary rLN-derived B cells (*n* = 3) for 48 hours using Transwell. In parallel, RNA was extracted from DLBCL-FRCs(p) (*n* = 2 patients). (**B**) GSEA fibroblast activation pathways in human DLBCL-FRCs(c) or DLBCL-FRCs(p) versus FRCs. (**C**) Murine low-input bulk RNA-Seq workflow. Spleens and LNs from IμHA*Bcl6* (*n* = 3, *n* = 7 respectively) and WT mice (*n* = 6, *n* = 5 respectively) were processed for FRC isolation (FACS). (**D**) GSEA fibroblast activation pathways in IμHA*Bcl6*-FRCs versus WT-FRCs from spleen and LNs. (**E**) GSEA immunologically relevant pathways in human and murine bulk gene expression profiles. Circle colors depict the normalized enrichment score (NES) and FDR.

The above data supporting lymphoma-induced activation of FRCs led us to define how DLBCL alters the transcriptome of FRCs. We first generated transcriptional profiles of DLBCL-FRCs(c) following conditioning with DLBCL cell lines (*n* = 8) or primary DLBCL cells (*n* = 4) (Figure 4A and Supplemental Table 3) and found differentially expressed genes (DEGs) compared with FRCs (Supplemental Figure 5, A and B). Gene set enrichment analysis (GSEA) and DEG analysis identified upregulation of genes involved in proliferation (*MKi67*, *CDK1*, *BUB1B*, *CDC20*), metabolism (*GYS2*, *STC1*, *KIF20*), adhesion (*ITGA8*, *ICAM1*, *VCAN*), and ECMECM(*MMP9*, *MMP10*, *COL4A4*) pathways in DLBCL-FRCs (Figure 4B and Supplemental Figure 5C). Notably, analogous transcriptional alterations were also observed in DLBCL-FRCs(p) (*n* = 2, Figure 4B and Supplemental Figure 5, D and E), providing evidence that chronic exposure to lymphoma within the TME keeps FRCs in an activated state. We further noted that conditioning with DLBCL cells induced a more metabolically active FRC transcriptome compared with nonmalignant B cell controls (B cell–FRCs[c]). Additionally, low-input RNA-Seq analysis of PDPN^+^CD31^–^ FRCs sorted from spleens or LNs of IμHA*Bcl6* and WT mice (Figure 4C and Supplemental Figure 5, H–K) identified similarly reprogrammed pathways in IμHA*Bcl6*-FRCs, most notably from splenic TMEs that harbored most disease (Figure 4D and Supplemental Figure 5L). Moreover, immunomodulatory pathways were significantly enriched in both human and murine DLBCL-FRCs, including increased gene expression in inflammatory IFN type I and II responses (*Irf8*, *Ifi27*), cytokine and chemokine (*Cxcl9*, *Cxcl10*), and MHC class I and II antigen-presentation (HLA genes, *B2m* and *Cd74*) pathways (Figure 4E and Supplemental Figure 5, F, G, and M). We found that DLBCL-FRCs showed greater enhancement of these altered immune regulatory pathways compared with FRCs conditioned with nonmalignant B cells. In contrast, we observed downregulated homeostatic chemokine (*Ccl21* and *Ccl19*) expression in IμHA*Bcl6*-FRCs. Together, these analyses reveal that acute or chronic exposure to DLBCL induces an altered immunoregulatory state in FRCs, with similarity in how they respond to inflammatory stimuli (24, 40, 41).

**Figure 5:**
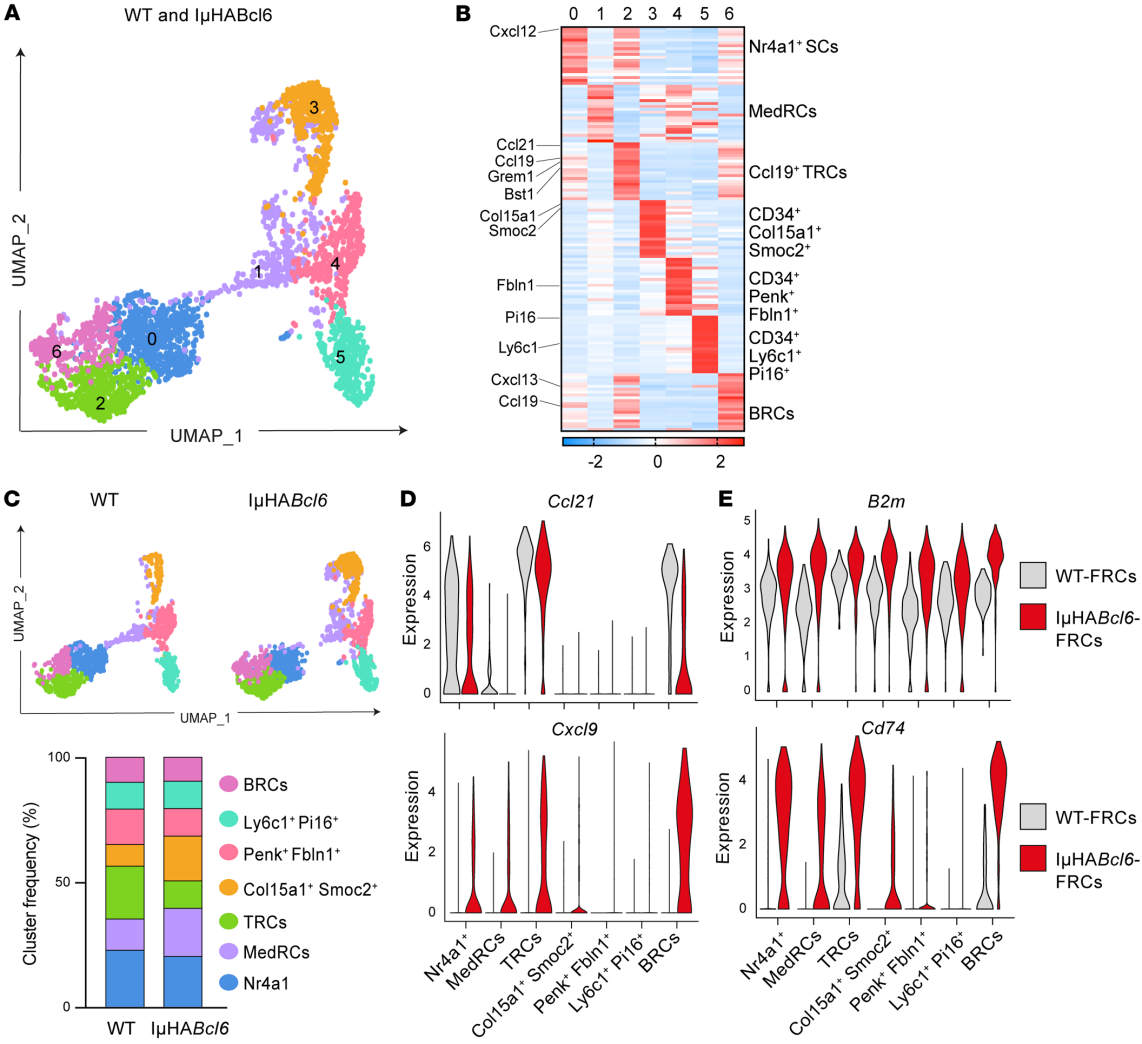
scRNA-Seq of murine DLBCL-FRCs reveals altered chemokine and antigen-presentation gene pathways. (**A**) UMAP of scRNA-Seq data generated from FACS-sorted LN stromal cells for WT-FRCs (1,408 cells) and IμHA*Bcl6*-FRCs (1,422 cells). Seven clusters (c0–c6) identified with FRC-reclustered analysis. (**B**) Heatmap showing the top 20 genes and average expression levels in each cluster and their assigned identity (FDR < 0.001 and highest log-fold changes). (**C**) Distribution of FRC clusters in IμHA*Bcl6* versus WT. Upper panels, UMAP of FRC clusters across the WT (left) and IμHA*Bcl6* (right) samples. Lower panel, histogram showing frequency of FRC clusters in WT and IμHA*Bcl6*. (**D** and **E**) Violin plots of *Ccl21* and *Cxcl9* (**D**)and *B2m* and *Cd74* (**E**) expression in IμHA*Bcl6*-FRC versus WT-FRC clusters.

To gain enhanced resolution of how lymphoma reprograms FRCs, we also performed single-cell RNA-Seq (scRNA-Seq) on sorted LN stromal cells (CD45^–^) from IμHA*Bcl6* mice (4,686 cells) and age-matched WT mice (2,779 cells). Unsupervised clustering of the combined samples allowed the identification of the major LEC, BEC, and FRC stromal populations visualized with uniform manifold approximation and projection (UMAP) (Supplemental Figure 5, N–P) (42). FRC-reclustered analysis (*n* = 2,975 cells) revealed 7 clusters that we assigned cross-study labels by comparing our data with previously defined FRC subsets (24, 42–44) (Figure 5, A and B, Supplemental Figure 5, Q and R, and Supplemental Table 4). We identified transcriptional signatures consistent with T-zone reticular cells (TRCs) (c2) (*Ccl19*, *Ccl21*, *Bst1*, *Grem1*); Nr4a1^+^ SCs (c0) showing some gene expression in common with c2, suggesting a possible activated TRC origin (43); medullary FRCs (MedRCs) (c1) that expressed the markers *Inmt*, *Nr4a1*, *Timp1*, and *Lum* previously associated with this subset (24, 42, 44); 3 populations of CD34^+^ FRCs (*Col15a1*^+^*Smoc2*^+^, c3; *Penk*^+^ *Fbln1*^+^, c4; and *Ly6c1*^+^ *Pi16*^+^, c5) (42); and B cell–interacting reticular cells (BRCs) (*Cxcl13*^+^, c6). GSEA confirmed enriched fibroblast activation, ECM, and IFN-response pathways in IμHA*Bcl6*-FRCs compared with WT-FRCs (Supplemental Figure 5S), in keeping with our bulk RNA-Seq data sets. scRNA-Seq analysis also revealed a reduction in the relative abundance of Ccl19^+^ TRCs (c2) in IμHA*Bcl6*-FRCs compared with WT-FRCs, whereas MedRCs (c1) and *Col15a1*^+^*Smoc2*^+^CD34^+^ FRCs (c3) increased (Figure 5C and Supplemental Figure 5T). In addition, we confirmed downregulated expression of *Ccl21,* whereas IFN-induced *Cxcl9*, *B2m*, and *Cd74* were upregulated across multiple FRC subsets (Figure 5, D and E). In sum, our results show that lymphoma reprograms FRCs, altering chemokine and antigen-presentation gene pathways that could influence the recruitment and activation of immune cells.

### DLBCL-FRCs exhibit a diminished capacity to support T lymphocyte migration.

**Figure 6:**
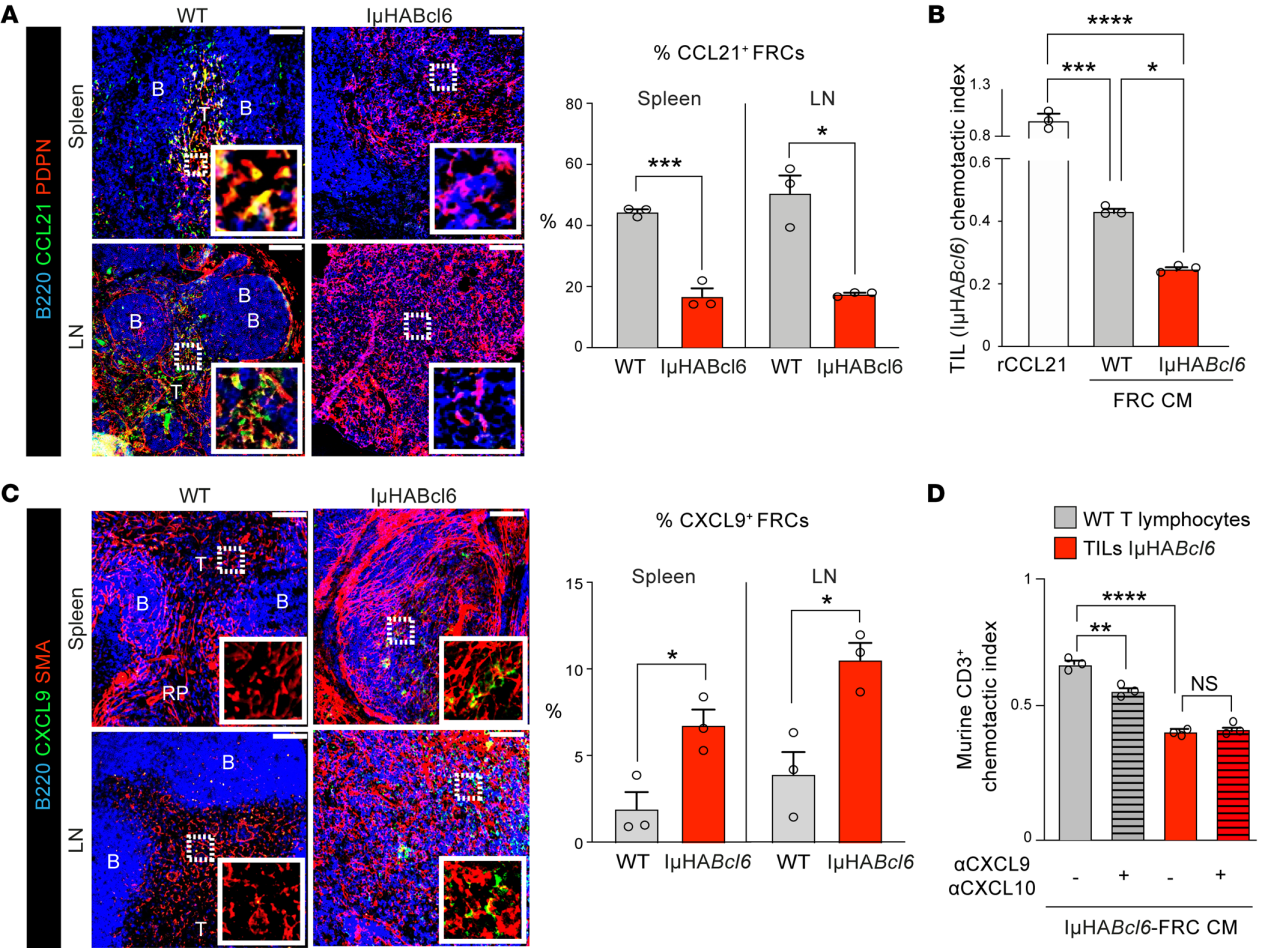
DLBCL-FRCs show a reduced ability to attract TILs. (**A**) Images of stained WT (*n* = 3) and IμHA*Bcl6* (*n* = 3) spleens and LNs. Scale bars: 100 μm. B, B cell zones; T, T cell zones. Graph shows percentages of CCL21^+^ FRCs. (**B**) TIL (IμHA*Bcl6*) chemotaxis toward recombinant CCL21, CM from WT-FRCs, or IμHA*Bcl6*-FRCs. (**C**) Images of stained WT (*n* = 3) and IμHA*Bcl6* (*n* = 3) spleens and LNs. Scale bars: 100 μm. Graph shows percentages of CXCL9^+^ FRCs. (**D**) WT T lymphocyte TIL (IμHA*Bcl6*) chemotaxis toward IμHA*Bcl6*-FRCs CM with isotype (–) or CXCL9/CXCL10–neutralizing antibodies (+). (**B** and **D**) One experiment from *n* = 5 (**B**) or *n* = 3 (**D**) independent sample experiments. Data are represented as mean ± SEM (**A**–**D**). *P < 0.05; **P < 0.01; ***P < 0.001; ****P < 0.0001, 1-way ANOVA with Tukey’s test (**B** and **D**) or Mann-Whitney *U* test (**A** and **C**).

We next asked whether lymphoma-induced reprogramming affected the ability of FRCs to recruit T cells. Confocal analysis confirmed that CCL21-expressing FRCs were significantly reduced in both murine and human DLBCL tumors (Figure 6A and Supplemental Figure 6A) and showed decreased *CCL19* expression (Supplemental Figure 6B). The diminished expression of these homeostatic chemoattractants, known to guide CCR7-expressing T cells into LNs and maintain their movement (26), led us to model the capacity of IμHA*Bcl6*-FRCs to attract TILs. Chemotaxis assays demonstrated that, although TILs migrated effectively toward CCL21 or freshly harvested conditioned media (CM) from WT-FRCs, they were incapable of substantial migration toward IμHA*Bcl6*-FRC CM (Figure 6B). As TILs expressed CCR7 (Supplemental Figure 6C), we next verified whether decreased migration was linked to reduced FRC expression of chemokine ligands. We confirmed that expanded WT-FRCs expressed CCL21 at the time CM was harvested (45), whereas IμHA*Bcl6*-FRCs expressed significantly less (Supplemental Figure 6D). Furthermore, blockade of CCR7 or neutralization of its ligands decreased the migratory potential of TILs toward CCL21 or WT-FRC CM, but had no effect on TIL migration toward DLBCL-FRC CM (Supplemental Figure 6, E and F). Intriguingly, our transcriptome analysis revealed that DLBCL-FRCs increased *Cxcl9* and *Cxcl10* expression. Confocal analysis of murine and human DLBCL biopsies confirmed increased CXCL9^+^ FRCs in situ (Figure 6C and Supplemental Figure 6G) and in cultured IμHA*Bcl6*-FRCs (Supplemental Figure 6H). As these IFN-induced chemokines have been linked to the attraction of effector T cells during acute infection (24, 46), we next asked why the CM of IμHA*Bcl6*-FRCs was ineffective at recruiting TILs. We first analyzed the expression of CXCR3, the receptor for CXCL9 and CXCL10, and found reduced levels on TILs compared with WT T lymphocytes, suggestive of desensitized CXCR3^+^ TILs (Supplemental Figure 6I). Indeed, unlike TILs, WT T lymphocytes migrated effectively toward IμHA*Bcl6*-FRC CM (Figure 6D). Neutralization of CXCL9/10 inhibited WT T lymphocyte, but not TIL, migration toward IμHA*Bcl6*-FRC CM, in keeping with densensitization of TIL function by a CXCR3 ligand–enriched FRC secretome (Figure 6D). Thus, these data suggest that altered chemokine signaling in DLBCL-FRCs contributes to a reduced ability to attract TILs.

**Figure 7:**
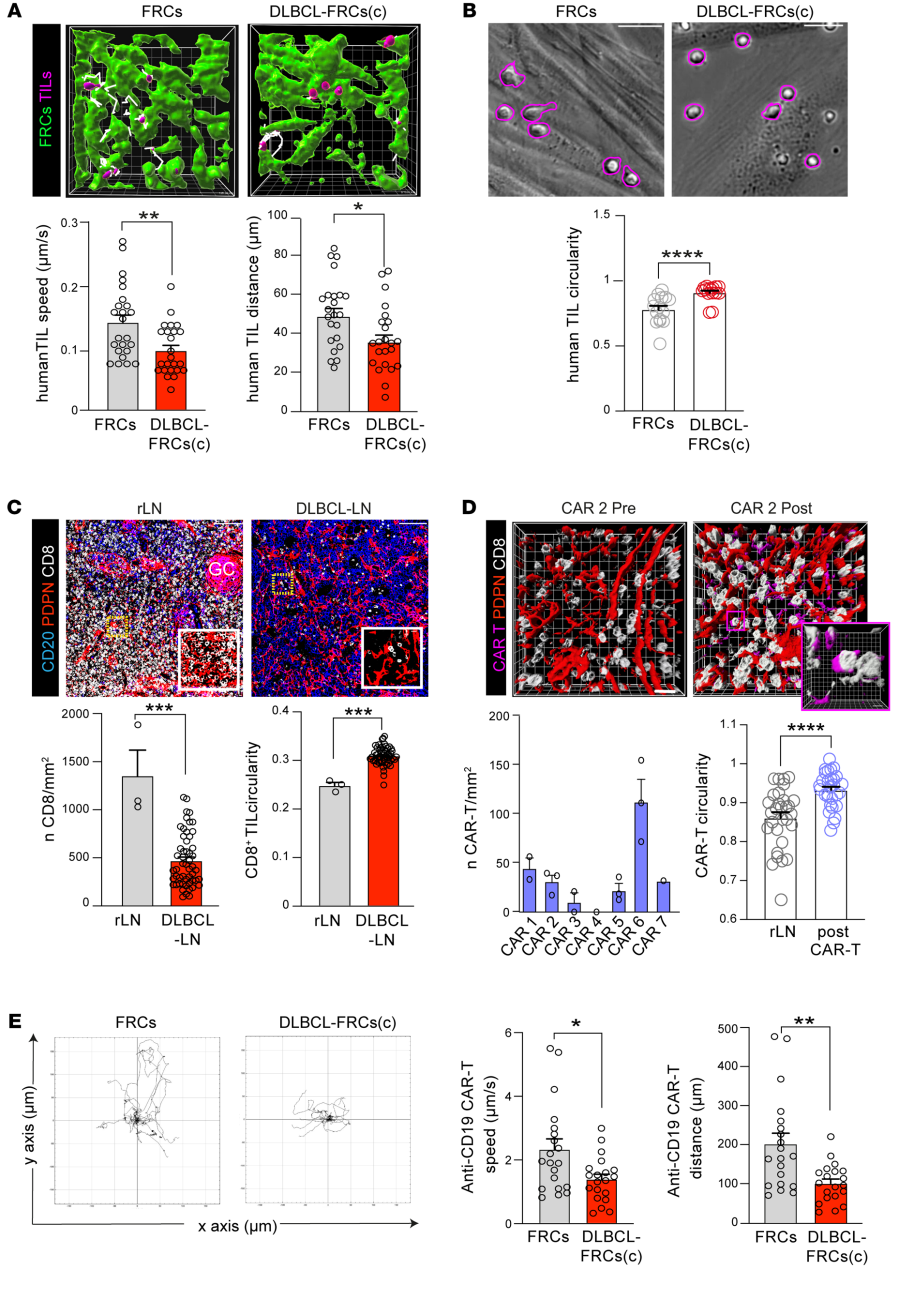
DLBCL-FRCs exhibit a diminished capacity to support T cell and CAR T migration. (**A**) Human 3D TIL motility in gels containing FRCs or DLBCL-FRCs(c) (primary DLBCL cells with autologous TILs) (white lines indicate TIL [purple] migratory tracks). TIL speed and distance quantification. Original magnification, ×20. (**B**) Human TIL cell shape analysis (circularity) during 2D motility on FRCs or DLBCL-FRCs(c) (primary DLBCL cells with autologous TILs) monolayers. Motile TILs (morphology highlighted in purple). TIL cell-shape quantification. (**C**) IF images of stained human rLN (*n* = 5) and DLBCL-LNs (*n* = 15). Scale bars: 100 μm. IMC CD8^+^ T cell numbers/mm^2^ quantification and their circularity in rLN (*n* = 3) and DLBCL-LNs (*n* = 53). (**D**) Images of stained DLBCL-LNs (CAR 2) before and after CAR T cell infusion (CD8^+^ TILs [white], FRCs [red], and CAR T cells [purple]). CD8^+^ CAR T cell numbers/mm^2^ after infusion and their circularity (compared with rLN CD8^+^ cells). (**E**) Anti-CD19 CAR T cell 2D motility on FRCs versus DLBCL-FRCs(c) (SU-DHL16). Left panel, migratory tracks. Right panels, CAR T cell speed and distance quantification. (**A**, **B**, and **E**) Representative patient data from *n* = 3 independent primary DLBCL patient/donor experiments. Data are represented as mean ± SEM (**A**–**E**). *P < 0.05; **P < 0.01; ***P < 0.001; ****P < 0.0001, Mann-Whitney *U* test.

Given the critical function of FRCs in providing a substrate for T cells to migrate upon (26), we next explored how DLBCL-FRCs influenced T cell migratory behavior. Time-lapse imaging revealed that human and murine TILs showed significantly reduced migration when applied to monolayers of DLBCL-FRCs(c) or IμHA*Bcl6*-FRCs, respectively, in comparison with FRCs (Supplemental Figure 6, J and K, Supplemental Videos 1–4). We verified decreased TIL motility along DLBCL-FRC(c) that were coseeded within 3D matrix gels (Figure 7A and Supplemental Videos 5 and 6). Notably, we observed that T cells migrating on DLBCL-FRCs exhibited a more rounded cell shape compared with an elongated morphology and faster movement on FRCs (Figure 7B and Supplemental Figure 6L), in line with a previous study linking T cell shape with migratory speed (47). Our transcriptome analysis of DLBCL-FRCs identified *ICAM1* as a DEG, a ligand for LFA-1 expressed on T cells, that when engaged provides substrate friction during migration (47). We confirmed that human and murine FRCs upregulated ICAM-1 expression following exposure to DLBCL or inflammatory cytokines (Supplemental Figure 6M). The pretreatment of DLBCL-FRCs(c) with a titrated ICAM-1–blocking antibody significantly increased T cell migration, in keeping with elevated adhesion slowing down lymphocyte migration (Supplemental Figure 6N). Additionally, we performed adhesion assays using FRCs as a substrate and observed higher numbers of T cells adhering to DLBCL-FRCs(c) compared with FRCs (Supplemental Figure 6O). Together, these findings suggest that reduced support of T cell migration by DLBCL-FRCs could promote deficient infiltration within the TME.

To investigate the localization of in situ CD8^+^ TILs, we performed IMC and confocal microscopy analysis of human and murine DLBCL tumors. In contrast with clearly demarcated CD8-rich T cell zones in nonmalignant lymphoid tissues, we detected a loss of T cell compartmentalization and significantly reduced numbers of CD8^+^ TILs in DLBCL that localized with the remodeled FRC network (Figure 7C and Supplemental Figure 6P). Further analysis revealed that CD8^+^ T cells exhibited a more circular morphology (Figure 7C and Supplemental Figure 6Q) and increased interactions with FRCs in DLBCL-LNs compared with rLN (Supplemental Figure 6R), consistent with the motility assay data. Given the potential relevance for CAR T cell immunotherapy, we also examined pre- and postinfusion biopsies from a phase I/II trial testing a CD19-targeted CAR T cell product in relapsed/refractory (R/R) DLBCL (48) (Supplemental Table 5). This analysis confirmed the presence of remodeled FAP^+^ FRCs prior to and following treatment, except for 1 patient (CAR 5) who experienced a partial response and whose FRCs formed a more interconnected network after CAR T cell biopsy (Supplemental Figure 6, S–U). Notably, we found that CAR T cells poorly infiltrated DLBCL biopsies (Figure 7D and Supplemental Figure 6V), in agreement with a previous study (20). However, most infiltrating CAR T cells established contact with the remodeled FRC network and exhibited a more rounded cell shape, suggestive of slower movement (Supplemental Figure 6W and Figure 7D). Indeed, time-lapse microscopy confirmed that anti-CD19 CAR T cells showed significantly reduced motility along DLBCL-FRCs(c) compared with elongated faster movement on FRCs (Figure 7E and Supplemental Figure 6X), further supporting that FRCs in DLBCL show a reduced ability to promote T lymphocyte migration.

### DLBCL-FRCs suppress antitumor CD8^+^ T cell cytolytic activity.

**Figure 8:**
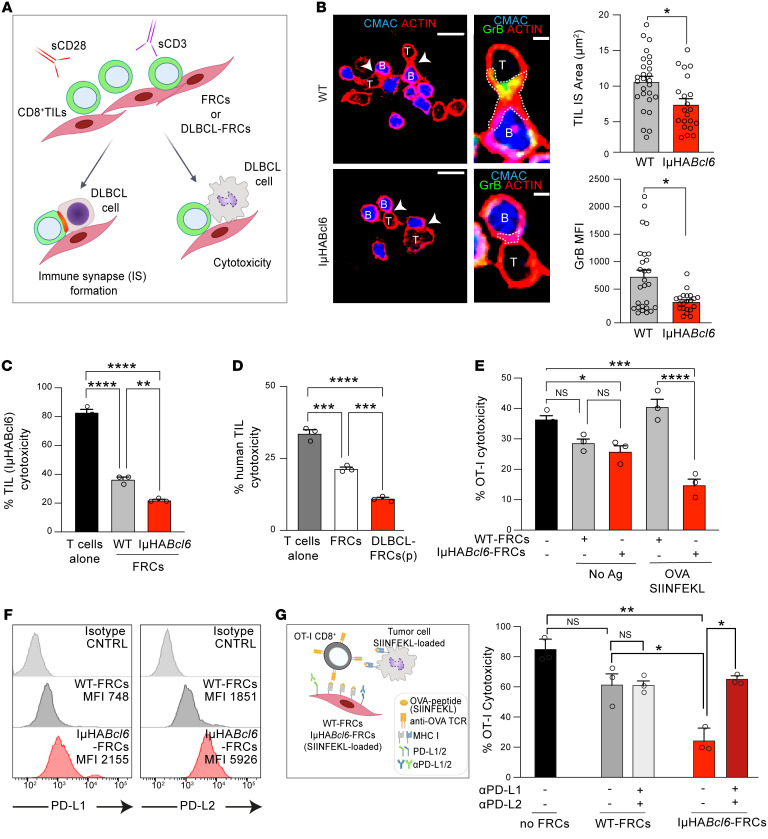
DLBCL-FRCs dampen CD8^+^ TIL killing function. (**A**) Autologous assay schematic assessing CD8^+^ TIL antitumor activities following exposure (24 hours) to DLBCL-FRCs or FRCs. (**B**) CD8^+^ TIL (IμHA*Bcl6*) (T) and DLBCL B cells (B) immune synapse following the exposure of TILs to WT-FRCs or IμHA*Bcl6*-FRCs. Scale bars: 100 μm; 25 μm (magnified). CD8^+^ TIL:DLBCL F-actin^+^ immune synapse (IS) area and GrB MFI. (**C**) TIL (IμHA*Bcl6*) cytotoxicity against DLBCL B cells. TILs activated alone or with WT-FRCs or IμHA*Bcl6*-FRCs. (**D**) Human anti-DLBCL TIL cytotoxicity. TILs activated alone or with FRCs or DLBCL-FRCs(p). TILs, DLBCL cells, and DLBCL-FRCs(p) were autologous (representative patient data from *n* = 2 independent patient samples, ABC and GCB-DLBCL). (**E**) OT-I T lymphocyte cytotoxicity against IμHA*Bcl6* DLBCL cells loaded with SIINFEKL. OT-I exposed to FRCs pulsed with (+) or without (–) SIINFEKL before cytotoxicity assays. (**F**) Histograms of PD-L1 and PD-L2 expression on WT-FRCs (gray, *n* = 3) or IμHA*Bcl6*-FRCs (red, *n* = 5). (**G**) Schematic shows pretreatment of FRCs with anti–PD-L1/PD-L2 in the antigen-specific model. OT-I–mediated anti-DLBCL cytotoxicity. (**B**, **C**, **E**, and **G**) Representative data from *n* = 3 independent sample experiments. Data are represented as mean ± SEM (**B**-**D**, **E**, and **G**). *P < 0.05; **P < 0.01; ***P < 0.001; ****P < 0.0001, 1-way ANOVA with Tukey’s test (**C**–**E**, and **G**) or Mann-Whitney *U* test (**B**).

**Figure 9:**
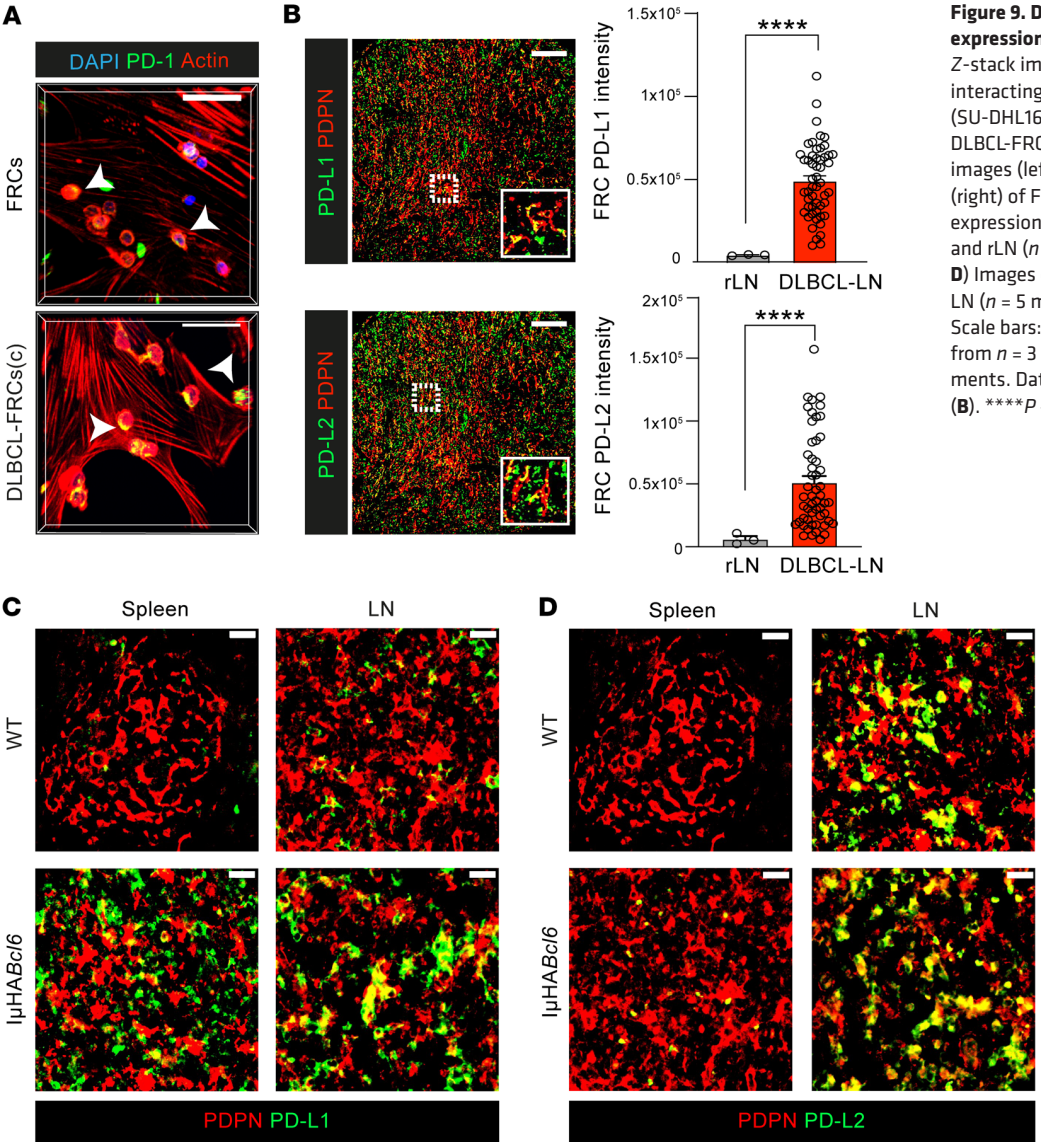
DLBCL-FRCs show aberrant expression of coinhibitory ligands. (**A**) *Z*-stack images of activated T lymphocytes interacting with FRCs or DLBCL-FRCs(c) (SU-DHL16). Polarized PD-1 expression in DLBCL-FRCs(c). Scale bars: 50 μm. (**B**) IMC images (left) and pixel intensity analysis (right) of FRC-associated PD-L1 and PD-L2 expression in human DLBCL-LNs (*n* = 53) and rLN (*n* = 3). Scale bars: 100 μm. (**C** and **D**) Images of WT and IμHA*Bcl6* spleens and LN (*n* = 5 mice/group) stained as indicated. Scale bars: 100 μm. (**H**) Representative data from *n* = 3 independent sample experiments. Data are represented as mean ± SEM (**B**). ****P < 0.0001, Mann-Whitney *U* test.

Enhanced interaction with CD8^+^ T cells and elevated expression of antigen-presentation genes prompted us to investigate how DLBCL-FRCs modulated CD8^+^ T cell cytotoxicity. Flow cytometric analysis confirmed that DLBCL-FRCs(p) and IμHA*Bcl6*-FRCs expressed elevated levels of MHC molecules and could capture and proteolytically process the model antigen OVA (Supplemental Figure 7, A–C). Notably, IμHA*Bcl6*-FRCs exhibited increased crosspresentation of processed MHC class I–associated OVA_257-264_ peptide (SIINFEKL), in keeping with an enhanced antigen-presentation capacity (Supplemental Figure 7D). To investigate modulation of CD8^+^ T cell function, we utilized autologous culture assays that allowed activated CD8^+^ TILs to directly interact with FRCs for 24 hours prior to the addition of DLBCL B cells to measure antitumor activities (Figure 8A). This culture time point preceded the ability of FRCs to attenuate T cell proliferation (27, 49), and we confirmed that culture with FRCs did not alter TIL numbers or viability (data not shown). CD8^+^ T cells form cytolytic immune synapses with target cells to enable polarized secretion of lytic granules and killing of tumor cells. Confocal analysis showed that murine and human CD8^+^ TILs that had been cultured with IμHA*Bcl6*-FRCs or DLBCL-FRCs(p), respectively, displayed a reduced ability to form F-actin–rich, granzyme B^+^ (GrB) lytic synapses with tumor B cells when compared with FRC-educated TILs (Figure 8B and Supplemental Figure 7E). Triple-culture autologous assays revealed that anti-DLBCL T cell–mediated cytotoxicity was significantly reduced when murine CD8^+^ TILs were exposed to IμHA*Bcl6*-FRCs, but not WT-FRCs (Figure 8C). We confirmed this finding in human DLBCL and found that TILs exhibited suppressed antitumor killing function following exposure to DLBCL-FRCs(p) compared with FRCs (Figure 8D). We next assessed whether this inhibition was antigen dependent by adapting our triple-culture assay to incorporate OVA-specific OT-I CD8^+^ T cells interacting with OVA-loaded DLBCL-FRCs, prior to the addition of OVA-loaded DLBCL cells. IμHA*Bcl6*-FRC–driven suppression of OT-I T cell cytolytic function was only detected when FRCs displayed OVA (Figure 8E), consistent with antigen-driven suppression. FRCs have been shown to restrain T cells under inflammatory conditions via upregulated expression of self-antigens and coinhibitory molecules such as PD-L1 (50, 51). Flow cytometric analysis revealed that IμHA*Bcl6*-FRCs expressed increased levels of PD-L1 and PD-L2 in comparison with WT-FRCs (Figure 8F). This led us to test the role of these inhibitory ligands in our culture assay. Pretreating OVA-loaded IμHA*Bcl6*-FRCs with blocking antibodies against PD-1 ligands significantly increased the subsequent cytotoxic function of OT-I T cells against OVA-DLBCL cells, whereas the treatment of WT-FRCs had no effect (Figure 8G). Indeed, blockade of these inhibitory ligands on IμHA*Bcl6*-FRCs augmented endogenous TIL cytotoxicity against autologous DLBCL cells (Supplemental Figure 7F). We further verified these findings in human disease, confirming that exposure to DLBCL cells (or treatment with LTs and TNF-α) triggered increased expression of PD-1 ligands on FRCs (Supplemental Figure 7G). Consistent with these results, immunostaining detected significantly increased focal PD-1 expression on T cells interacting with DLBCL-FRCs(c) compared with FRCs (Figure 9A and Supplemental Figure 7H). Moreover, we detected upregulated expression of PD-L1/2 on in situ FRCs in both human and murine DLBCL in comparison with nonmalignant tissues (Figure 9, B–D, and Supplemental Figure 7I). Our IMC analysis revealed heterogeneous expression of these PD-1 ligands on FRCs in human DLBCL biopsies (Supplemental Figure 7J). Together, these results provide functional evidence that lymphoma-activated FRCs can dampen CD8^+^ T cell–killing function during antigen-dependent interactions via the aberrant expression of coinhibitory ligands.

### CD8^+^ T cell–FRC composition, spatial interaction, and association with survival.

**Figure 10:**
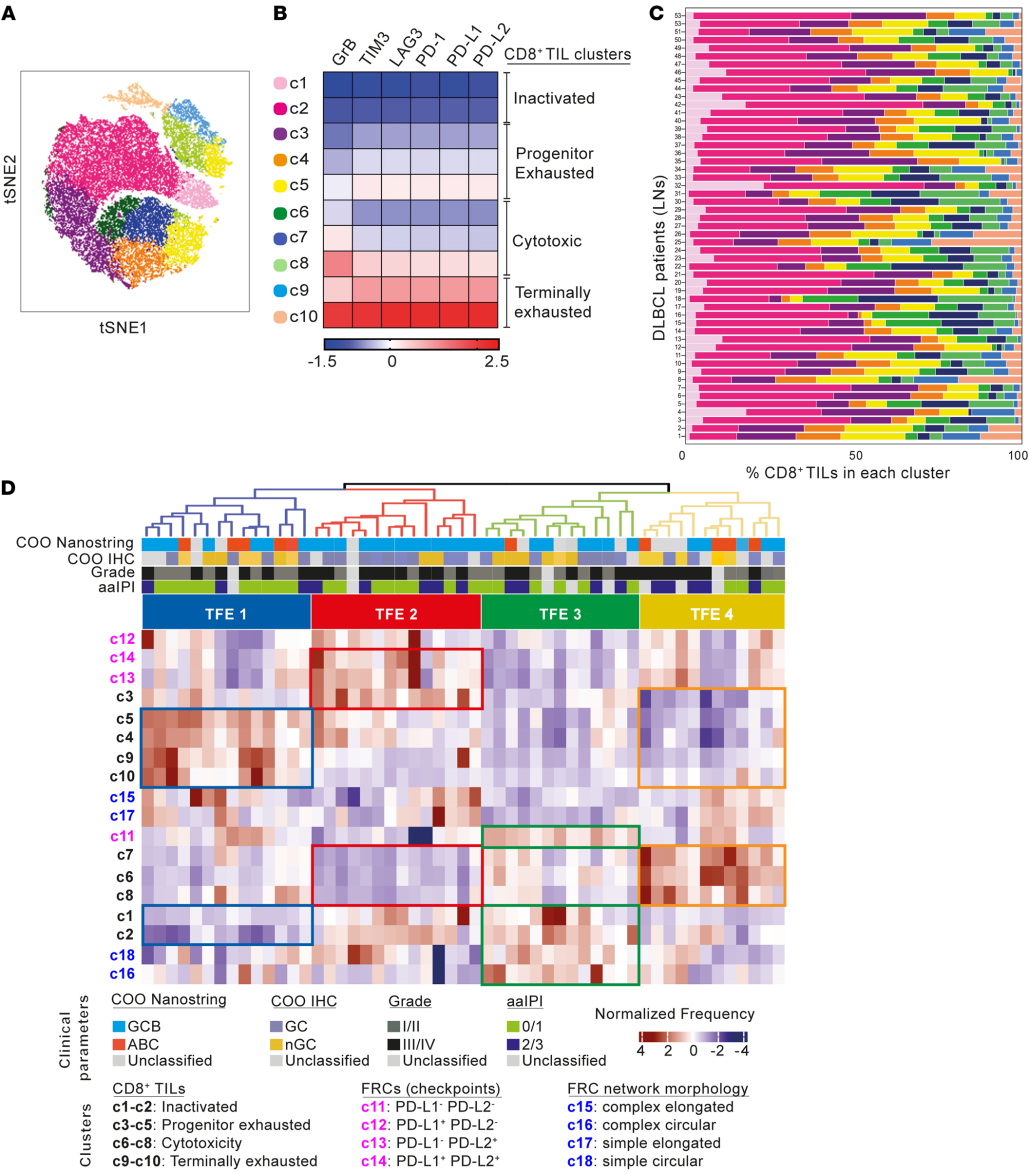
IMC reveals distinct CD8^+^ TFEs in DLBCL. (**A**) *t*-SNE plot of CD8^+^ TILs from 53 DLBCL-LN core biopsies (2 per patient tissue) (IMC). TILs are clustered based on the expression of PD-1, LAG-3, TIM-3, PD-L1, PD-L2, and GrB. (**B**) Heatmap of the median normalized protein expression per CD8^+^ cluster (c1–c10) and associated phenotypic identities indicated. (**C**) Frequency distribution of the identified CD8^+^ clusters across the 53 DLBCL-LNs (TMA patient IDs shown). (**D**) Hierarchical clustering of DLBCL patient data (*n* = 53) based on the z-scored frequency of each CD8^+^ TIL cluster (c1–c10), FRC PD-1 ligand expression cluster (c11–c14) (Supplemental Figure 7J), and FRC morphological shape cluster (c15–c18) (Supplemental Figure 1H). Four CD8^+^ TFEs (TFE1–4) are indicated at the top of the heatmap.

We next sought to further characterize the composition and organization of the CD8^+^ TIL and FRC environment (TFE) within human DLBCL by examining high-dimensional images generated from the TMA with our IMC panel (Supplemental Tables 1 and 2). First, to gain insight into the phenotypic diversity of the CD8^+^ T cell compartment, segmented CD8^+^ single cells were analyzed for the expression of immune checkpoint (PD-1, PD-L1, PD-L2, LAG-3, TIM-3) and cytolytic (GrB) proteins. Phenograph clustering and visualization with *t*-Sne revealed the presence of 10 different CD8^+^ clusters (Figure 10A and Supplemental Figure 8A). To assign putative phenotypic identity, we calculated the normalized median expression of each marker within each cluster and visualized values in a heatmap that enabled the grouping of subpopulations with similar immunophenotypes (Figure 10B). This analysis highlighted the gradual phenotypic diversity of the intratumoral CD8^+^ T cell pool in DLBCL that expressed various levels of coinhibitory checkpoints. Two of these clusters (c1 and c2) displayed a checkpoint^lo^ and GrB^lo^ immunophenotype in keeping with a nonactivated T cell state (52); c3, c4, and c5 showed an immunophenotype reminiscent of intratumoral progenitor exhausted CD8^+^ T cells (53), characterized by higher expression of checkpoint molecules compared with GrB (54). In contrast, higher levels of GrB expression compared with checkpoints in c6, c7, and c8 were consistent with cytotoxic T cells (52). The remaining 2 clusters (c9 and c10) displayed a checkpoint^hi^ and GrB^hi^ immunophenotype, characteristic of terminally exhausted CD8^+^ T cells (52, 53). Notably, the frequency of each CD8^+^ subpopulation (Figure 10C) did not correlate with COO. We then verified the phenotypic identities obtained with a phenograph by analyzing DLBCL patient TILs with flow cytometry (Supplemental Figure 8, B and C), confirming the presence of dysfunctional PD-1^+^TIM-3^+^ TILs in DLBCL (52, 54, 55).

**Figure 11:**
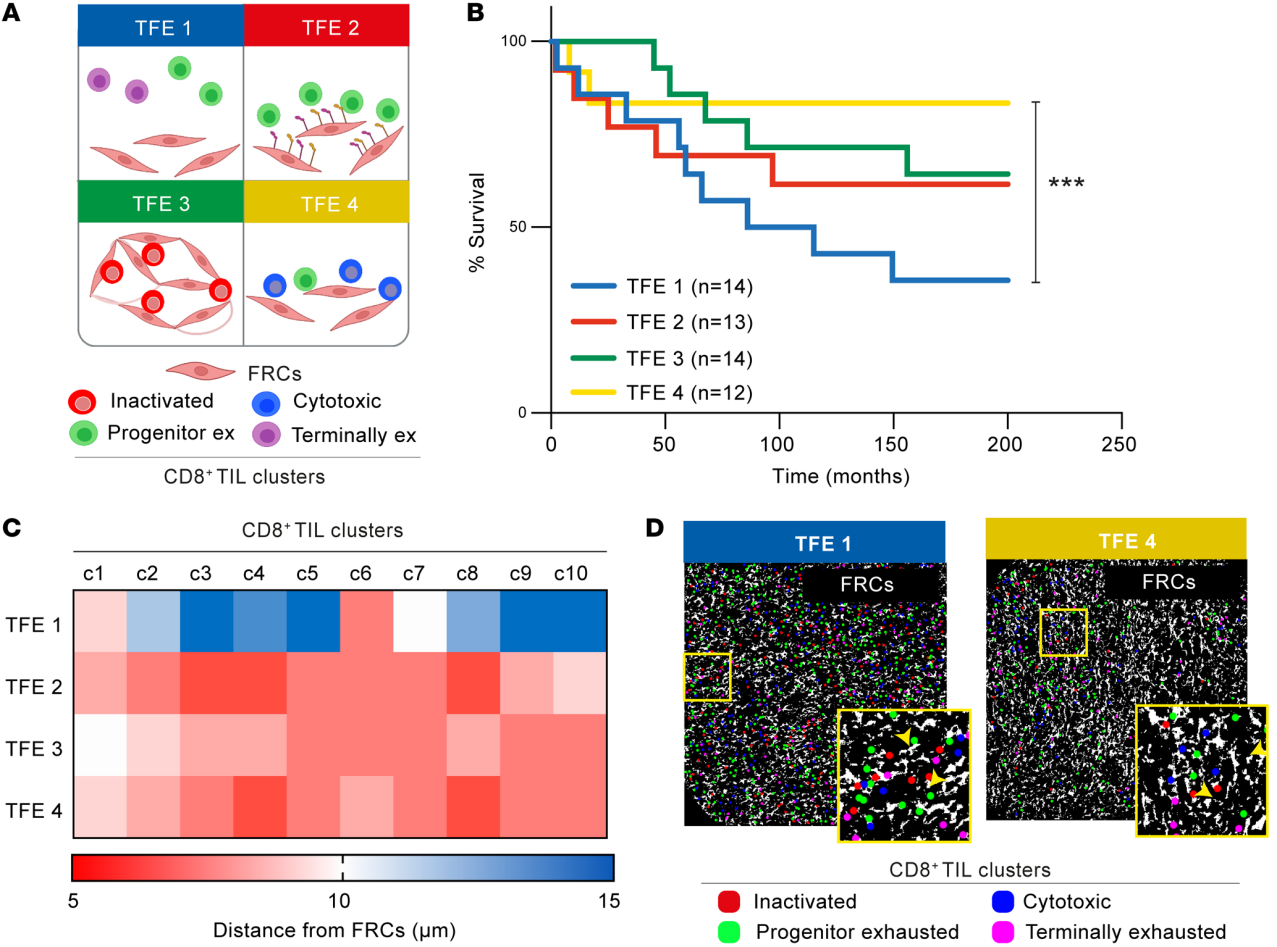
CD8^+^ TIL/FRC spatial organization associates with survival outcome in DLBCL. (**A**) TFEs in the DLBCL-LN TMA schematic. CD8^+^ TIL phenotypic identities: inactivated (red), progenitor exhausted (green), cytotoxic (blue), and terminally exhausted (purple). (**B**) Kaplan-Meier curves of overall survival for each identified TFE (*n* = number of patients per TFE group). (**C**) Heatmap showing the average distance of each CD8^+^ cell (within CD8^+^ TIL clusters, c1–c10) from the FRC network for each TFE. (**D**) Two representative DLBCL-LN samples belonging to TFE 1 and TFE 4, showing the FRC mask (white) and the center of each CD8^+^ phenotypic cluster: inactivated (red), progenitor exhausted (green), cytotoxic (blue), terminally exhausted (pink). Original magnification, ×20. (**B**) ***P < 0.001, log-rank (Mantel-Cox) test.

Next, we performed unsupervised hierarchical clustering of CD8^+^ TIL and FRC subpopulation (Supplemental Figure 1L and Supplemental Figure 7J) frequencies within all 53 tumors and identified 4 distinct TFEs (Figure 10D, Figure 11A, and Supplemental Figure 8D). TFE1 was characterized by a relatively higher proportion of exhausted (progenitor and terminally) but lower cytotoxic and nonactivated CD8^+^ TILs. In contrast, TFE4 DLBCLs were enriched in cytotoxic CD8^+^ T cells but lower in exhausted CD8^+^ TILs. TFE2 contained a higher proportion of PD-1 ligand^+^ FRCs and progenitor exhausted CD8^+^ T cells, but relatively fewer cytotoxic TILs. Finally, less activated FRCs (circular and PD-1 ligand^–^) and nonactivated T cells were enriched in TFE3. We found no association of these TFEs with COO or known clinical parameters. Remarkably, however, patients from TFE1 showed significantly shorter survival than those in TFE4, who had superior survival outcomes, whereas TFEs 2 and 3 showed intermediate stratified survival outcomes (Figure 11B).

Finally, to gain insight into TIL-fibroblast spatial organization within these TFEs, we conducted distance analysis between CD8^+^ clusters and the FRC network. Strikingly, in contrast with close CD8^+^ T cell/FRC interactions observed in intermediate and good outcome tumors (TFEs 2, 3, and 4), spatial analysis revealed an uncoupling of CD8^+^ clusters from the total FRC network (>10 μm distance) in the unfavorable TFE1 group (Figure 11, C and D). Overall, our IMC analysis provides evidence that the makeup of the CD8^+^ TIL/FRC network and their interactivity could help define distinct TMEs that associate with survival.

### Reinvigorating antilymphoma TIL activity with tumor- and FRC-targeted combination immunotherapy.

**Figure 12:**
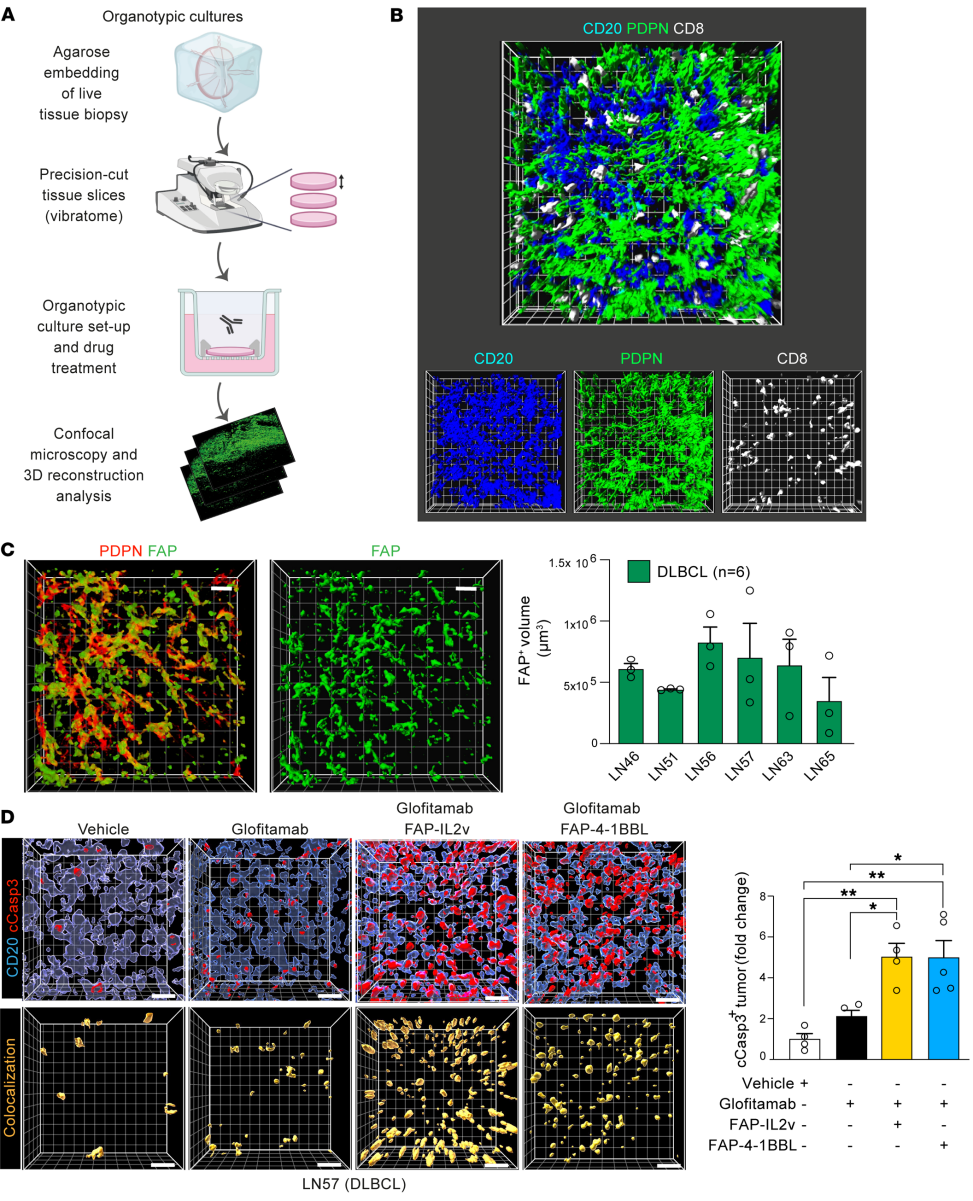
Combining FRC-targeted immunotherapy with glofitamab enhances antitumor activity in organotypic cultures. (**A**) 3D precision-cut LN slice-based organotypic cultures schematic. (**B**) Representative 3D image reconstruction of a human lymphoma organotypic culture stained for DLBCL cells (CD20), FRCs (PDPN), and TILs (CD8). Original magnification, ×20. (**C**) Confocal analysis of in situ FRCs (DLBCL-LN organotypic culture) stained for PDPN and FAP (left, 3D images; right, volume occupied analysis of FAP^+^ FRCs, *n* = 6 DLBCL patient LNs). (**D**) DLBCL organotypic cultures (LN57) treated for 48 hours with control antibodies (vehicle: DP47-TCB, DP47-4-1BBL, FAP-PGLALA) or with glofitamab (CD20xCD3) alone or in combination with FAP-IL2v or FAP-4-1BBL. 3D volume-rendered images show CD20^+^ tumor cells and cleaved caspase-3 (cCasp3) staining (upper images) and the colocalization channel (CD20^+^/c-Casp3^+^ cells) (lower). Cleaved caspase-3^+^ tumor cells (fold change quantification compared with vehicle treatment). Data are represented as mean ± SEM (**C** and **D**). *P < 0.05; **P < 0.01, 1-way ANOVA with Tukey’s multiple-comparisons test. Scale bars: 15 μm.

**Figure 13:**
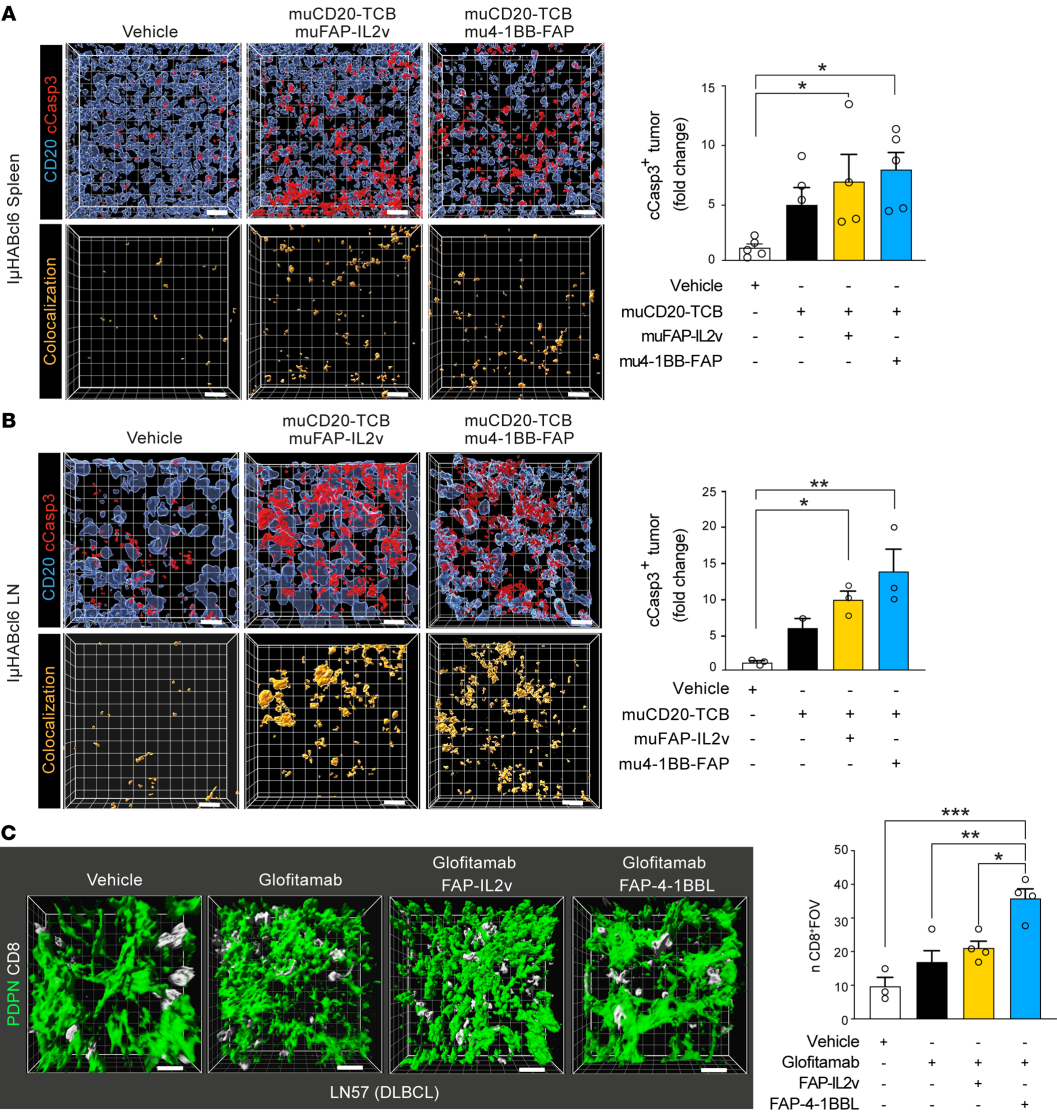
Combination immunotherapy enhances T cell retention in organotypic cultures. (**A** and **B**) Representative IμHA*Bcl6* spleen (**A**) and LN (**B**) organotypic cultures treated for 48 hours with vehicle or with surrogate murine (mu) muCD20-TCB alone or in combination with muFAP-IL2v or mu4-1BB-FAP immunotherapy. Cleaved caspase-3^+^ DLBCL cells (fold change quantification compared with vehicle). Data show 1 experiment (from *n* = 3 independent mice). (**C**) 3D confocal reconstruction of in situ FRCs and CD8^+^ TILs in a DLBCL (LN57) organotypic culture treated for 48 hours with the drugs indicated. Number of CD8^+^ TILs per field of view. Data are represented as mean ± SEM (**A**–**C**). *P < 0.05; **P < 0.01; ***P < 0.001, 1-way ANOVA with Tukey’s multiple-comparisons test. Scale bars: 15 μm.

Finally, to assess whether the interaction between TILs and inhibitory stromal cells could be targeted to stimulate antitumor immune activity, we explored the potential to harness FAP-expressing FRCs with targeted immunotherapy utilizing patient-derived samples. TCBs designed to redirect TILs, such as glofitamab (CD20 × CD3), are showing clinical promise in R/R lymphoma (56, 57), with the added potential to pair with costimulatory agonists to enhance T cell responses (58, 59). To investigate whether an FAP-targeted 4-1BB agonist (FAP-4-1BBL, RG7827) or IL-2 variant immunocytokine (FAP-IL2v, simlukafusp alfa) could target in situ FAP^+^ myofibroblasts to augment glofitamab-triggered TIL functionality, we established a precision-cut LN tissue slice organotypic culture assay. Advantageously, this system preserves the intact immune-FRC TME for subsequent 3D image analysis (Figure 12, A and B). We detected marked FAP expression on the FRC network in the 6 DLBCL biopsy tissues studied, which included diagnostic and R/R disease (Supplemental Table 6, Figure 12C, and Supplemental Figure 9A). Tumors were treated with glofitamab alone or in combination with FAP-IL2v or FAP-4-1BBL for 48 hours. We verified that glofitamab stimulated the expression of 4-1BB on CD8^+^ TILs in the lymphoma TMEs (Supplemental Figure 9B), creating an opportunity for 4-1BB agonist activity. Although glofitamab induced a nonsignificant increase in tumor cell death, its pairing with either FAP-IL2v or FAP-4-1BBL resulted in augmented DLBCL cell death compared with untargeted control drugs in all the lymphoma tissues tested — suggesting that the levels and distribution of FAP in human DLBCL is sufficient to enable target-mediated costimulation of interacting TILs (Figure 12D) (Supplemental Figure 9, C–G). We also evaluated the targeting of FAP^+^ FRCs in IμHA*Bcl6* tumors using murine (mu) surrogate antibodies (58) with splenic and LN slice organotypic cultures that overcame the low-penetrance challenge of this model and allowed comparative treatment studies. In keeping with the human data, combining a muCD20-TCB with mu4-1BB-FAP or muFAP-IL2v significantly improved DLBCL cell killing compared with TCB treatment alone (Figure 13, A and B and Supplemental Figure 9, H and I). Assessment of T cell retention in the human tumor cultures showed an increased persistence of CD8^+^ T cells following combination immunotherapy, in keeping with activation of cytolytic synapses (Figure 13C and Supplemental Figure 9, C–G). In contrast, the treatment of a rLN lacking FAP expression on the reticular network showed insensitivity to the addition of these stroma-targeting drugs (Supplemental Figure 9J).

To investigate FRC-suppressive activity in response to immunotherapy-induced inflammation, we measured the expression of PD-1 ligands on in situ FRCs and found significantly increased levels following glofitamab treatment — suggesting that FRCs could restrain immunotherapy-activated TILs (Supplemental Figure 10A). Indeed, in vitro triple-culture assays showed that glofitamab-triggered TIL cytotoxicity was significantly lower in the presence of DLBCL-FRCs(c) compared with FRCs (Supplemental Figure 10B). In keeping with this finding, we detected reduced IL-2, IFN, and GrB in the CM harvested from glofitamab-treated cultures (Supplemental Figure 10C). Importantly, the combination of FAP-targeted immunostimulatory drugs with glofitamab increased anti-DLBCL TIL killing function when TILs were interacting with FAP^+^ DLBCL-FRCs(c), but not FRCs lacking detectable FAP expression (Supplemental Figure 10D). Consistent with these findings, the cotreatment of DLBCL-LN slices with blocking anti–PD-L1/2 antibodies enhanced the antitumor activity of glofitamab to a level comparable to that of combination FAP-4-1BBL/FAP-IL2v plus glofitamab — supporting the ability of FAP-targeted immunotherapies to counteract inhibitory signaling from FRCs (Supplemental Figure 10E). In addition, given our earlier finding of diminished expression of homeostatic chemokines in DLBCL-FRCs, we sought to investigate the impact of immunotherapy. Immunoassays revealed enrichment of CCL19 and CCL21 within the CM of in vitro cultures treated with glofitamab (Supplemental Figure 10F). Analysis of treated DLBCL-LNs verified significantly increased expression of FRC-associated CCL21, suggesting promotion of a T cell–attracting TME (Supplemental Figure 10G). Indeed, chemotaxis assays showed that the CM of glofitamab-treated triple-culture assays increased the recruitment of untreated TILs (Supplemental Figure 10H). Finally, we assessed targeting FAP^+^ FRCs in other CD20-expressing B cell malignancies and confirmed that the addition of FAP-targeted immunostimulatory drugs could augment glofitamab-induced cytotoxicity in follicular lymphoma and Hodgkin lymphoma patient LNs when FAP was expressed by stromal cells (Supplemental Table 6 and Supplemental Figure 10, I–L). Together, these results suggest that lymphoma-activated FRCs are inhibitory toward CD8^+^ TILs and immunotherapy, but can be targeted with stroma-targeted immunotherapy to improve antitumor immune activity.

## Discussion

The immunological consequences of lymphoma development on the ability of LNs to maintain homeostasis and regulate immune responses remain unclear. Such knowledge could be harnessed to better understand immunosuppressed TMEs in NHL and to optimize immunotherapy. Recent transcriptomic-based classification of DLBCL TMEs has highlighted the relevance of fibroblast-immune cell landscapes (12–14), but functional studies to define immunobiology have been lacking. Here, we have leveraged primary patient samples and a murine model of disease to reveal that exposure of LN-resident immunologically specialized FRCs to DLBCL B cells triggers their activation, remodeling, and reprogramming. DLBCL-exposed FRCs exhibited an altered immunoregulatory state that includes a switch from homeostatic to inflammatory chemokine expression as well as upregulated adhesion and antigen-presentation molecules. Our functional studies suggest that altered DLBCL-FRCs impeded optimal T cell migration and inhibited CD8^+^ T cell lytic function. Despite these suppressive properties, we demonstrate that FRCs in lymphoma TMEs can be cotargeted in combination with immunotherapy to boost TIL cytolytic activity.

FRCs have a dual role in enhancing immune activation while also suppressing T cell responses (24, 25, 28, 60). This seemingly paradoxical activity reflects the crucial role FRCs play in dynamically regulating optimal immune responses during homeostasis, inflammation, and subsequent resolution. During an immune response, the FRC network remodels, alters transcriptionally, and proliferates to allow LN expansion and support increased immune cell infiltration and function (31, 34, 40). Importantly, after pathogenic clearance, FRCs contract this network and normalize their inflammation-altered transcriptome to allow the LN to return to homeostasis (31). Here, we demonstrate that signals from DLBCL B cells activate FRCs and lead to reprogramming of key inflammatory and immunomodulatory pathways. We reveal that lymphoid tissues burdened with DLBCL cells contained an expanded and heavily remodeled FRC network, resembling an amplified acute immune response state (31). In common with how FRCs respond to CLEC2-expressing DCs during an immune response (32), we observed that exposure of FRCs to DLBCL cells led to an inhibition of their contractile signaling and induced stretching. We found that DLBCL-derived inflammatory molecules TNF, LT3, and LT12 activated FRCs, while FRC-expressed BAFF supported DLBCL B cell survival. In line with previous reports of normal and malignant B cell bidirectional crosstalk with stromal cells (36, 61–63), we propose a model whereby infiltrating DLBCL B cells sustain chronic inflammatory FRC activation within LN TMEs by hijacking B cell–derived signals, which are normally spatially and temporarily controlled during immune reactions. Although blocking these TNF family molecules was sufficient to attenuate tumor-induced FRC stretching, this did not fully abrogate fibroblast activation, suggesting that DLBCL cells produce additional stromal remodeling factors (13). DLBCL-FRCs exhibited a profoundly altered transcriptional state, resembling the activation of innate immune sensors in FRCs following viral infection (24, 26, 31, 40). Accordingly, we detected altered expression patterns of genes involved in proliferation, metabolism, ECM remodeling, inflammatory type I and II IFN signaling, cytokine and chemokine signaling, and antigen presentation. Importantly, these transcriptional pathways were found in FRCs following acute exposure to DLBCL B cells in coculture assays, but also in FRCs expanded from patient or murine tumors. Additionally, scRNA-Seq analysis of murine DLBCL-LNs revealed a reduction in the abundance of *Ccl19*-expressing TRCs that normally attract T cells and DCs through the provision of CCL19 and CCL21 (43). In contrast, we detected an expansion of MedRCs in diseased LNs, which provide niche factors including BAFF and IL-6 for plasma B cells during an immune response (64). Moreover, a collagen-producing FRC subset (*Col15a1*^+^*Smoc2*^+^*Cd34*^+^ fibroblasts) was also expanded in DLBCL, consistent with enriched fibroblast-derived ECM signatures recently described in DLBCL TMEs (13). Our transcriptome analysis revealed downregulation of chemokines *Ccl21* and *Ccl19*, while IFN-regulated chemokines such as *Cxcl9* and antigen-presentation genes were upregulated across multiple FRC subsets within DLBCL-LNs. Collectively, these data suggest that DLBCL reprograms FRCs into a chronically activated CAF-like state that could foster the tumor niche and promote aberrant immunity.

Additionally, our study utilized tumor-derived primary cultures to demonstrate that DLBCL-FRCs could interfere with T cell function. We confirmed that the FRC network in DLBCL tissues had a deficiency in homeostatic CCL21 and CCL19 expression. Accordingly, CM harvested from DLBCL-FRCs showed a diminished ability to attract TILs in chemotaxis assays. Unexpectedly, IFN-induced chemokines produced by DLBCL-FRCs failed to offset reduced TIL migration, which we hypothesize could be related to CXCR3 desensitization within the inflammatory TME. Our data are consistent with a previous murine model study demonstrating that lymphoma-induced high endothelial venule and FRC remodeling was detrimental for T cell transmigration into malignant LNs (65). Furthermore, we observed decreased TIL and CAR T cell motility along DLBCL-FRCs using 2D and 3D assays. Based on our data, we speculate that elevated ICAM-1 expression on lymphoma-activated FRCs could exert adhesive breaks for migrating lymphocytes. Our analysis of TME tissues supported this view, as CD8^+^ TILs, although low in number, showed increased interactions with remodeled FRCs compared with nonmalignant LNs. Our functional assays also revealed the ability of DLBCL-FRCs to suppress interacting CD8^+^ T cells. Increased expression of antigen presentation and coinhibitory molecules PD-L1 and PD-L2 by DLBCL-FRCs inhibited antitumor CD8^+^ cytolytic function in an antigen-dependent manner. Our data identify fibroblasts as a functionally relevant PD-L1/2–expressing cellular compartment within the DLBCL TME (66). Together, these data suggest that FRCs in DLBCL are held in an unresolved inflammatory state that promotes immunosuppressive properties. Intriguingly, this mechanism is akin to inhibitory FRC function during chronic viral infection (51) and solid tumor CAF-mediated suppression and deletion of CD8^+^ T cells (67). Although we did not detect any modulation of T cell viability by FRCs in our short-term culture assays, we do not exclude that chronic exposure of TILs to proinflammatory and immunosuppressive signals from fibroblasts in the TME could modulate their survival or differentiation states (41, 68). For example, murine FRCs have been shown to produce nitric oxide (27), while human FRCs express multiple inhibitory molecules including IDO and TGF to keep the proliferation of activated T cells in check (28). Additional studies will be needed to determine the full scale of immunoregulatory activity directed by FRCs in the lymphomas.

Considering the diversity of cellular ecosystems in DLBCL (14), it will be important to understand the relationship between the FRC state and the regulation of innate and adaptive immune processes within the TME. Here we employed high-dimensional image analysis to provide insight into the composition and spatial localization of the CD8^+^ TIL/FRC network in DLBCL-LNs and identified 4 distinct microenvironments (TFEs). TFE4 tumors associated with superior patient survival and were enriched in GrB^hi^CD8^+^ T cells interacting with FRCs. This finding suggests that cytolytic CD8^+^ T cells may be able to overcome the inhibitory action of FRCs in this subset of patients. In contrast, TFE2 tumors with a paucity of cytolytic TILs, but increased frequencies of PD-1 ligand^+^ FRCs and progenitor, exhausted CD8^+^ T cells associated with intermediate survival outcome. Intriguingly, poor-outcome TFE1 DLBCLs harbored an increased proportion of exhausted CD8^+^ TILs, and spatial analysis revealed evidence of T cells dissociating from the FRC network. Given the physiological role of FRCs as dedicated immune-interacting and supporting fibroblasts, we speculate that, in some patients, DLBCL-FRCs may still retain some immunostimulatory activity to support TIL function, whereas when the acquisition of inhibitory properties in tumor-altered FRCs prevail, this could promote adverse immunosuppressed or cold TMEs. Indeed, a decrease in immune and stromal cell gene signatures in DLBCL TMEs toward “depleted” ecosystems is associated with disease progression and poor outcome, whereas enriched FRC-immune gene expression profiles are predictive for better patient survival (12–14). While we have focused on FRC-CD8^+^ T cell interactions, we envision that DLBCL-FRCs also modulate other key immune subsets that reside within the TME, which is supported by our data showing upregulated chemoattractants such as *Ccl2* for macrophages. Additional studies will be required to understand how FRC activation states (as well as other LN stromal cells) influence the immune landscape within evolving lymphoma TMEs.

Our work also provides proof of principle that inhibitory fibroblasts can be targeted in DLBCL and other B cell lymphomas to stimulate endogenous CD8^+^ T cells interacting with the remodeled FRC network. We took advantage of increased FAP expression on DLBCL-FRCs to demonstrate that FAP-targeted immunostimulatory drugs could augment antitumor CD8^+^ T cell activity induced by glofitamab, utilizing patient LN organotypic cultures. Intriguingly, our image analysis hinted at the ability of glofitamab to reprogram FRCs toward a more homeostatic state, as we detected increased CCL21 expression on the FRC network following treatment. Future research should define which FRC-immune crosstalk and topology influences respond to bispecific antibody–based or CAR T immunotherapy and the potential for beneficial stroma-immune reprogramming. Our work reveals the immunological relevance of remodeled FRCs in DLBCL, including the potential to suppress endogenous and immunotherapy-induced T cell responses. This contributes to emerging data recognizing reprogrammed LN fibroblasts as CAFs in the lymphomas and their potential role in shaping the immune TME (14, 62, 69). Further insights into their activation state and diverse immune functions should inform the development of future therapeutic approaches to help optimize immunotherapy for patients.

## Methods

All methods are described in Supplementary Methods.

### Human patient samples: human LNs.

Excess fresh excision diagnostic biopsies from rLNs (*n* = 5) and DLBCL/lymphoma-LNs (*n* = 29) (Supplemental Tables 3 and 6) were processed for the isolation of viable primary lymphocyte cellular suspensions, FRC isolation and culture, and organotypic cultures (see *Primary cell isolation and culture* and *Organotypic cultures* in Supplemental Methods), FRC isolation and culture, and organotypic cultures.

### Data availability.

Data were deposited in the NCBI’s Gene Expression Omnibus database (GEO GSE179161, GSE179193, GSE193565). These and reagents are all detailed in Supplemental Table 7. Values for all data points found in graphs can be found in the Supporting Data Values file.

### Statistics.

Statistical analyses were performed using Prism 9 (GraphPad). Unpaired Mann-Whitney *U* test was performed when 2 data sets were compared. One-way ANOVA with Tukey’s multiple-comparisons was used to compare 3 or more data sets. Survival (overall survival: time from initial diagnosis to death due to any cause) was calculated using Kaplan-Meier estimate with the log-rank (Mantel-Cox) test. *P* values of less than 0.05 were considered significant. Sample size, experimental replicates, and additional details are provided in Supplemental Methods and figure legends.

### Study approval.

All patient samples were obtained with written informed consent, in accordance with the Declaration of Helsinki, and their research use was approved by the National Research Ethics Committee. Murine model work was fully compliant with UK Home Office guidelines and was approved by the UK Home Office.

## Author contributions

BA designed and performed experimental work and data analysis and wrote the manuscript. FS, NP, MTB, and RE performed IMC analysis. PJ and DP assisted in RNA-Seq sample preparation and organotypic culture. SK and RN contributed to sequencing and analysis. DC and MS performed bioinformatic analysis. GE, EP, RR, LAS, GZ, AV, RFJ, AD, CG, and RB contributed study samples and clinical data. RMA and JS provided histopathology analyses. MB, CC, PU, CK, DPC, PRH, and AKG contributed to data interpretation and/or discussion. AGR designed and supervised the study and wrote the manuscript.

## Supplementary Material

Supplemental data

Supplemental tables 1-7

Supplemental video 1

Supplemental video 2

Supplemental video 3

Supplemental video 4

Supplemental video 5

Supplemental video 6

Supporting data values

## Version 1 05/23/2023

In-Press Preview

## Version 2 07/03/2023

Electronic publication
