# Supplementary material for: Tumor-activated lymph node fibroblasts suppress T cell function in diffuse large B cell lymphoma
Source: J Clin Invest. 2023 Jul 3;133(13):e166070. doi: 10.1172/JCI166070 (PMC10313378; doi:10.1172/JCI166070)
Supplement: Supplemental data [file jci-133-166070-s143.pdf]

Tumor-activated lymph node fibroblasts suppress T cell function in diffuse large B cell lymphoma

Supplemental Material

1. Supplemental Methods

2. Supplemental Figures 1-10

3. List of Movies

4. References

Supplemental Methods

Human patient samples.

Formalin-fixed paraffin embedded (FFPE) tissues. Whole tissue FFPE biopsies from 10 control rLNs and 15 DLBCL-LNs at diagnosis (10 GCB, 5 ABC) were obtained from King's College Hospital (London). The TMA containing 53 DLBCL patients (**Supplemental Table 1**) was obtained from Uppsala University and Uppsala University Hospital. The molecular and clinical characteristics of these patients have previously been reported (1). The TMA contained two 1 mm^2 cores from each patient biopsy. DLBCL tissues pre- and post-CAR-T cell treatment were obtained from patients (n=7) enrolled in the EudraCT 2013-001393-19/NCT02132624 clinical trial at Uppsala University Hospital (**Supplemental Table 5**) (2).

Human primary FRC and DLBCL cell lines. Primary human lymphatic fibroblasts derived from non-diseased rLNs were purchased from Sciencell (n=4 donors) as a source of FRCs for all DLBCL-FRC(c) (see FRC conditioning). Immunophenotyping ($CD45^-$, $CD31^-$, $CD21^-$, $PDPN^+$) confirmed FRC identity. They were cultured in Fibroblast Media (Sciencell) according to manufacturer's instructions and used at passage 3 in all assays.

Cell lines used are all summarized in **Supplemental Table 7**. DLBCL cell lines U2932, SU-DHL16, SU-DHL4, OCI-Ly3 were purchased from DMSZ, DLBCL cell lines SU-DHL10, RL, TMD8, WSU-NHL, WSU-DLCL2, OCI-ly1, OCI-Ly10, and OCI-Ly18 were a kind gift from Patrick Hagner (Bristol-Myers Squibb, USA). EBV-transformed normal B lymphocyte lines ('B cell lines') JY, 00136 and 111BLCL were purchased from Public Health England (UK). All the cell lines were cultured in complete RPMI medium (RPMI 1640 (Gibco/Thermo Fisher Scientific) supplemented with 10% fetal bovine serum (FBS) (Gibco/Thermo Fisher Scientific), 100 U/mL penicillin and 100 µg/mL streptomycin (Sigma-Aldrich)), and routinely tested for mycoplasma contamination.

Mice.

IµHA*Bcl6*, line 4E12, (C57BL/6) mice were kindly provided by Prof. Dalla-Favera (Columbia University, USA), with breeding and genotyping performed as described previously (3). Mice were housed in pathogen-free conditions using a positive pressure isolator. Mice were monitored up to 20 months of age for the onset of lymphoma symptoms (palpable and visible splenomegaly, lymphadenopathy). DLBCL diagnosis was confirmed using hematoxylin-eosin (H&E) staining on harvested spleen and lymph node tissues by a pathologist (R.M.A.). IµHA*Bcl6* mice developed lymphoma from 10 months of age onwards with a low penetrance (20.3%). The median time to lymphoma development was 16 months. Age-matched WT mice (littermates, median age 16 months) were used as controls in all assays.

Reagents.

The full summary of reagents and antibodies, dilutions and assay applications are listed in **Supplemental Table 7.**

Drugs.

Glofitamab (CD20-TCB) (human), FAP–4-1BBL (human), FAP-IL2v (Simlukafusp alfa) (human), muFAP-IL2v (murine, mu), muCD20-TCB and mu4-1BB-FAP were designed as previously described (4-6) and provided by Roche Innovation Center Zurich. FAP-PGLALA (anti-FAP IgG1 with P329G, L234A and L235A mutations, clone 4B9) was used as a control ('vehicle') for the FAP-IL2v drugs. Non-binding DP47-TCB was used as control ('vehicle') for CD20-TCB, non-binding DP47-4-1BBL was used as a control ('vehicle') for the FAP-4-1BBL drug. (human/murine) CD20-TCB was used at the concentration of 0.1 µg/mL, FAP-targeting drugs were used at a concentration of 0.5 µg/mL in all assays.

Primary cell isolation and culture

Tumor B cells and T lymphocytes/TILs. Fresh diagnostic human patient DLBCL-LN biopsies (as well as human rLNs) and murine DLBCL diseased spleen samples were cut into small pieces and mechanically dissociated using a metal sieve to obtain viable single-cell suspensions. Cells were centrifuged,

resuspended in complete RPMI 1640 medium and counted. Human and murine CD19$^+$ DLBCL B cells ('DLBCL cells or DLBCL B cells') (or CD19$^+$ B cells from rLNs), CD3$^+$ and CD8$^+$ T cells (human: CD3$^+$ or CD8$^+$ TILs, murine: CD3$^+$ or CD8$^+$ IμHA*Bcl6*-TILs or WT T cells as indicated) were all negatively isolated using column-free magnetic separation kits (StemCell Technologies) according to manufacturer's instructions.

T cells were also purified from the peripheral blood of human healthy donors. Briefly, after isolation of peripheral blood mononuclear cells (PBMCs) by density gradient centrifugation using Ficoll-Histopaque (Sigma), CD3$^+$ and CD8$^+$ T cells were negatively selected using magnetic separation kits (StemCell Technologies) according to manufacturer's instructions.

Anti-CD19 CAR-T cell (cell product for in vitro studies). CD3$^+$ T cells isolated from the peripheral blood of healthy donors were resuspended in 'transduction media' (X-VIVO 15 (Lonza) supplemented with 1x MEM Non-essential Amino Acid Solution (Sigma), 20 mM HEPES (Thermo Scientific), 1mM Sodium pyruvate (Thermo Scientific) and 10% FBS), activated with CD3/28 dynabeads (Thermo Scientific) supplemented with 20 ng/mL IL-2 (Miltenyi Biotec) and transduced with an anti-CD19 CAR (anti-CD19 4G7 scFv 4-1BB CD3ζ) lentivirus (MOI 5) (7, 8). Anti-CD19 CAR-T cells were expanded for 14 days (supplemented with IL-2 every 2-3 days) and transduction efficiency was measured by flow cytometry using an anti-CD19 CAR antibody. Cells were cryopreserved prior to downstream functional assays.

Human and murine primary FRC isolation from fresh biopsy tissue and culture. FRCs were isolated as previously described (9) from human rLN tissues ('FRC') and patient DLBCL-LN tissues ('DLBCL-FRCS(p)'), and murine LN (brachial, axillary and inguinal) and spleen tissues from age-matched WT mice ('WT-FRCs') or matched DLBCL-diseased tissues from IμHA*Bcl6* mice (IμHA*Bcl6*-FRCs). Human and murine FRCs purified from fresh tissues were used at passage 1 in all comparative culture assays (DLBCL/ IμHA*Bcl6*-FRCs versus rLN/WT-FRCs). Briefly tissues were cut into small pieces and sequentially digested in a water bath at 37°C using a freshly prepared RPMI-based enzymatic mix containing Dispase, Collagenase P and DNase I (Sigma-Aldrich) (human: 2.4 mg/mL Dispase, 0.6 mg/mL Collagenase P and 0.3 mg/mL DNase I; mouse: 0.8 mg/mL Dispase, 0.2 mg/mL Collagenase P and 0.1 mg/mL DNase I). The

procedure was carried out for not longer than 1 hour. After complete digestion, cells (containing a mixture of fibroblasts and lymphocytes) were filtered through a 100μm nylon mesh, counted using trypan blue and plated at the concentration of $5x10^5$ cells/cm^2 in Fibroblast media (Sciencell). Non-adherent cells were removed after 48 hours, and media was subsequently changed every 2 days. After reaching confluency (3 to 5 days), adherent cells were washed with phosphate-buffered saline (PBS), detached using trypsin and their phenotype was assessed using flow cytometry. FRCs (passage 1) were either plated on fibronectin (Sciencell, 10μg/mL)-coated surfaces at a density of 10^4 cells/cm^2 for 2D cultures or seeded in a 48 well glass bottom culture plates (MatTek, 10^4 cells/well) in 100μl of 3D gel mix (collagen I: 3.2 mg/mL (Sigma-Aldrich, Merk), matrigel: 1,8 mg/mL (Sigma-Aldrich, Merk) for 3D assays. Aim V media was used in all the assays.

FRC conditioning. FRC conditioning with DLBCL B cells was by direct contact co-culture, unless otherwise stated, in Aim V media. Primary FRCs (Sciencell, passage 3) were conditioned with DLBCL cell lines for 5 days (cell lines on FRCs were split 1:2 at day 3) or with primary patient DLBCL B cells for 3 days (human: DLBCL-FRCs(c), murine: IμHA*Bcl6*-FRCs(c)) unless otherwise stated. FRCs conditioned with non-malignant B cell lines or primary B cells from rLNs (B cell-FRCs(c)) were prepared in the same way as DLBCL-FRC(c).

For 2D cultures, 10^4 FRCs or murine WT-FRCs were left to adhere on fibronectin (Sciencell, 10μg/mL)-coated plates overnight in Fibroblast Medium (24 well plate). DLBCL B cells resuspended in serum-free Aim V media (Gibco, Thermo Fisher Scientific) were added the following day (FRC:DLBCL ratio of 1:10 for DLBCL cell lines or 1:20 for primary human and murine DLBCL B cells). For direct contact co-cultures, DLBCL B cells were plated on top of FRC monolayers and after conditioning removed by gently washing FRCs with ice-cold PBS (Sigma-Aldrich). FRCs (unconditioned) were cultured alone at matched time-points as controls.

For transwell co-cultures, DLBCL B cells were plated in the upper chamber of a transwell plate (Corning, Merck) with a 0.4 μm pore size.

For 3D co-cultures, 10^4 FRCs and DLBCL B cells (FRC:DLBCL ratio 1:10 for cell lines) were co-seeded in a 48 well glass bottom culture plates (MatTek) in 100μL of 3D gel mix (collagen I: 3.2 mg/mL (Sigma-Aldrich, Merk), matrigel: 1,8 mg/mL (Sigma-Aldrich, Merk), with Aim V media, and incubated for the indicated time points. For contraction assays and T cell motility assays on FRCs in 3D, DLBCL-FRC(c) were first prepared in 2D culture before removal of the suspension DLBCL B cells to allow the harvesting and seeding of FRCs into 3D gels.

Recombinant TNFα (50 ng/mL, Peprotech), LTα_3 (10 ng/mL, R&D Systems), and LT$\alpha_1\beta_2$ (100 ng/mL, R&D Systems) were also used for FRC conditioning. For LTβR blockade, FRCs were pre-incubated with a LTβR blocking antibody (50μg/mL, R&D Systems) or isotype control for 1 hour at 37 °C prior to the addition of DLBCL B cells. Neutralizing antibodies against LTα_3 (10μg/mL, R&D Systems) and TNFα (10μg/mL, R&D Systems) or isotype controls were added directly to FRC:DLBCL co-cultures. Blocking and neutralizing antibodies were re-added every 24 hours for the duration of the assays (3 days).

Tumor survival assays with FRCs. For 2D survival assays, 10^5 DLBCL cells (cell lines) were seeded in serum starved conditions/media (RPMI, Thermo Fischer Scientific) and either cultured alone or co-cultured with FRCs (Sciencell) in a 48 well plate (ratio FRC:DLBCL: 1:10) (in triplicate per condition). At the indicated time points, tumor cell viability was assessed using Annexin V (Biolegend) and ToPro3 viability dye (Thermo Fisher Scientific) staining with flow cytometric analysis (BD Accuri C6 Plus, BD Biosciences). For 3D survival assays, 10^5 primary DLBCL B cells were seeded in 100μL of 3D gel mix alone or with $5x10^3$ FRCs (ratio 20:1) in serum starved conditions (RPMI, Thermo Fischer Scientific) (in triplicate per condition). At the indicated time points, gels were processed for confocal microscopy and image analysis. A neutralizing antibody against BAFF (10μg/mL, R&D Systems) or the corresponding isotype control was added to cultures where indicated and re-added every 24 hours until the end of the assay (5 days).

Ovalbumin uptake and processing. For antigen uptake, $5x10^4$ FRCs were seeded overnight in a 24 well plate and pulsed with Ovalbumin Alexa Fluor™ 488 Conjugate (50 µg/mL, ThermoFisher) for 15 minutes at 37 °C. For antigen processing, FRCs were pulsed with DQ-OVA (100 µg/mL, ThermoFisher) for 15

minutes and then incubated at 37 °C for 2 hours. After the indicated incubation times, FRCs were washed and analyzed by flow cytometry using the BD Accuri C6 Plus (BD Biosciences).

For complete antigen processing and presentation, FRCs were incubated for 24 hours with Ovalbumin (100 µg/mL, Sigma-Aldrich), washed, detached, and stained using a specific MHC I-SIINFEKL antibody. FRCs loaded with SIINFEKL (5 nM, Sigma-Aldrich) overnight were used as positive control. Samples were acquired using the BD Accuri C6 Plus (BD Biosciences).

Organotypic cultures.

Fresh excess human (**Supplemental Table 6**) or murine LNs were embedded in 4% low-melting agarose (Sigma-Aldrich) gel and allowed to solidify on ice for 15 minutes. Gels were then sliced at a thickness of 200 µm using a Vibratome (Leica VT1200) set to speed 1.5 mm/s and frequency 3 mm. Organ slices were immediately transferred in Aim V (Gibco, ThermoFisher Scientific) media and rested on organotypic culture inserts (Millicell, Millipore), stabilized using sterile stainless steel washers (10) and treated with the indicated drugs or control antibodies for up to 48 hours before immunofluorescence.

Contraction assay.

Following 2D FRC conditioning (DLBCL-FRCs(c)), FRCs were detached using trypsin, washed, and resuspended in Aim V media. 10^5 FRCs were added to 500µl of Collagen I (3.2mg/mL) Matrigel (1,8mg/mL) mix and seeded in a 24 well plate in triplicate per each condition. Gels were let to polymerize for 30 minutes at 37°C, detached from the borders of the well and covered with 500 µL AIM V media. Gel contraction was quantified at day 3 and measured as the ratio between the area of contracted gel/initial area, normalized to the control (FRCs alone).

G-LISA.

RhoA and Rac-1 activity in FRCs was measured using the luminescence-based G-LISA® RhoA and Rac-1 activation assays (Cytoskeleton, Inc) according to the manufacturer’s instructions.

Immunofluorescence staining

Immunofluorescence of cultured FRCs. DLBCL-FRCs(c) (or B cell-FRCs(c)) were generated and cultured on fibronectin-coated (Sciencell, 10µg/mL) glass coverslips, washed with PBS, and fixed with 3% paraformaldehyde for 15 minutes at room temperature. Cells were then permeabilized with 0,1% Triton X100 (Sigma-Aldrich), 4% BSA (Sigma-Aldrich) in PBS for 20 minutes and blocked with blocking solution (0.1% BSA in PBS) for 15 minutes. Primary antibodies, resuspended in blocking solution, were incubated for 1 hour with cells, before addition of secondary antibodies for 1 hour. Where indicated, for DNA detection, cells were stained 5 minutes with 4′,6-diamidino-2-phenylindole (DAPI, 0.5 µg/mL, CellSignaling Technologies), washed and mounted using antifade medium (FluorSave™, Merck Millipore).

Lipid rafts staining. Lipid raft labelling was carried out on ice on unfixed FRCs/DLBCL-FRCs(c), cultured on fibronectin-coated (Sciencell, 10µg/mL) glass coverslips, according to manufacturer's instructions (Vybrant lipid raft labelling kit 555, Thermo Fisher Scientific). Cells were then fixed in 4% paraformaldehyde before staining for PDPN and CD44. Samples were acquired using an A1R confocal microscope (Nikon).

Immunofluorescence of tissue. All tissues were freshly cut at 5 µm thickness using either a microtome (human FFPE) or a cryotome (paraformaldehyde-fixed, OCT-embedded, or snap frozen murine tissues). Human FFPE slices were dewaxed with xylene (Sigma) and rehydrated with decreasing concentrations of ethanol. Heat-induced antigen retrieval (MenaPath Access Retrieval Unit) was performed in citrate buffer (Dako) for both human and OCT-embedded murine samples.

Snap frozen murine tissues were fixed in acetone for 10 minutes before proceeding to staining.

Slides were blocked with 5% normal donkey serum and incubated overnight with primary antibodies. Secondary antibodies were then added for 1 hour. Where indicated, for DNA detection, tissues were stained for 5 minutes with DAPI (0.5 µg/mL, Cell Signaling Technologies), washed, and mounted using antifade medium (FluorSave™, MerckMillipore).

RNA In situ hybridization (RNA ISH). RNA-ISH was performed on 5 µm thick FFPE tissue sections using the RNAscope™ Multiplex Fluorescent V2 kit (ACD, Biotechne), coupled with TSA® Plus Fluorescence system (PerkinElmer), according to the manufacturer's instructions and using standard pre-treatment conditions (15 minutes pre-treatment and 30 minutes protease incubation). ISH was coupled with antibody staining to identify the cell type positive for the specific probe. In detail, the following staining panels were used: (I) Hs-LTA-310461, Hs-LTB-310471, Hs-TNFA-310421, anti-CD20 EP459Y (Abcam); (II) Hs-CCL19-474361, anti-podoplanin D2-40 (DAKO, Agilent). Positive and negative control probes were used to confirm the quality of the staining according to manufacturer's instructions (ACD, Biotechne).

Immunofluorescence of 3D gels/organotypic cultures. 3D gels and organotypic cultures were gently washed with PBS and fixed with 3% paraformaldehyde for 1 hour at room temperature. Samples were then permeabilized with 0,1% Triton X100 (Sigma-Aldrich), 4% BSA (Sigma-Aldrich) in PBS 1 hour and blocked with blocking solution (0.1% BSA in PBS) for 30 minutes. Primary antibodies were resuspended in blocking solution and incubated with samples overnight at 4°C. Where required, secondary antibodies were added and incubated for 1 hour at room temperature. For DNA detection, samples were stained 15 minutes with DAPI (0.5 µg/mL, CellSignaling Technologies) and mounted using antifade medium (FluorSave™, MerckMillipore).

T lymphocytes functional assays

Chemotaxis. To generate conditioned media (CM) from FRCs, WT and IµHA*Bcl6* LNs were enzymatically digested, and single cell suspensions plated in Fibroblast Media (24 well imaging plates, concentration of 5x10^5 cells/cm^2). After 4 hours, when FRCs started to adhere to the plate, suspension cells were washed with PBS and removed, and cultures topped up with 1 mL of AIM V media. FRC-specific media was collected after 48 hours and stored at -20°C (passage 0 FRCs) (11). At the same time point, the expression of CCL21 and CXCL9 in adherent FRCs was evaluated using immunofluorescence (11). Chemotaxis assays were performed in 96-well plates using transwell inserts with 5 µm pore size membrane (Corning®). 150 µL of AIM V alone (negative control), AIM V containing 1µg/mL CCL21

(positive control) or previously prepared CM from FRC cultures was added to the bottom chamber and, after re-equilibration for 30 minutes at 37°C, 10^5 CD3$^+$ TILs (IμHA*Bcl6*) or T cells (WT) were added to the top chamber. Migration was evaluated after 4 hours incubation at 37°C by quantifying the number of migrated cells in the lower chamber with a BD Accuri C6 flow cytometer. Each condition was performed in triplicate. The chemotactic index was calculated as the ratio between the number of migrated cells in each experimental condition and the number of migrated cells in the positive control sample.

For CCR7 blockade, CD3$^+$ TILs were pre-incubated with a CCR7 blocking antibody (10 μg/mL, R&D Systems) or isotype control for 1 hour at 37°C. For chemokine neutralization, antibodies against CCL21 (10 μg/mL, R&D Systems), CCL19 (10 μg/mL, R&D Systems), CXCL9 (10 μg/mL, R&D Systems), CXCL10 (10 μg/mL, R&D Systems), or isotype controls were added to the culture supernatants during the re-equilibration phase.

Motility. 10^5 FRCs or DLBCL-FRCs(c) (or WT-FRCs or IμHA*Bcl6*-FRCs) were generated and cultured on fibronectin (Sciencell, 10μg/mL) in 24 well imaging plates (Ibidi). 10^5 healthy CD3$^+$ T cells or CD3$^+$ TILs (as indicated) were added to FRCs and let to settle for 10 minutes at 37°C. T cells were then stimulated with 1 μg/mL CCL21 (Peprotech) for 5 minutes and imaging was performed using Ti2 wide-field imaging system (Nikon) with a 20x (ELWD) objective, using the NIS-elements software (Nikon). Image frames were captured at 1 minute intervals for 1 hour. Data analysis was performed using ImageJ by combining the MTrackJ and the Chemotaxis tool (Ibidi) plugins, at least 10 cells/movie were quantified per patient sample. Movies were extracted using the NIS-elements software (Nikon).

T cell (TIL or CAR-T-cell) circularity (perimeter^2/4 π area, shape factor tool, NIS-elements software Nikon) was determined by manually identifying cells on a still image (frame 30 that was the 30 minute time-lapse video time-point). At least 10 cells were quantified per condition. Higher values represent increased circularity.

For ICAM-1 blockade, stromal cells were pre-treated with ICAM-1 blocking antibody (Biolegend) at the indicated concentration or with an isotype control for 1 hour at 37°C before adding CD3$^+$ T cells.

For 3D motility, 5x10^5 FRCs or DLBCL-FRCs(c) (pre-conditioned in 2D culture) were stained with the CellTrace CFDA (Thermo Fisher Scientific), resuspended 100 µL of Collagen I (3.2mg/mL) Matrigel (1,8mg/mL) gels and plated into 48 well plate MatTek dishes (MatTek corporation). The day after, 10^5 $CD3^+$ TILs were stained with CellTrace Violet (200nm, Thermo Fisher Scientific), resuspended in 80 µL of matrigel and plated on top of the FRCs gel. Gels were left to settle for 3 hours to allow T cells to move towards FRCs by gravity and topped up with 200 µL of Aim V media. After the addition of 1 µg/mL of CCL21 (Peprotech), gels were imaged for 4 hours using A1R confocal microscope (z-stack: 1 µm thickness, 2 minutes interval).

Movies were analyzed using Imaris v9.7.2 (Bitplane). Briefly, both the surface and the spots tools were used to segment images (blue channel, CellTrace Violet) and to identify T cells. The surface tool was then used to calculate T cells sphericity while the spots tool was used to track T cells over time. At least 15 cells/movie were quantified per patient sample.

Adhesion. 10^5 FRCs or DLBCL-FRCs(c) were generated and cultured on fibronectin-coated (Sciencell, 10µg/mL) glass coverslips placed in a 24 well plate. 10^5 healthy $CD3^+$ T cells (untreated or previously activated overnight with 1 µg/mL of soluble agonistic CD3 and CD28 antibodies) were stained with CFDA (Thermo Fisher Scientific) and added to stroma cultures. After 4 hours at 37°C, cultures were washed three times with PBS, cells fixed for 15 minutes at room temperature with 3% formaldehyde (Thermo Fisher Scientific), permeabilized with 0.3% Triton X-100 (Sigma-Aldrich) and stained with Rhodamine Phalloidin (Thermo Fisher Scientific) and DAPI (0.5 µg/mL, CellSignaling Technologies). Coverslips were then mounted on microscopy slides using antifade medium (FluorSave™, MerckMillipore). Coverslips coated with 1 µg/mL ICAM-1 (R&D Systems) were used as positive control. All assays were performed in triplicate.

Immune Synapse formation. Immune synapse assays and quantitative analysis were performed as previously described (12). Briefly, 10^6 human or murine $CD8^+$ TILs were stimulated with 1 µg/mL soluble agonistic CD3 and CD28 antibodies in presence of 10^5 FRCs (WT-FRCs) or DLBCL-FRCs(p) or IµHA*Bcl6*-FRCs (FRC-T cell ratio 1:10) in a 24 well plate. The day after, T cells were collected and mixed with 10^5

primary DLBCL cells previously stained with CellTracker Blue CMAC, according to the manufacturer's instructions (Thermo Fisher Scientific). DLBCL-T cell cultures were centrifuged at 260xg for 5 minutes and incubated at 37°C for 30 minutes to allow the formation of conjugates.

Cells were then transferred onto poly-L-lysine slides (Thermo Fisher Scientific) using a cell concentrator (Cytofuge 2), fixed for 15 minutes at room temperature with 3% formaldehyde (Thermo Scientific), permeabilized with 0.3% Triton X-100 (Sigma-Aldrich) for 5 minutes before proceeding with antibody staining. Primary and secondary antibodies were applied sequentially for 45 minutes at 4°C in 5% goat serum (Sigma-Aldrich) blocking solution. To visualize the synapse area, F-actin was always stained using Rhodamine Phalloidin (Thermo Fisher Scientific) following manufacturer's instructions. After washing, slides were mounted using antifade medium (FluorSave™, Merck Millipore) and analyzed using confocal microscopy.

Cytotoxicity. 10^4 human/murine $CD8^+$ TILs, resuspended in Aim V media, were stimulated for 24 hours with 1 µg/mL soluble agonistic CD3 and CD28 antibodies (Biolegend) alone or in presence of 10^3 FRCs (or WT-FRCs) or DLBCL-FRCs (DLBCL-FRCs(p) or IµHA*Bcl6*-FRCs) (FRC-T cell ratio 1:10) in a 96-well plate. The day after, 10^3 DLBCL B cells (tumor targets) were stained with CellTrace CFDA (200nM, Thermo Fisher) and added to the CD8-FRCs co-cultures (FRC-CD8-tumor B cell ratio 1:10:1). After 4 hours, cells were recovered, washed, and stained with Annexin V (Biolegend) and ToPro3 viability dye (Thermo Fisher), according to manufacturer's instructions. T cell-mediated cytotoxicity against tumor cells was determined by flow cytometry using the BD Accuri C6 Plus (BD Biosciences). Cytotoxicity was calculated as: % target cell death = 100- ((% $CFDA^+$ $ToPro3^-$ $AnnexinV^-$ target cells incubated with effector T cells) × 100 / (% $CFDA^+$ $ToPro3^-$ $AnnexinV^-$ target cells alone)). All cytotoxicity assays were performed in triplicate.

For antigen-specific cytotoxic assays, WT-FRCs or IµHA*Bcl6*-FRCs were cultured in Aim V media and loaded with 5 nM SIINFEKL peptide overnight (Sigma-Aldrich). After washing, $CD8^+$ OT-I cells were added to the culture (stroma-T cell ratio 1:10) and stimulated with 1 µg/mL soluble anti-CD3 and anti-

CD28 for 24 hours. Cytotoxicity was performed using target DLBCL B cells loaded with 5nM SIINFEKL peptide for 1 hour.

For PD-L1/PD-L2 (Biolegend) blockade, WT-FRCs or IμHA*Bcl6*-FRCs were pre-incubated with 1 μg/mL blocking antibodies for 1 hour at 37 °C. After washing, $CD8^+$ T cells were added to the culture and the assay performed as described above.

To measure cytotoxicity associated with T cell-engaging bispecific antibody and FRC-targeted drug treatment, FRCs or DLBCL-FRCs(c) were generated in a 96 well plate before being co-cultured with $CD8^+$ TILs together with target autologous DLBCL B cells (FRCs-CD8-tumor ratio 1:10:1) for 48 hours in presence of control antibodies (vehicle) or bispecific drugs alone or in combination, as indicated.

T lymphocytes-FRCs interaction. 10^5 FRCs or DLBCL-FRCs(c) were generated and cultured on fibronectin-coated (Sciencell, 10μg/mL) glass coverslips placed in a 24 well plate. 10^5 healthy $CD3^+$ T cells (previously activated overnight with 1 μg/mL of soluble agonistic CD3 and CD28 antibodies), resuspended in AIM V media, were added to the culture. After 4 hours at 37°C, cultures were washed, fixed, permeabilized and stained for DAPI, actin and PD-1. After washing, slides were mounted using antifade medium (FluorSave™, MerckMillipore) and analyzed using confocal microscopy.

Immunofluorescence and confocal imaging.

Background of all fluorescence signals was verified by staining samples with the appropriate secondary antibodies alone and with unstained sample controls. Whole tissue acquisition was performed using VS120 fluorescence scanner (Olympus) with a 20x/0.75 air objective. Each staining panel was imaged in batch to minimize variability across samples using the same fluorescence exposure time. Images were then adjusted for brightness and contrast and exported using the Olyvia software (Olympus).

For confocal microscopy, images were captured using a high-sensitivity A1R confocal microscope with NIS-elements imaging software (Nikon). Medial optical section (or Z-stacks for 3D volume images) of cell cultures and tissues were imaged using a 20x Plan APO lens NA 0.75, organotypic cultures were imaged with a 40x Plan Fluor 40x NA 1.3, immune synapses were acquired using a 60x N Apo NA 1.4 λs

oil objective. Detectors were set to detect an optimal signal below saturation limits and staining panels were acquired during the same session using identical acquisition settings.

Image analysis

FRC network. FRC network analysis (Gap and skeleton) was performed on the PDPN signal (or ER-TR7 for murine tissues), as previously described (13, 14). Briefly, images were imported in ImageJ, cropped to exclude germinal centers/follicles or PDPN negative areas (e.g. red pulp for splenic tissues), thresholded and converted into binary images. Gap analysis was performed using a specific Matlab script (13), while skeleton analysis was carried out with ImageJ. At least 3 Field Of View (FOVs) were analyzed per sample.

The area occupied by the FRC network was measured using NIS-Elements software (Nikon), after PDPN or ER-TR7 thresholding, and expressed as a percentage of the total tissue area for each FOV (20X, 1024x1024, 0.61µm/px, 0.39mm^2) (3 FOVs analyzed/sample).

The percentage of FRCs expressing a specific marker (FAP, BAFF, CCL21, CXCL9, PD-L1, PD-L2) was determined with image thresholding and calculated relative to the total PDPN$^+$ or ER-TR7$^+$ FRC network area in each FOV using NIS-elements (3 FOVs analyzed/sample).

FRC cultures. Cell shape of cultured FRCs was calculated using cell shape index analysis (circularity = perimeter^2/4πarea); the area and perimeter of each single FRC (manually identified with F-actin staining using rhodamine phalloidin) was obtained using NIS-elements software. At least 20 FRCs/condition were quantified. FRC length in 3D cultures was determined by manually measuring the major axis of each cell using NIS-elements software. At least 10 FRCs/gel were quantified.

For lipid raft-PDPN and PDPD-CD44 analysis, the colocalization coefficient R was measured using pixel intensity with NIS-elements. At least 10 cells were quantified for each condition.

For 3D tumor survival assays with FRCs, a full stack of the gel was imaged and the number of tumor cells in each FOV was manually counted (2 FOVs analyzed/sample).

For CCL21 and CXCL9 expression by passage 0 FRCs, fibroblasts were identified using actin staining and chemokine Mean Fluorescence Intensity (MFI) was calculated using NIS-elements. At least 10 cells were quantified for each condition.

Immune synapses. T cell-DLBCL B cell immune synapse and T cell-FRC contact site interaction analysis was performed by drawing a region of interest (ROI) around each cell-cell conjugation contact site using NIS-elements (Nikon). F-actin (rhodamine phalloidin staining) synapse (T cell-DLBCL) or contact (T cell-FRC) areas (μm^2), Granzyme B (GrB) and PD-1 MFI were calculated. At least 20 interactions were scored for each condition.

T cell and CAR-T analysis in tissues. CAR-T cell identification and circularity in DLBCL-LN biopsy tissues was detected by thresholding and combining the fluorescence signal (co-localizaton) of anti-CD8 and the anti-Human IgG (H+L) antibody signals using the surface tool in Imaris v9.7.2 (Bitplane). $CD8^+$ T cell counts in murine and human tissues, and CAR-T cell counts in biopsy tissues were performed using the spots detection algorithm in Imaris v9.7.2 (Bitplane). The number or CAR-T cells in contact with FRCs was calculated using the "spots close to surface" (threshold set at 10µm distance between CAR T-FRCs) XTension from Imaris v9.7.2 (Bitplane).

Organotypic cultures. 3D organotypic culture image reconstruction and analysis were performed using surfaces or volume rendering tools from Imaris v9.7.2 (Bitplane). To measure tumor cell viability before and after drug treatment, images were pre-processed using the median filter tool. The surface tool was then used to threshold fluorescence signal and to calculate the volume occupied by tumor B cells ($CD20^+$ or $B220^+$ signals for human and mouse samples respectively) and by the double positive $CD20^+$/$B220^+$ and cleaved caspase-3^+ tumor cells. Tumor cell viability ($cCasp3^+$ tumor B cells) was then calculated as the ratio between the volume occupied by tumor B cells also positive for cleaved caspase-3 and the volume occupied by the total tumor B cell population.

Flow cytometry

FRC staining. FRCs were detached with Trypsin (Sigma), washed, and counted. 10^5 cells were resuspended in 500 μL of PBS and incubated with the Fixable Viability Dye eFluor™ 780 (Thermo Scientific) for 30 minute at 4 °C. After washing, cells were resuspended in 100μL of PBS containing 2% FBS and incubated with fluorochrome-conjugated antibodies against cell surface markers at 4 °C for 30 min. For cytokine detection, adherent cells were stimulated at 37°C in AIM V with Golgi plug (BD Biosciences) for 4 hours prior to intracellular staining performed using the BD Cytofix/Cytoperm Kit (BD Biosciences). To exclude the presence of any residual tumor cells when analyzing DLBCL-FRCs(c), an anti-CD20 antibody was added to panels.

T cell staining. 10^6 splenocytes (murine samples) or LN-derived single cell suspensions (human samples), derived from mechanically dissociated organs, were resuspended in 500 μL of PBS and incubated with Fixable Viability Dye eFluor™ 780 (Thermo Scientific) for 30 minutes at 4 °C. After washing, cells were resuspended in 100 μL of phosphate-buffered saline (PBS) containing 2% FBS and incubated with fluorochrome-conjugated antibodies against cell surface markers at 4 °C for 30 min. Transcription factor staining (TCF-1) was performed using the Transcription Factor Staining Buffer Set (BD Biosciences).

Data acquisition and analysis. BD FACSCanto II and Fortessa cell analyzer (BD Biosciences) were used for samples acquisition. Data analysis was performed with FlowJo v.10. Live cells were gated using the viability dye negative stained cell populations. Control isotype antibodies were used to correct fluorescence signals for non-specific background staining. Compensation was performed using anti-mouse Ig, κ/negative control compensation particles set (BD Biosciences) for lymphocytes and the anti-mouse Ig, κ/negative control (BSA) compensation plus (7.5 μm) particles set (BD Biosciences) for FRCs.

RT-PCR.

FRCs or DLBCL-FRCs(c) were cultured for the time-points specified, before DLBCL cells were removed and total RNA was extracted from FRCs using the RNeasy isolation kit (Qiagen). Quantitative RT-PCR

analysis of FRCs was performed using TaqMan assays (Pdpn: Hs00366766_m1, Fap Hs00990806_m1, Gapdh Hs02786624_g1) and the StepOnePlus™ Real-Time PCR System (Thermo Fisher Scientific).

Luminex.

FRCs or DLBCL-FRCs(c) were co-cultured with CD8$^+$ TILs and autologous target tumor B cells (stroma-CD8-tumor cell ratio 1:10:1) in presence of T cell-engaging bispecific antibody as indicated. Co-culture supernatants were collected after 48 hours, centrifuged, and analyzed using a custom designed Human Luminex assay kit (RnD Systems). Samples were acquired with the Luminex FLEXMAP 3D system (RnD Systems) according to manufacturer's instructions.

Imaging Mass Cytometry (IMC)

Staining. 5 µm thick FFPE rLN control tissues (n=3) and the DLBCL TMA (containing 53 patients, two core biopsy areas per patient) were dewaxed with xylene (Sigma) and rehydrated with decreasing concentrations of ethanol. Heat-induced antigen retrieval was performed in Antigen Retrieval Reagent (R&D Systems) in a 96 °C waterbath for 30 minutes. Tissues were then blocked with 3% BSA and incubated overnight with the metal-tagged antibodies listed in **Supplemental Table 2**. The day after slides were washed, incubated for 5 minutes with Cell-ID™ Intercalator (Standard BioTools) to stain cell nuclei, washed and left to dry before proceeding with IMC acquisition.

Acquisition. Images were acquired using the Hyperion Imaging System (Standard BioTools). IMC-stained slides were loaded on the Hyperion initially to obtain high resolution (20X) light-microscope composite images (panoramas) of approximately 4 mm^2. From each panorama, a 1 mm^2 region of interest area (ROI, corresponding to a complete core of the TMA) was selected for laser ablation at 1 mm^2/pixel resolution and at 200 Hz frequency. Our IMC dataset has been deposited in: 10.5281/zenodo.6491226 (**Supplemental Table 7**).

Analysis. Raw output files (.mcd or text) were converted into 32bit ome.tiff stacks using MCD^{TM} Viewer (Standard BioTools) and corrected by applying a spillover matrix (maximum threshold 98th percentile of the original raw data). Germinal centers, identified in 4/100 analyzed DLBCL cores and in 1/3 rLN tissue areas, were excluded from all the analysis.

Stroma analysis. After hot pixel removal (https://github.com/BodenmillerGroup/ImcPluginsCP), Ilastik was used to analyze the single-channel images for Vimentin (Vim), PDPN, FAP, CD31 and CD68 for pixel classification and probability map generation(15). The three main LN stromal subsets (LECs, BECs, FRCs) were identified by combining (excluding, adding and/or multiplying) the different probability maps in CellProfiler to create subset-specific stromal masks (**Supplemental Figure 1A**, FRCs: PDPN$^+$, Vim$^+$, FAP$^+$, CD68$^-$, CD31$^-$; LECs: PDPN$^+$, CD31$^+$, Vim$^-$, FAP$^-$, CD68$^-$, BECs: PDPN$^-$, CD31$^+$ Vim$^-$, FAP$^-$, CD68$^-$). The generated stromal masks were then used to measure the total area occupied by each subset (expressed as a percentage of the total ROI area) and the expression levels (integrated, mean and median pixel intensity) of PDPN, FAP, PD-L1 and PD-L2 on the FRC network.

For the morphological classification of the FRC network, FRC masks were segmented in CellProfiler. Primary objects were identified using a typical diameter of 10-250-pixel units with a smoothing filter size for declumping of 80 and a minimum allowed distance between local maxima of 20. The identified FRC objects were classified in 4 categories according to their circularity (values>0.32: circular, values<0.32: elongated) and complexity (measured as the ratio between the maximum Feret diameter and minimum Feret Diameter, ratio>1.93: simple, ratio<1.93: complex): (1) complex circular (circularity$^+$/complexity$^+$), (2) complex elongated (circularity$^-$/complexity$^+$), (3) simple circular (circularity$^+$/complexity$^-$); (4) simple elongated (circularity$^-$/complexity$^-$) (**Supplemental Figure 1H**).

FRC checkpoint clusters (PD-L1$^-$/PD-L2$^-$, PD-L1$^+$/PD-L2$^-$, PD-L1$^-$/PD-L2$^+$, PD-L1$^+$/PD-L2$^+$) were defined combining the previously generated FRC masks with the PD-L1/PD-L2 masks and measuring the area occupied by each cluster with CellProfiler (module "MeasureImageAreaOccupied").

CD8$^+$ TIL characterization and clustering. CD8$^+$ T lymphocytes were identified and counted using CellProfiler by multiplying the probability maps for CD8 and CD3 generated in Ilastik. We also extracted

single cell data measurements such as cell shape (circularity = perimeter^2/4πarea) and marker expression (integrated, mean, and median intensity expression for LAG3, PD-L1, PD-L2, TIM3, PD-1 and Granzyme B) in CellProfiler.

The identified CD8$^+$ objects were then masked with the previously generated FRC network mask to analyze CD8-FRC interactions. A minimum overlap of 10% between the identified CD8 and the FRC network mask was selected to exclude CD8 cells in proximity but not in contact with FRCs.

The integrated intensity of LAG3, PD-L1, PD-L2, TIM3, PD-1 and Granzyme B (GrB) was used for the clustering of CD3$^+$, CD8$^+$ T lymphocytes into groups of phenotypically similar cells (PhenoGraph as implemented in the Cyofkit R package, available at https://github.com/JinmiaoChenLab/cytofkit (16)).

The k-neighbors value was selected according to the square root of the number of total events and adjusted to optimize the clustering (K = 211). Of the resulting 13 clusters, 4 were aggregated into a larger group following the hierarchical clustering (Euclidean distance) of their median integrated marker intensities. For visualization, high-dimensional single cell data were reduced to 2 dimensions using the *t*-distributed stochastic neighbor embedding (t-SNE) algorithm and each cluster represented by a different color on the map using FlowJo software (BD Bioscience, version 10.7.1).

The frequency of CD8$^+$ cells in each cluster for each sample was generated using a custom MATLAB script and used to identify differences in the abundance of each cluster across all the patient samples.

TFE identification. Unsupervised hierarchical clustering of the normalized frequency (Z-score) of each identified CD8$^+$ TIL and FRC cluster was used to define the DLBCL-TFEs using a custom script in Matlab (version R2020b). Spearman's rank correlation was used as statistical method to verify the correlation between the identified clusters and specific subsets of patients.

Spatial analysis. New FCS files were then created on MATLAB incorporating the cluster ID for each CD8$^+$ cell and used for the neighborhood analysis. The distance between each CD8$^+$ cell and the FRC network was defined as the median Euclidian distance between the center of each CD8 cell and the closest 15 FRC pixels. CD8$^+$ and FRCs were considered as ‘interacting’ when the median distance was below 10µm.

Sorting/FACs

Enzymatically digested murine tissues (LN (brachial, axillary, and inguinal) and spleen tissues from age-matched WT mice or matching DLBCL-diseased tissues from IµHA*Bcl6* mice) were resuspended in PBS containing 2% FBS and B cells were depleted using a $CD19^+$ positive selection kit (Stemcell). CD19-depleted samples were stained for CD45, PDPN and CD31 for 30 minutes at 4°C. Samples were then washed, and stromal cells sorted with a FACSAria II (BD Biosciences). The gating strategy for low-input bulk RNASeq is shown in **Supplemental Figure 5H** and **J**. For scRNA-seq, after LN enzymatic digestion and antibody staining, $CD45^-$ stromal cells ($PDPN^+$ and/or $CD31^+$) were FACS purified and further processed for scRNASeq. In all the procedures, dead cells were excluded using DAPI staining.

Bulk RNASeq

Bulk human RNA-Seq. FRCs (Sciencell) at passage 3 were cultured alone (n=3) or conditioned with either DLBCL cell lines (n=8, SU-DHL4, SU-DHL10, SU-DHL16, RL, WSH-NHL, TMD8, OCI-Ly10, OCI-Ly18), B cell lines (n=3, JY, 111BLCL, 00136), primary DLBCL B cells (n=4 patients) or primary rLN-derived B cells (n=3) for 48 hours using transwell co-culture to avoid any risk of tumor cell contamination (**Supplemental Table 3**). DLBCL-FRCs(p) were isolated from patient DLBCL-LNs (n=2) and used for sequencing at passage 1. Total RNA was extracted using the RNeasy isolation kit (Qiagen). Library preparation of extracted mRNA was performed utilizing the TruSeq stranded mRNA kit (Illumina) and the resulting library sequencing on an Illumina HiSeq 2500 system (Illumina). The RNA-seq data derived from this study have been deposited in the Gene Expression Omnibus (GEO) repository (GSE179161).

Bulk murine low input RNA-Seq. $CD45^-$ $CD31^-$ $PDPN^+$ FRCs were sorted into lysis buffer from the Agencourt RNAdvance Cell v2 kit (Beckman Coulter) and RNA extracted using SPRI magnetic bead-based extraction (Beckman Coulter). Ultra-low input Non-stranded, PolyA enriched SMARTSeq V4 + NuGEN library preparation was carried out followed by MiSeq QC and HiSeq2500 Rapid 2X100 cycles. The RNA-seq data derived from this study have been deposited in the Gene Expression Omnibus (GEO) repository (GSE179193).

Analysis. Fastq quality was evaluated using FastqQC (v 0.11.5). The fastq files were tested using bbmap reformat (v.37.24) for compliance with the fastq format and to convert the quality encoding to q33 if necessary. In all analyses the primary assembly of hg38 was used (ENCODE version) and the annotation from Gencode v24. Alignment was performed using a two pass mode with STAR (v2.5.2b(17)) on the full genome and counts were obtained using the quantmode GeneCounts option. STAR was also used to trim the reads on the fly using the clip3pAdapterSequence option. Differential expression was performed using DESeq2 (v. 1.26.0).

Pathway Analysis. Gene lists were processed for GSEA (v. 3.0(18)) by taking the inverse of the FDR adjusted P value for each gene and multiplying it by the sign of the log2 fold change relative to the FRC Control condition. GSEA was run under the "prerank" mode with 1000 permutations for each of the gene sets available in MSigDB version 7.4.

scRNA-seq.

Freshly sorted LN stromal cells ($CD45^-/CD31^{+/-}/PDPN^{+/-}$) from two IμHA*Bcl6* and one WT age matched mice were counted using a hemocytometer and encapsulated into emulsion droplets using the Chromium Controller instrument (10× Genomics), where cell lysis and barcoded reverse transcription of RNA were performed. The scRNA-seq libraries were constructed using the Chromium Single Cell 3′ Library, Gel Bead & Multiplex Kit (10× Genomics, V2 and V3) according to the manufacturer's instructions.

Single-cell 3′ RNA-seq libraries were generated according to the manufacturer's instructions (Chromium Single Cell 3′ Reagent v3 Chemistry Kit, 10X Genomics, Inc.). Libraries were sequenced to an average depth of ~20,000 reads per cell on an Illumina HiSeq 2500 system.

Sequenced reads from all libraries were aligned and quantified using the Cell Ranger 3.1.0 (10X Genomics, Inc.) against the mouse reference genome mm10-3.0.0 and the Cell Ranger Aggr function was used to combine biological replicates into a single dataset. The Seurat pipeline version 3.1.4 (19). Cells were subsequently filtered based on unique features (nFeature_RNA > 200 genes and <5000),

total number of molecules detected within a cell (nCount_RNA) > 200 and mithocondrial counts < 5%. Libraries were anchored and integrated using the top 2000 variable features per library calculated via the "vst" method in Seurat. Canonical correlation analysis (CCA) on these 2000 features between the libraries was calculated, and the first 20 dimensions used as input for anchoring. Post anchoring, PCA was performed and the first 10 PC's were used for UMAP dimensionality reduction and subsequent clustering using the default Louvain implementation.

The above procedure was performed on the entire dataset and repeated on the *Pdpn*$^+$, *Pdgfra*$^+$ FRC population identified within all IµHA*Bcl6* and WT samples.

Differential expression tests to identify cluster markers and genes significantly deregulated between WT and IµHA*Bcl6* samples were carried out with the Seurat package, by selecting the Wilcoxon Rank Sum test procedure. Genes were called differentially expressed when the absolute value of the log2 fold change was higher than or equal to 0.25 and the adjusted p-value less than 0.001. Similarity between the transcriptional profiles of the FRC clusters identified in this study and those previously reported was estimated with GSEA. Gene signatures of the cell populations identified by Rodda et al. (20) and Kapoor et al. (21) consisted of the cluster markers with an average log fold change greater than 0.5, an adjusted p-value less than 0.001 and a proportion of cluster cell expressing the gene higher than 0.1. Enrichment of these gene lists was calculated using GSEA after sorting the genes detected in this present study by decreasing log fold changes used to identify cluster markers. The sequencing data derived from this study have been deposited in the Gene Expression Omnibus (GEO) repository (GSE193565).

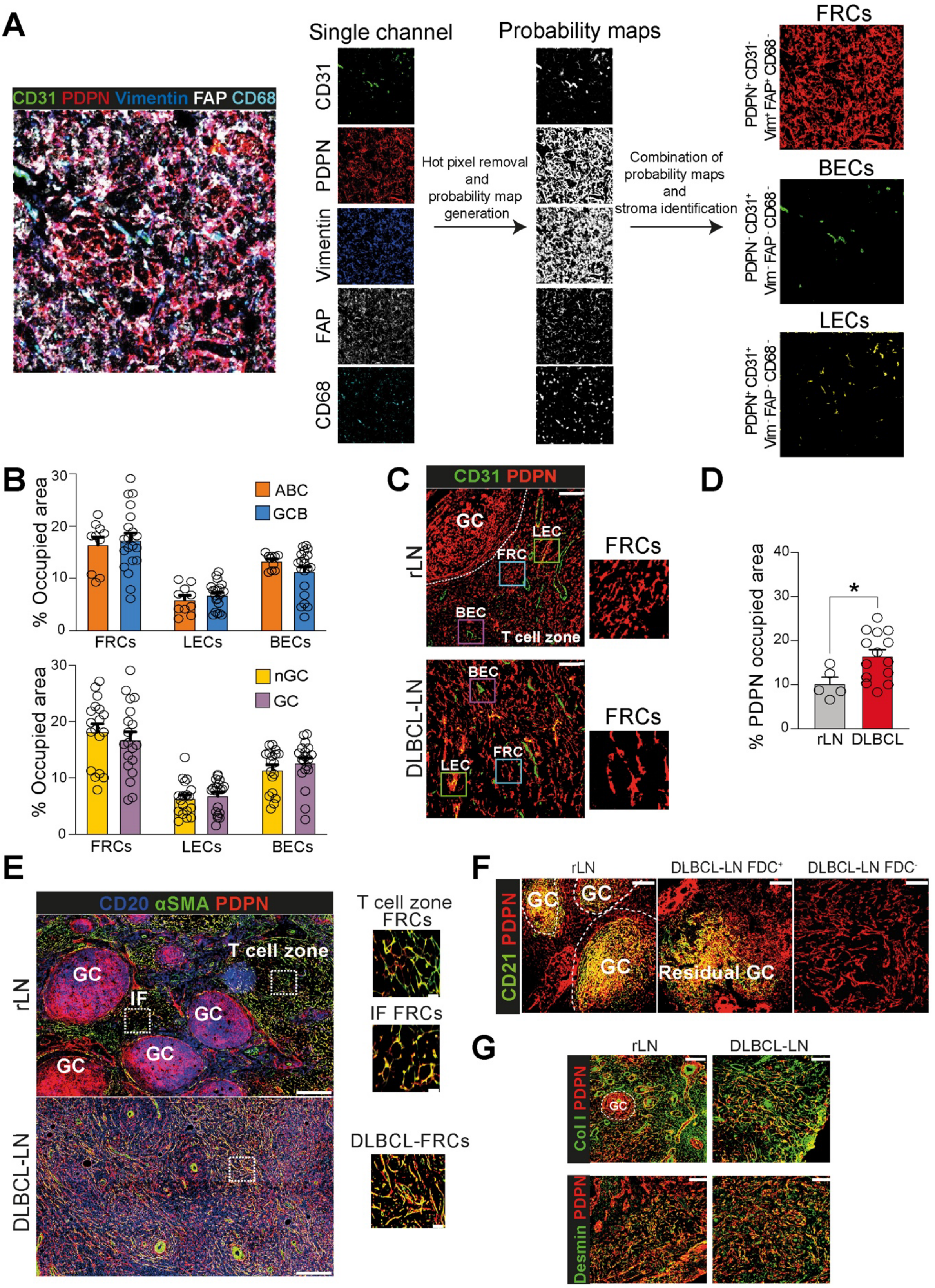
A
CD31 PDPN Vimentin FAP CD68
Single channel
CD31
PDPN
Vimentin
FAP
CD68
Hot pixel removal
and
probability map
generation
Probability maps
Combination of
probability maps
and
stroma identification
FRCs
BECs
LECs
B
% Occupied area
ABC
GCB
nGC
GC
FRCs
LECs
BECs
C
CD31 PDPN
rLN
GC
LEC
FRC
BEC
T cell zone
FRCs
DLBCL-LN
D
% PDPN occupied area
*
rLN
DLBCL
E
CD20 αSMA PDPN
T cell zone
IF
T cell zone FRCs
IF FRCs
DLBCL-FRCs
F
rLN
DLBCL-LN FDC+
DLBCL-LN FDC-
CD21 PDPN
Residual GC
G
Col I PDPN
Desmin PDPN

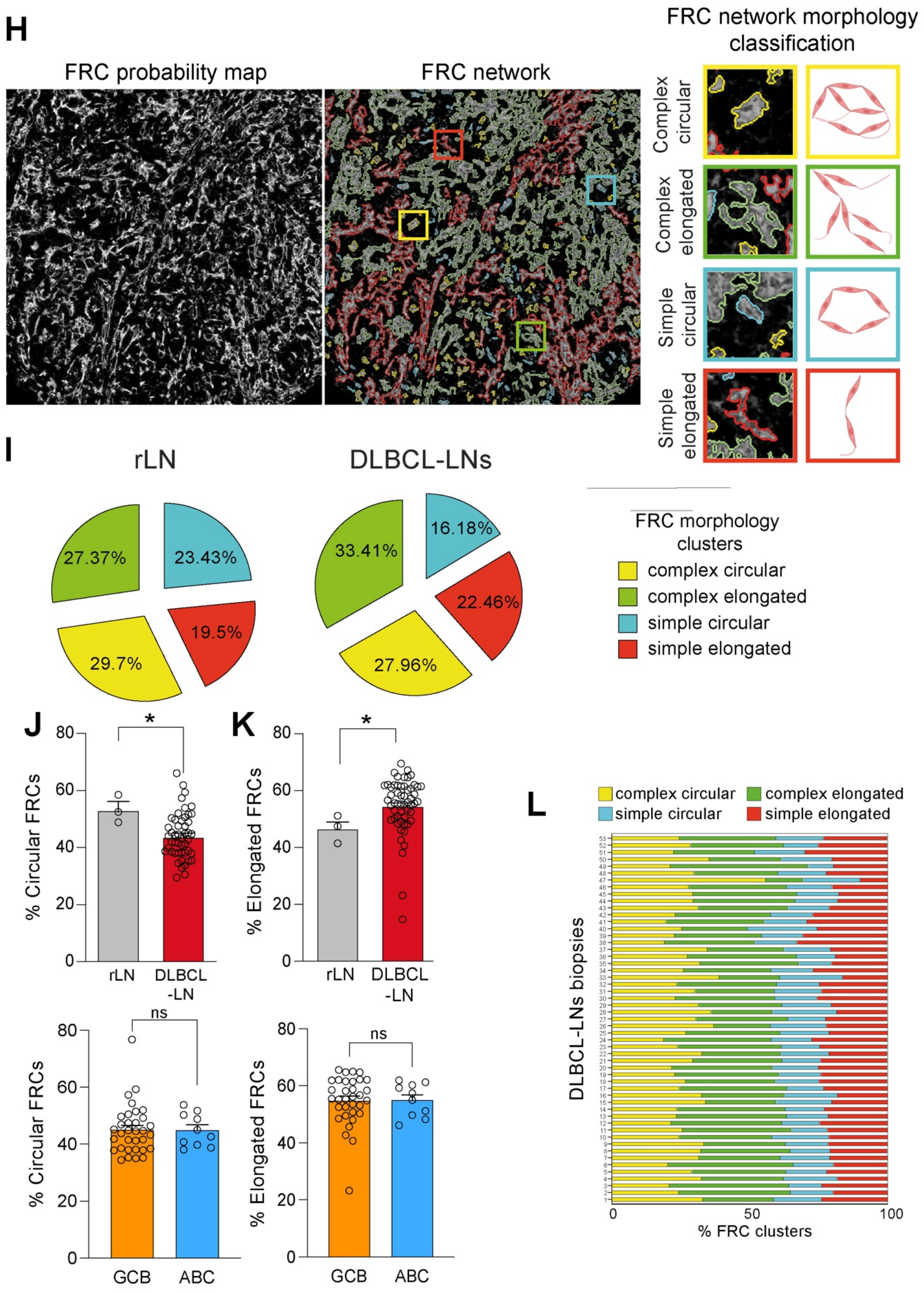

Supplemental Figure 1. FRC remodeling in human DLBCL-LN tissue biopsies. (**A**) IMC stroma pipeline schematic. Left panel: representative merged IMC image of a DLBCL-LN. Probability maps for each marker (CD31, PDPN, Vimentin, FAP and CD68) were used to generate binary masks associated to each stromal subset (FRCs: PDPN^{+}, CD31^{-}, Vim^{+}, FAP^{+}, CD68^{-}; LECs: PDPN^{+}, CD31^{+}, Vim^{-}, FAP^{-}, CD68^{-}; BECs: PDPN^{-}, CD31^{+}, Vim^{-}, FAP^{-}, CD68^{-}). (**B**) Area occupied by FRCs, LECs and BECs in 53 DLBCL-LNs. Patients were grouped according to the COO using nanostring (upper graph) or immunohistochemistry (lower

graph). (**C**) Representative confocal images showing FRCs, LECs and BECs identified using PDPN and CD31, in rLN (upper panel, n=10) and whole DLBCL-LNs (lower panel, n=10). Squares highlight FRCs (light blue), BECs (pink) and LECs (green). GC: germinal center. Scale bar, 100μm. (**D**) Area occupied of PDPN$^+$ FRCs in rLN (n=5) and whole DLBCL-LN tissues (n=14). (**E**) Representative immunofluorescence images showing CD20, PDPN and αSMA in rLN (n=5) and whole DLBCL-LNs (n=15). IF: interfollicular. Scale bar, 100μm. Magnified FRC network images indicated. Scale bar, 20μm. (**F**) Representative confocal images of FDCs (CD21$^+$ PDPN$^+$) and FRCs (CD21$^-$ PDPN$^+$) in rLN (n=5) and DLBCL biopsies (n=53, one representative DLBCL-FDC$^+$ and DLBCL-FDC$^-$ sample). Scale bar, 100μm. (**G**) Representative confocal images of rLN (n=5) and whole DLBCL-LNs (n=5) stained for PDPN and collagen I (upper panels) or desmin (lower panels). Scale bar, 100μm. (**H**) IMC-based analysis to identify FRC morphological clusters: complex circular (yellow), complex elongated (green), simple circular (light blue), simple elongated (red) (representative DLBCL-LN IMC data shown). Panels (right) show magnified and schematic representation of the 4 identified morphological cluster categories. Briefly, 'complex circular' included FRCs exhibiting an increased number of branch points and a contracted morphology; 'complex elongated' contained FRCs showing increased branch points and a more stretched morphology; 'simple circular' incorporated FRCs exhibiting fewer branch points and a contracted morphology; 'simple elongated' included FRCs showing less complex branch points and a more stretched morphology. (**I**) Proportion of morphological clusters within the FRC network of rLNs (n=3) and DLBCL-LNs (n=53). (**J**, **K**) Frequency of circular (**J**) and elongated (**K**) morphological clusters within the FRC network of rLN (n=3) and DLBCL-LNs (n=53) (upper graphs) and within GCB (orange) and ABC (blue) COO subtypes of DLBCL (lower graphs). (**l**) Frequency of morphological clusters within the FRC network of 53 DLBCL-LNs. Data in (**B**, **D**, **J**, **K**) are shown as mean ± SEM, *P < .05, Mann–Whitney U-test.

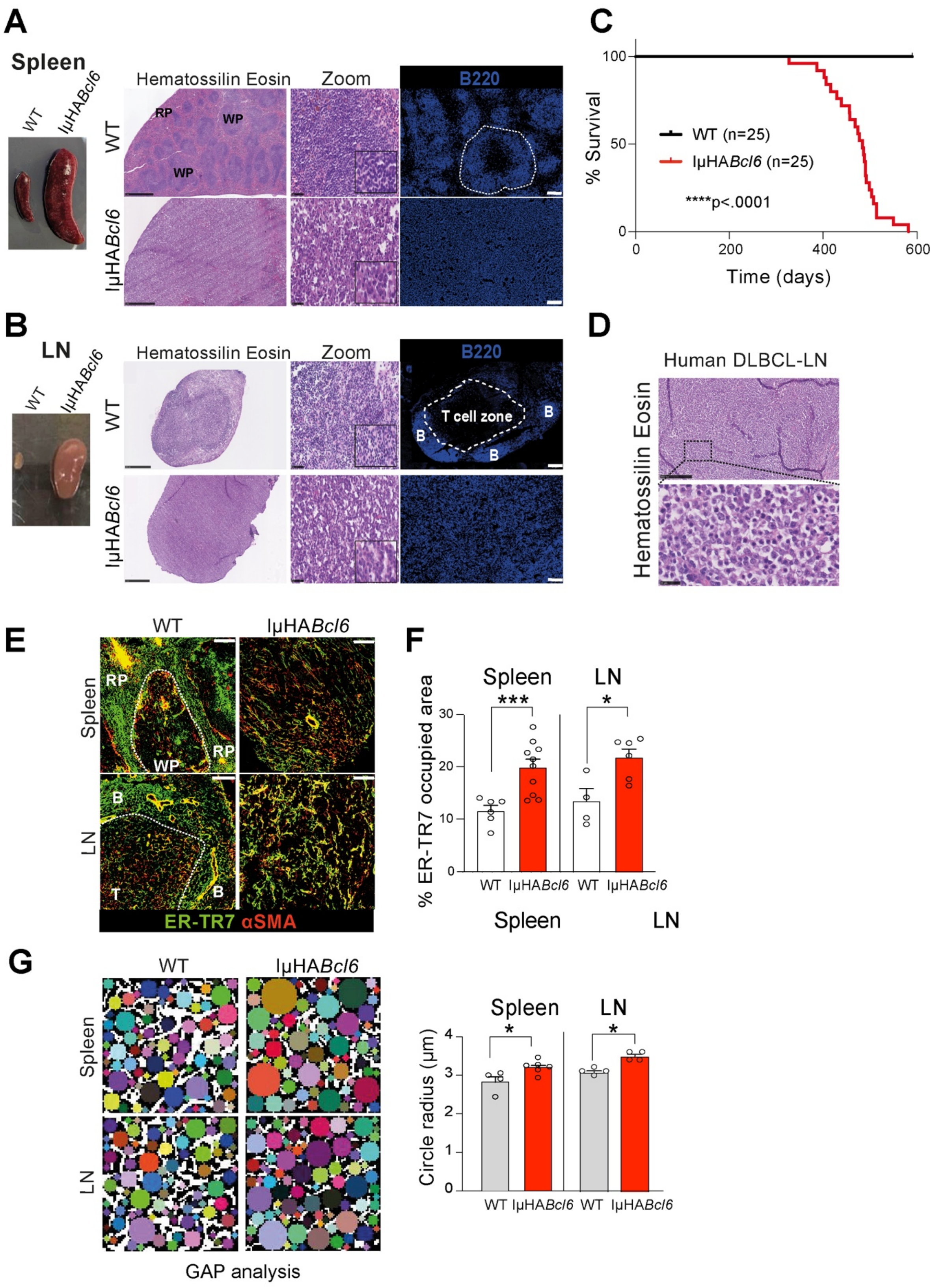
A
Spleen
WT
IμHABcl6
Hematossilin Eosin
Zoom
B220
RP
WP
B
LN
T cell zone
C
% Survival
WT (n=25)
IμHABcl6 (n=25)
****p<.0001
Time (days)
D
Human DLBCL-LN
Hematossilin Eosin
E
ER-TR7 αSMA
F
% ER-TR7 occupied area
G
Circle radius (μm)
GAP analysis

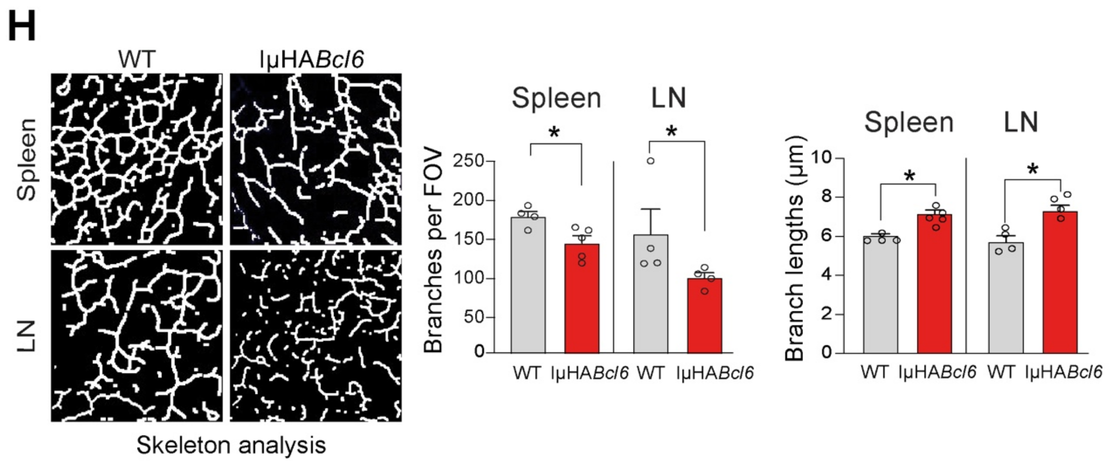

Supplemental Figure 2. FRCs are expanded and remodeled in IμHA*Bcl6* mice, a spontaneous murine model of DLBCL. (**A, B**) Histologic characterization of spleen (**A**) and LN (**B**) tissues from IμHA*Bcl6* mice. (**A**) Left panel: representative age-matched WT and IμHA*Bcl6* diseased spleens. Middle panels: H&E staining of WT and IμHA*Bcl6* spleens. Diseased tissues were characterized by white pulp (WP) expansion (and red pulp contraction), prevalence of large lymphocytes (zoomed in pictures), and tissue effacement by tumor B cells (right panels, B220 immunofluorescence). (**B**) Left panel: representative age-matched WT and IμHA*Bcl6* diseased LNs. Middle panels: H&E staining of WT and IμHA*Bcl6* LNs. DLBCL-infiltrated LNs were characterized by a loss of B/T cell zone compartmentalization (zoomed in picture) and tissue effacement by large tumor B cells (right panels, B220 immunofluorescence). (**C**) Kaplan-Meyer survival curves of IμHA*Bcl6* mice that developed lymphoma (n=25) vs WT age-matched animals (n=25). (**D**) Comparative H&E staining of a representative human DLBCL-LN biopsy showing loss of LN structure and expansion of large tumor B cells. Scale bar 1mm for large pictures, 50μm for zoomed in images. (**E**) Representative confocal images of ER-TR7 and αSMA immunofluorescence staining of spleens and LNs from WT and IμHA*Bcl6* mice. Scale bar=100μm. (**F**) Quantitation of the area occupied by ER-TR7$^+$ FRCs in spleen (WT n=6, IμHA*Bcl6* n=10) and LN (WT n=4, IμHA*Bcl6* n=6) tissues. (**G**) Left panel: representative gap analysis images of ER-TR7$^+$ FRCs from spleens and LNs of WT and IμHA*Bcl6* mice. Right panel: gap analysis quantification. (**H**) Left panels: representative skeletonized images of ER-TR7$^+$ FRCs from spleens and LNs of WT and IμHA*Bcl6* mice. Right panels: skeleton quantification: number of branches per FOV and branch lengths. Data in (**F**, **G**, **H**) are shown as mean ± SEM, *$P < .05$, ***$P<.001$, Mann–Whitney U-test.

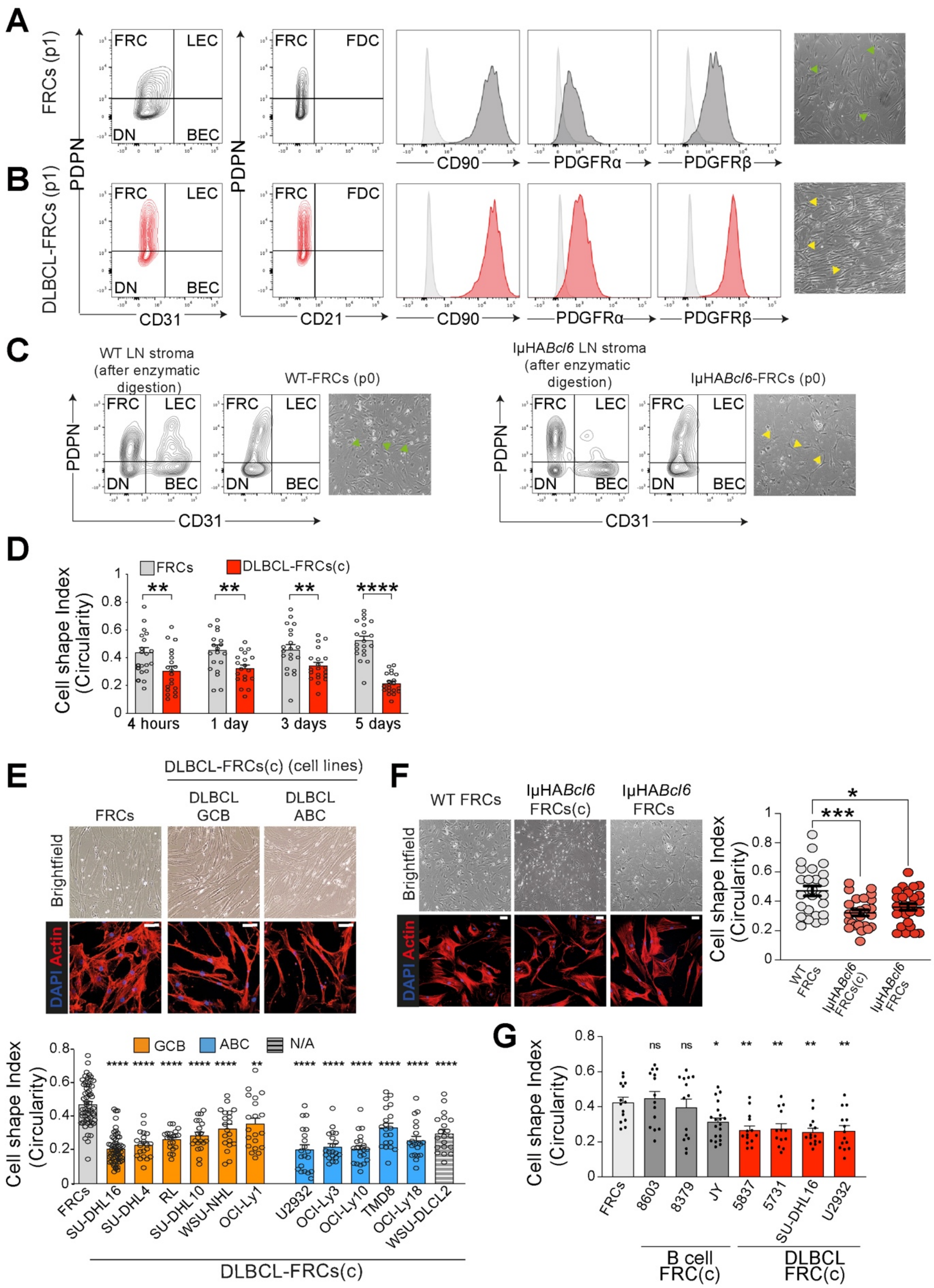

A
FRCs (p1)
FRC
LEC
DN
BEC
FDC
CD90
PDGFRα
PDGFRβ
B
DLBCL-FRCs (p1)
PDPN
CD31
CD21
C
WT LN stroma (after enzymatic digestion)
WT-FRCs (p0)
IμHABcl6 LN stroma (after enzymatic digestion)
IμHABcl6-FRCs (p0)
D
Cell shape Index (Circularity)
FRCs
DLBCL-FRCs(c)
4 hours
1 day
3 days
5 days
E
DLBCL-FRCs(c) (cell lines)
FRCs
DLBCL GCB
DLBCL ABC
Brightfield
DAPI Actin
F
WT FRCs
IμHABcl6 FRCs(c)
IμHABcl6 FRCs
GCB
ABC
N/A
SU-DHL16
SU-DHL4
RL
SU-DHL10
WSU-NHL
OCI-Ly1
U2932
OCI-Ly3
OCI-Ly10
TMD8
OCI-Ly18
WSU-DLCL2
DLBCL-FRCs(c)
G
8603
8379
JY
5837
5731
B cell FRC(c)
DLBCL FRC(c)

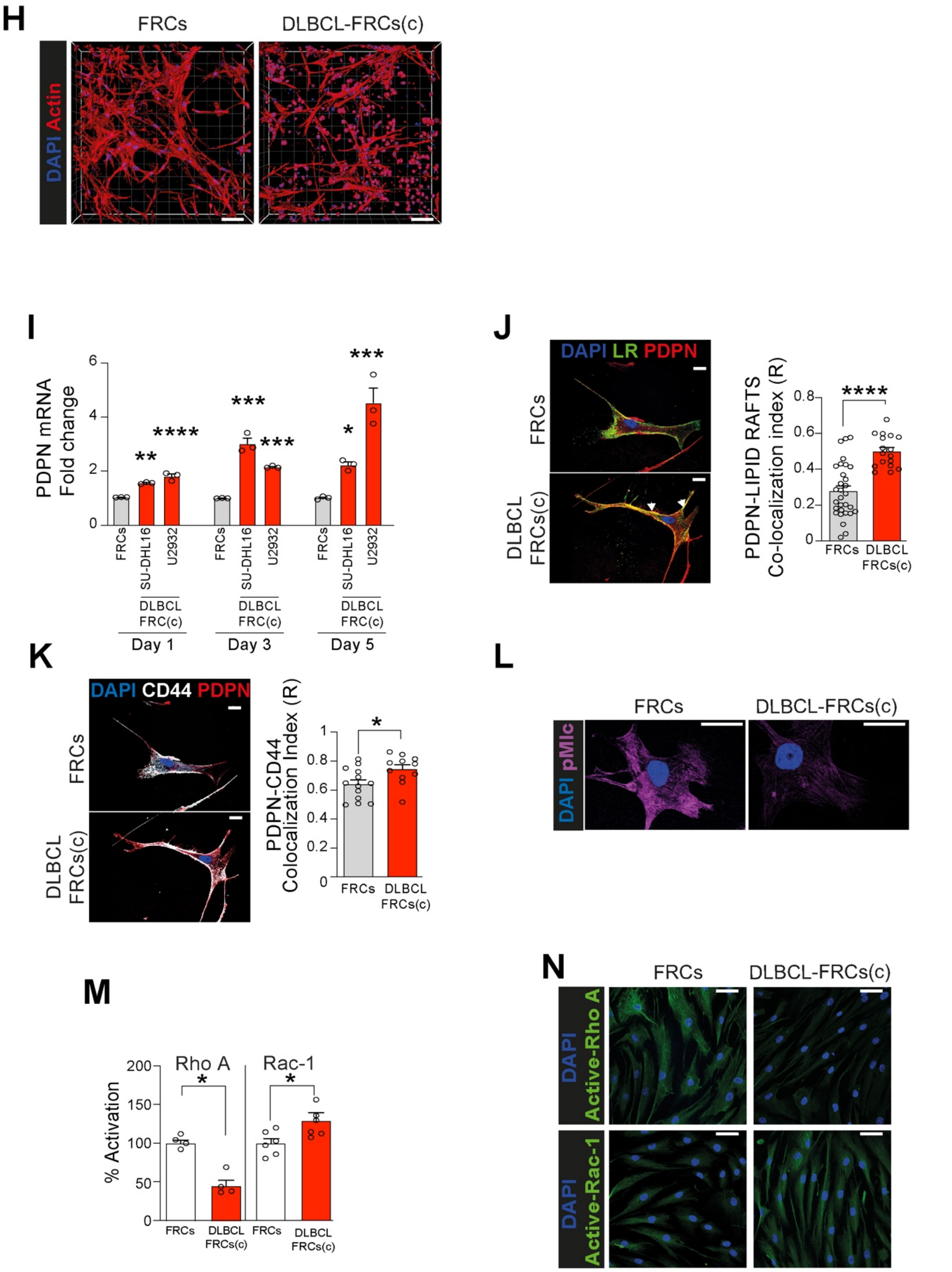

Supplemental Figure 3. DLBCL B cells activate FRCs in human and murine co-culture assays. (**A**, **B**) Phenotypic characterization of early passage (p1) human primary LN-derived FRCs (**A**) and DLBCL-FRCs(p) (**B**) using flow cytometry for the conventional FRCs markers (PDPN, CD90, PDGFRα/β) and brightfield microscopy (green arrows highlight contracted FRC morphology, yellow arrows highlight stretched DLBCL-FRC(p) morphology. DN refers to double negative stromal cells. FDC refers to follicular dendritic cells. (**C**) Immunophenotypic characterization of murine stromal cells from WT and IµHA*Bcl6* LNs immediately after enzymatic processing and at passage 0, together with brightfield microscopy

images of cultured WT- and IμHA*Bcl6*-FRCs (p0). (**D**) FRC cell shape analysis (circularity) of DLBCL-FRCs(c) (SU-DHL16 conditioning) compared to FRCs alone at the indicated co-culture time-points. Representative cell line data from n=2 GCB- and n=2 ABC-DLBCL independent cell lines. (**E**) Upper panel: Representative brightfield and confocal images of FRCs and DLBCL-FRCs(c) (SU-DHL16 (GCB type), U2932 (ABC type). Scale bar, 10μm. Bar chart shows cell shape quantification of DLBCL-FRCs(c) conditioned with a panel of 12 different DLBCL lines compared to FRCs alone following 5 days culture. (**F**) Representative brightfield (top panels) and confocal images (bottom panels) of WT-FRCs (n=3), IμHA*Bcl6*-FRCs(c) (n=4) and IμHA*Bcl6*-FRCs (n=2). Dot plot shows cell shape quantification (circularity). Scale bar, 50μm. Note: the representative WT-FRC and IμHA*Bcl6*-FRC bright field images are also presented in panel C above. (**G**) Cell shape quantification (circularity) of FRCs conditioned for 5 days with non-malignant B cells (B cell-FRCs(c): rLN-derived primary B cells, sample IDs 8603 and 8379 or EBV-transformed B lymphocyte cell line JY) or DLBCL cells (DLBCL-FRCs(c): primary DLBCL cells, patient ID 5837 and 5731 or DLBCL cell lines: SU-DHL16 and U2932). (**H**) Representative 3D confocal images of FRCs and DLBCL-FRCs(c) (SU-DHL16) cultured for 5 days. Note, co-mixed SU-DHL16 cells were not removed from 3D cultures. Scale bar=50μm. (**I**) Quantitative RT-PCR analysis of *PDPN* mRNA in FRCs and DLBCL-FRCs(c) (SU-DHL16 (GCB type), U2932 (ABC type)) at the time-points shown (reactions performed in triplicate). (**J, K**) Confocal images of lipid rafts (LR)-PDPN (**J**) and CD44-PDPN (**K**) localization studies in FRCs and DLBCL-FRCs(c) (U2932 cell line conditioning for 15 minutes). Note: images in J and K show the same representative stained FRCs. Bar charts show the co-localization index (Parsons). Scale bar=20μm. (**L**) Representative confocal images of phosphorylated-myosin light chain (pMLC) in FRCs and DLBCL-FRCs(c) (U2932 for 1 hour). Scale bar=50μm. (**M**) ELISA-based G-protein analysis (GLISA) of Rho A and Rac-1 activation in FRCs and DLBCL-FRCs(c) (U2932 for 1 hour). (**N**) Representative confocal images of active-Rho A (top panels) and active-Rac-1 (bottom panels) in FRCs and DLBCL-FRCs(c) (U2932 for 1 hour). Scale bar=50μm. (**D**, **E**, **H**, **I**, **J**, **K**, **L**, **M**, **N**) show a representative experiment from n=3 independent experiments. Data in (**D**, **E**, **F**, **G**, **I**, **J**, **K**, **M**) are shown as mean ± SEM, *****P* < .0001, ****P*<.001, ***P* < .01, **P* < .05, Mann–Whitney U-test (**D**, **F**, **J**, **K**, **M**) or one-way Anova with Tukey's multiple comparisons test (**E**, **F, G**).

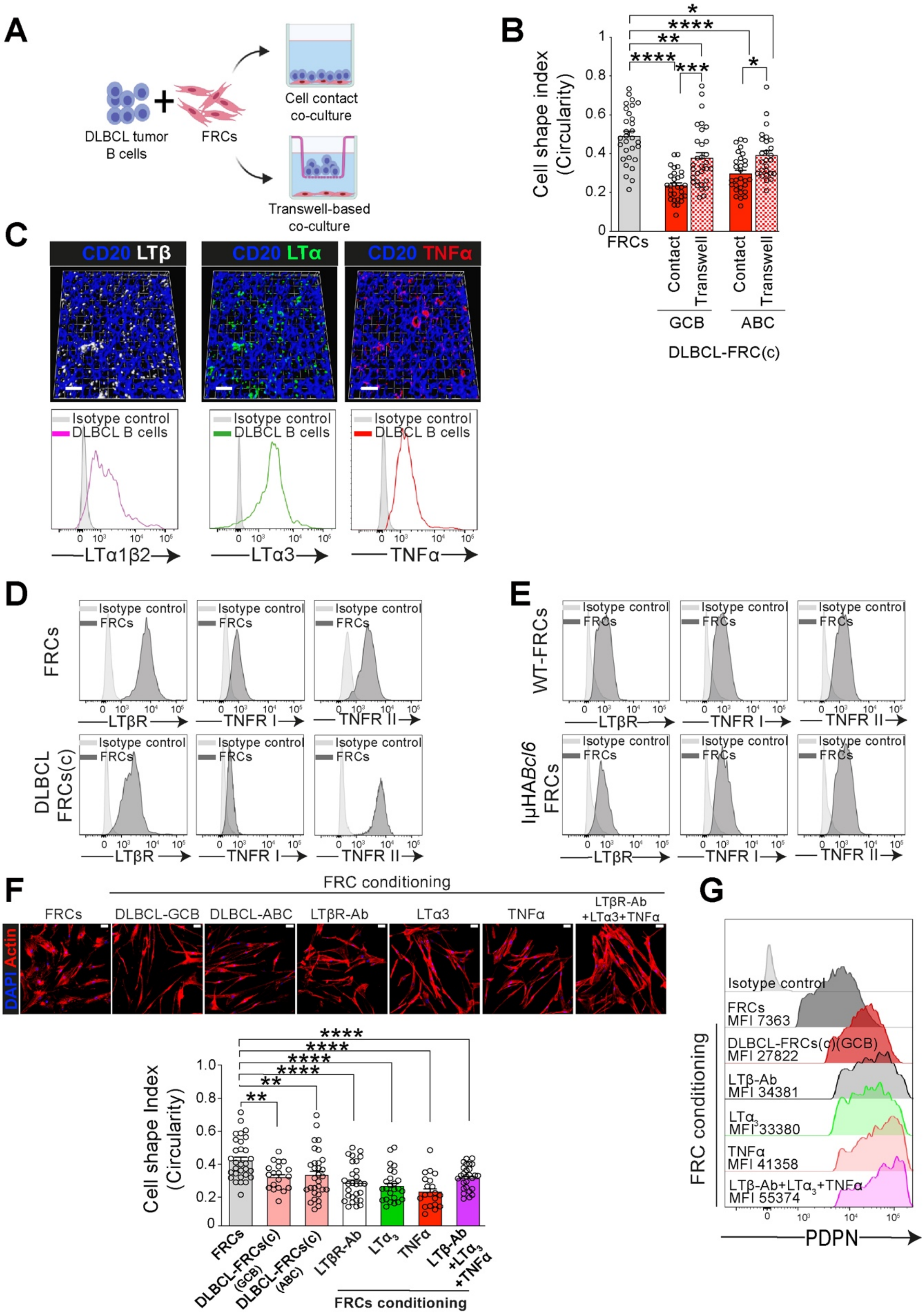
A
DLBCL tumor B cells
FRCs
Cell contact co-culture
Transwell-based co-culture
B
Cell shape index (Circularity)
FRCs
Contact
Transwell
GCB
ABC
DLBCL-FRC(c)
C
CD20 LTβ
CD20 LTα
CD20 TNFα
Isotype control
DLBCL B cells
LTα1β2
LTα3
TNFα
D
FRCs
DLBCL FRCs(c)
LTβR
TNFR I
TNFR II
E
WT-FRCs
IμHABcl6 FRCs
F
FRC conditioning
FRCs
DLBCL-GCB
DLBCL-ABC
LTβR-Ab
LTα3
TNFα
LTβR-Ab +LTα3+TNFα
DAPI Actin
Cell shape Index (Circularity)
DLBCL-FRCs(c) (GCB)
DLBCL-FRCs(c) (ABC)
FRCs conditioning
G
FRC conditioning
Isotype control
FRCs MFI 7363
DLBCL-FRCs(c)(GCB) MFI 27822
LTβ-Ab MFI 34381
LTα3 MFI 33380
TNFα MFI 41358
LTβ-Ab+LTα3+TNFα MFI 55374
PDPN

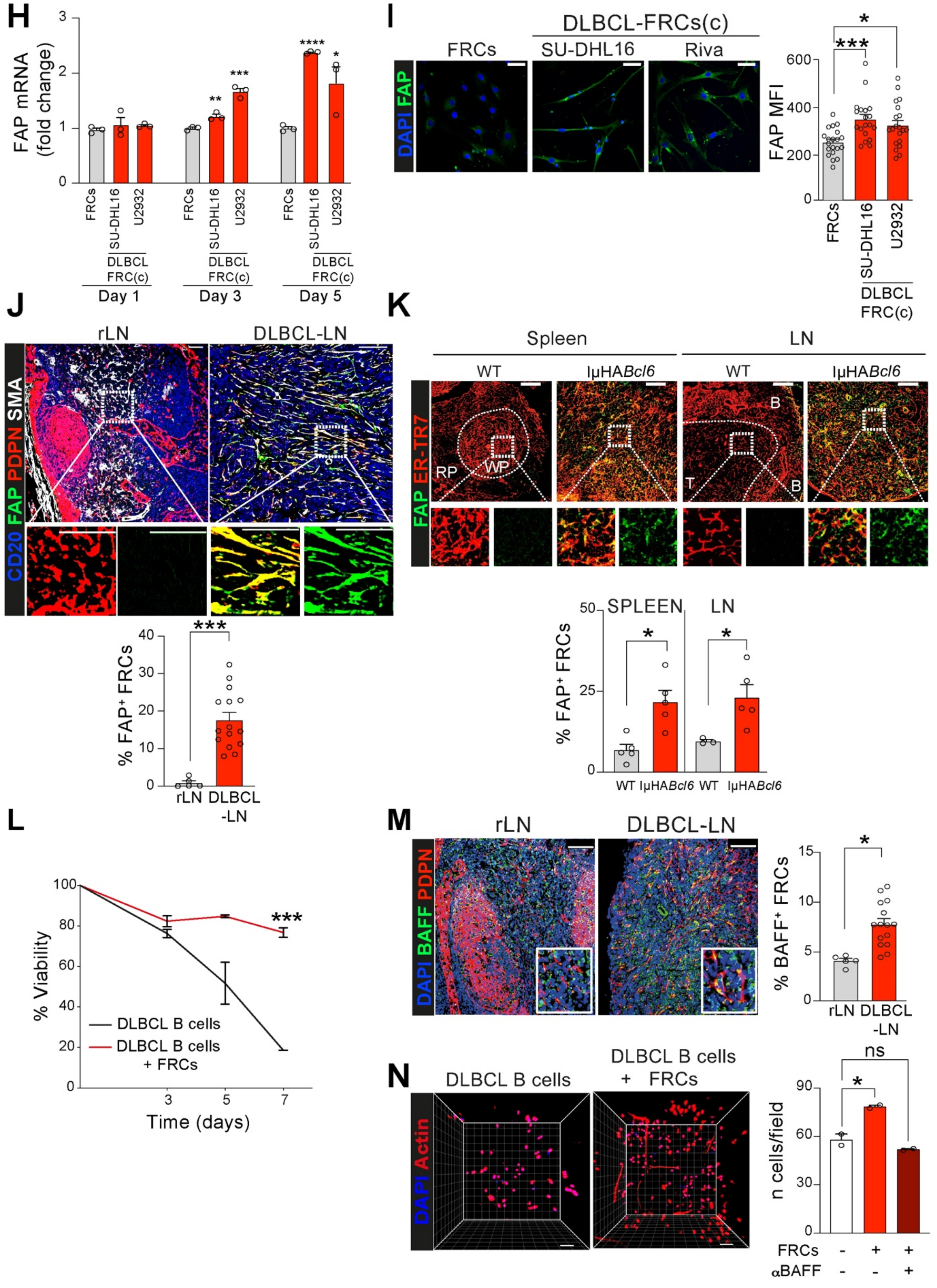

Supplemental Figure 4. Characterization of tumor B cell-FRC crosstalk in DLBCL. (**A**) Cartoon showing the FRC conditioning approaches (direct cell contact or transwell co-culture). (**B**) Cell shape analysis of FRCs and DLBCL-FRCs(c) (GCB-DLBCL: SU-DHL16, ABC-DLBCL: U2932) comparing cell contact and transwell co-culture for 5 days. (**C**) Expression of $LT\alpha_1\beta_2$, $LT\alpha_3$, and $TNF\alpha$ in DLBCL B cells. Upper panel:

Representative 3D confocal images of multiplex RNAscope™ for LTβ (*LTB*), LTα (*LTA*) and TNFα (*TNFA*) transcripts expressed by CD20$^+$ tumor cells in human DLBCL biopsies. Scale bar, 50μm. Lower panels: representative flow histograms showing the expression of surface $LT\alpha_1\beta_2$ and intracellular $LT\alpha_3$, and TNFα in primary DLBCL B cells. (**D, E**) Representative flow histograms of surface LTβR, TNFRI and TNFRII on FRCs (upper panels) and DLBCL-FRCs(c) (SU-DHL16) (lower panels) (**D**) and on WT-FRCs (upper panels) and IμHA*Bcl6*-FRCs (lower panels) (**E**). (**F**) Cell shape analysis of FRCs alone and DLBCL-FRCs(c) (GCB-DLBCL: SU-DHL16, ABC-DLBCL: U2932) or FRC conditioning with LTβR activating antibody, recombinant $LT\alpha_3$ or TNFα treatment for 3 days. Upper panels: representative confocal images, lower panel: cell morphology quantification (circularity). Scale bar, 50μm. (**G**) Representative histograms of surface PDPN expression on FRCs alone or conditioned for 3 days with SU-DHL16, or LTβR activating antibody, or recombinant $LT\alpha_3$ or TNFα (or together). FRCs are gated on the PDPN$^+$ population. (**H**) RT-PCR analysis of *FAP* mRNA in FRCs and DLBCL-FRCs(c) (SU-DHL16 (GCB), U2932 (ABC) at 1, 3 and 5 day time-points. (**I**) Confocal images and analysis of FAP expression by FRCs and DLBCL-FRCs(c) (SU-DHL16 (GCB), U2932 (ABC). Scale bar, 50μm. (**J**) Representative confocal images of rLN (n=5) and whole DLBCL-LN (n=15) biopsies stained for CD20, PDPN and FAP. Bar chart shows FAP$^+$ FRC frequency analysis across the network. Scale bars: main and zoomed in images 100μm. (**K**) Representative confocal images of spleens (WT n=5, IμHA*Bcl6* n=5) and LNs (WT n=3, IμHA*Bcl6* n=5) stained for ER-TR7 and FAP with frequency of network analysis below. B: B-cell zone, T: T-cell zone, RP: red pulp, WP: white pulp. Scale bar, 100μm. Note: WT LN image shows the same tissue sample presented in Supplementary Figure 2E that was stained as part of a multi-marker IF panel. (**L**) Flow cytometric viability assessment (Annexin V and ToPro3 staining) of the SU-DHL16 cell line cultured alone or co-mixed in the presence of FRCs in serum starved conditions (time points: 3, 5, 7 days). (**M**) Representative confocal images of rLN (n=5) and whole DLBCL-LN (n=15) stained for DAPI, PDPN and B-cell activating factor, BAFF. Bar chart shows the % of BAFF$^+$ FRCs. Scale bar, 100μm. (**N**) Representative volume rendered confocal images of primary DLBCL B cells cultured in 3D gels alone or co-mixed in the presence of FRCs for 5 days. Bar chart shows the number of primary DLBCL cells/3 Z-stack image areas. Treatment with a control isotype antibody or a BAFF-neutralizing antibody indicated. (**B**, **F**, **H**, **I**, **L**) show a representative experiment from n=3 independent experiments. Data in (**B**, **F**, **H**, **I**, **J**, **K**, **L**, **M**, **N**) are shown as mean ± SEM, *****P* < .0001, ****P*<.001, ***P* < .01, **P* < .05, one-way Anova with Tukey's multiple comparisons test (**B**, **F**, **I)** or Mann–Whitney U-test (**H**, **J**, **K**, **L**, **M**, **N**).

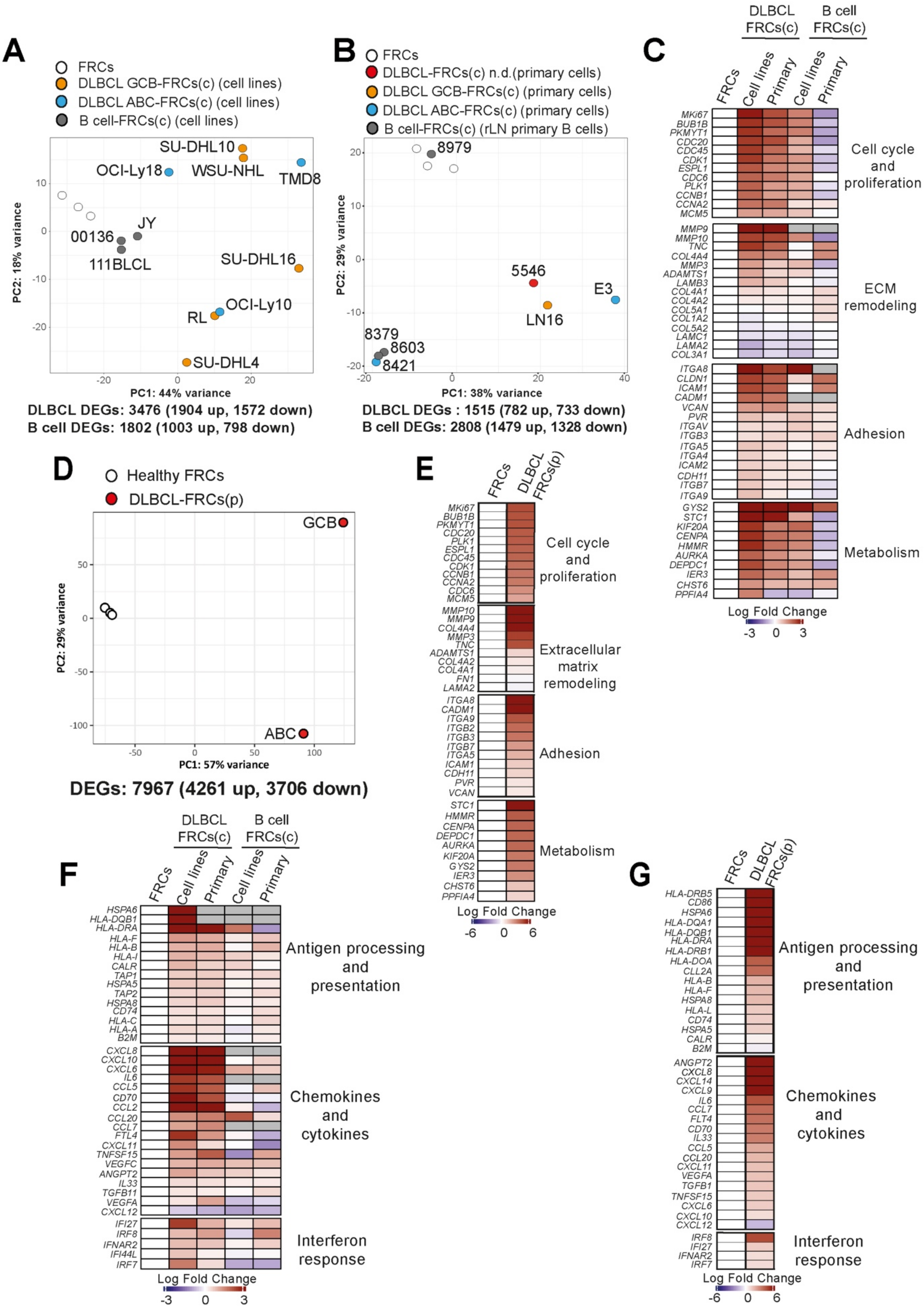

A
FRCs
DLBCL GCB-FRCs(c) (cell lines)
DLBCL ABC-FRCs(c) (cell lines)
B cell-FRCs(c) (cell lines)
SU-DHL10
OCI-Ly18
WSU-NHL
TMD8
JY
00136
111BLCL
SU-DHL16
OCI-Ly10
RL
SU-DHL4
PC1: 44% variance
PC2: 18% variance
DLBCL DEGs: 3476 (1904 up, 1572 down)
B cell DEGs: 1802 (1003 up, 798 down)
B
FRCs
DLBCL-FRCs(c) n.d.(primary cells)
DLBCL GCB-FRCs(c) (primary cells)
DLBCL ABC-FRCs(c) (primary cells)
B cell-FRCs(c) (rLN primary B cells)
8979
5546
E3
LN16
8379
8603
8421
PC1: 38% variance
PC2: 29% variance
DLBCL DEGs : 1515 (782 up, 733 down)
B cell DEGs: 2808 (1479 up, 1328 down)
C
DLBCL FRCs(c)
B cell FRCs(c)
FRCs
Cell lines
Primary
Cell lines
Primary
MKi67 BUB1B PKMYT1 CDC20 CDC45 CDK1 ESPL1 CDC6 PLK1 CCNB1 CCNA2 MCM5
Cell cycle and proliferation
MMP9 MMP10 TNC COL4A4 MMP3 ADAMTS1 LAMB3 COL4A1 COL4A2 COL5A1 COL1A2 COL5A2 LAMC1 LAMA2 COL3A1
ECM remodeling
ITGA8 CLDN1 ICAM1 CADM1 VCAN PVR ITGAV ITGB3 ITGA5 ITGA4 ICAM2 CDH11 ITGB7 ITGA9
Adhesion
GYS2 STC1 KIF20A CENPA HMMR AURKA DEPDC1 IER3 CHST6 PPFIA4
Metabolism
Log Fold Change
-3 0 3
D
Healthy FRCs
DLBCL-FRCs(p)
GCB
ABC
PC1: 57% variance
PC2: 29% variance
DEGs: 7967 (4261 up, 3706 down)
E
FRCs
DLBCL FRCs(p)
MKi67 BUB1B PKMYT1 CDC20 PLK1 ESPL1 CDC45 CDK1 CCNB1 CCNA2 CDC6 MCM5
Cell cycle and proliferation
MMP10 MMP9 COL4A4 MMP3 TNC ADAMTS1 COL4A2 COL4A1 FN1 LAMA2
Extracellular matrix remodeling
ITGA8 CADM1 ITGA9 ITGB2 ITGB3 ITGB7 ITGA5 ICAM1 CDH11 PVR VCAN
Adhesion
STC1 HMMR CENPA DEPDC1 AURKA KIF20A GYS2 IER3 CHST6 PPFIA4
Metabolism
Log Fold Change
-6 0 6
F
DLBCL FRCs(c)
B cell FRCs(c)
FRCs
Cell lines
Primary
Cell lines
Primary
HSPA6 HLA-DQB1 HLA-DRA HLA-F HLA-B HLA-I CALR TAP1 HSPA5 TAP2 HSPA8 CD74 HLA-C HLA-A B2M
Antigen processing and presentation
CXCL8 CXCL10 CXCL6 IL6 CCL5 CD70 CCL2 CCL20 CCL7 FTL4 CXCL11 TNFSF15 VEGFC ANGPT2 IL33 TGFB11 VEGFA CXCL12
Chemokines and cytokines
IFI27 IRF8 IFNAR2 IFI44L IRF7
Interferon response
Log Fold Change
-3 0 3
G
FRCs
DLBCL FRCs(p)
HLA-DRB5 CD86 HSPA6 HLA-DQA1 HLA-DQB1 HLA-DRA HLA-DRB1 HLA-DOA CLL2A HLA-B HLA-F HSPA8 HLA-L CD74 HSPA5 CALR B2M
Antigen processing and presentation
ANGPT2 CXCL8 CXCL14 CXCL9 IL6 CCL7 FLT4 CD70 IL33 CCL5 CCL20 CXCL11 VEGFA TGFB1 TNFSF15 CXCL6 CXCL10 CXCL12
Chemokines and cytokines
IRF8 IFI27 IFNAR2 IRF7
Interferon response
Log Fold Change
-6 0 6

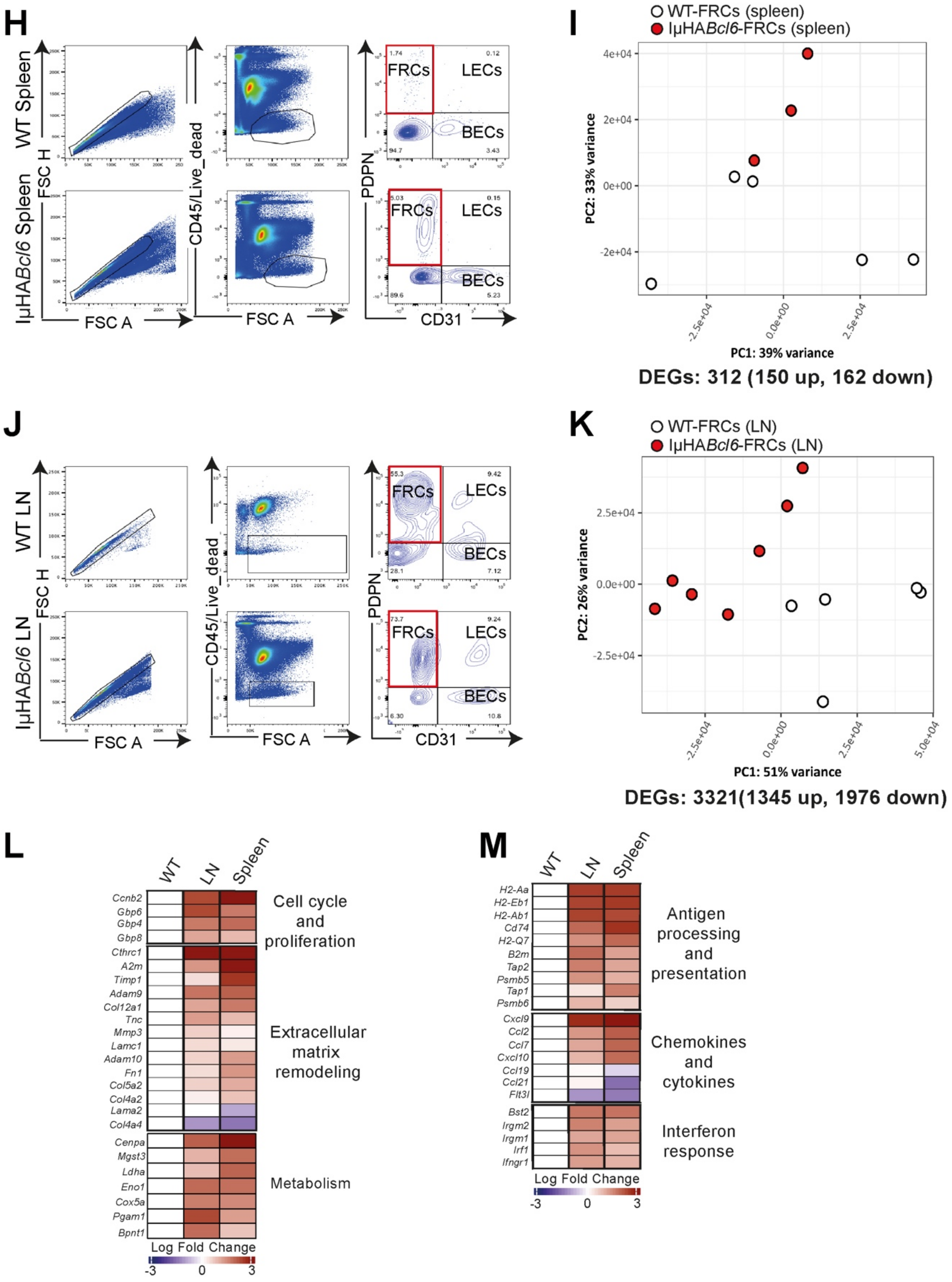
H
WT Spleen
IμHABcl6 Spleen
FSC H
FSC A
CD45/Live_dead
PDPN
CD31
FRCs
LECs
BECs
I
WT-FRCs (spleen)
IμHABcl6-FRCs (spleen)
PC2: 33% variance
PC1: 39% variance
DEGs: 312 (150 up, 162 down)
J
WT LN
IμHABcl6 LN
K
WT-FRCs (LN)
IμHABcl6-FRCs (LN)
PC2: 26% variance
PC1: 51% variance
DEGs: 3321(1345 up, 1976 down)
L
WT
LN
Spleen
Ccnb2
Gbp6
Gbp4
Gbp8
Cthrc1
A2m
Timp1
Adam9
Col12a1
Tnc
Mmp3
Lamc1
Adam10
Fn1
Col5a2
Col4a2
Lama2
Col4a4
Cenpa
Mgst3
Ldha
Eno1
Cox5a
Pgam1
Bpnt1
Cell cycle and proliferation
Extracellular matrix remodeling
Metabolism
Log Fold Change
M
H2-Aa
H2-Eb1
H2-Ab1
Cd74
H2-Q7
B2m
Tap2
Psmb5
Tap1
Psmb6
Cxcl9
Ccl2
Ccl7
Cxcl10
Ccl19
Ccl21
Flt3l
Bst2
Irgm2
Irgm1
Irf1
Ifngr1
Antigen processing and presentation
Chemokines and cytokines
Interferon response
Log Fold Change

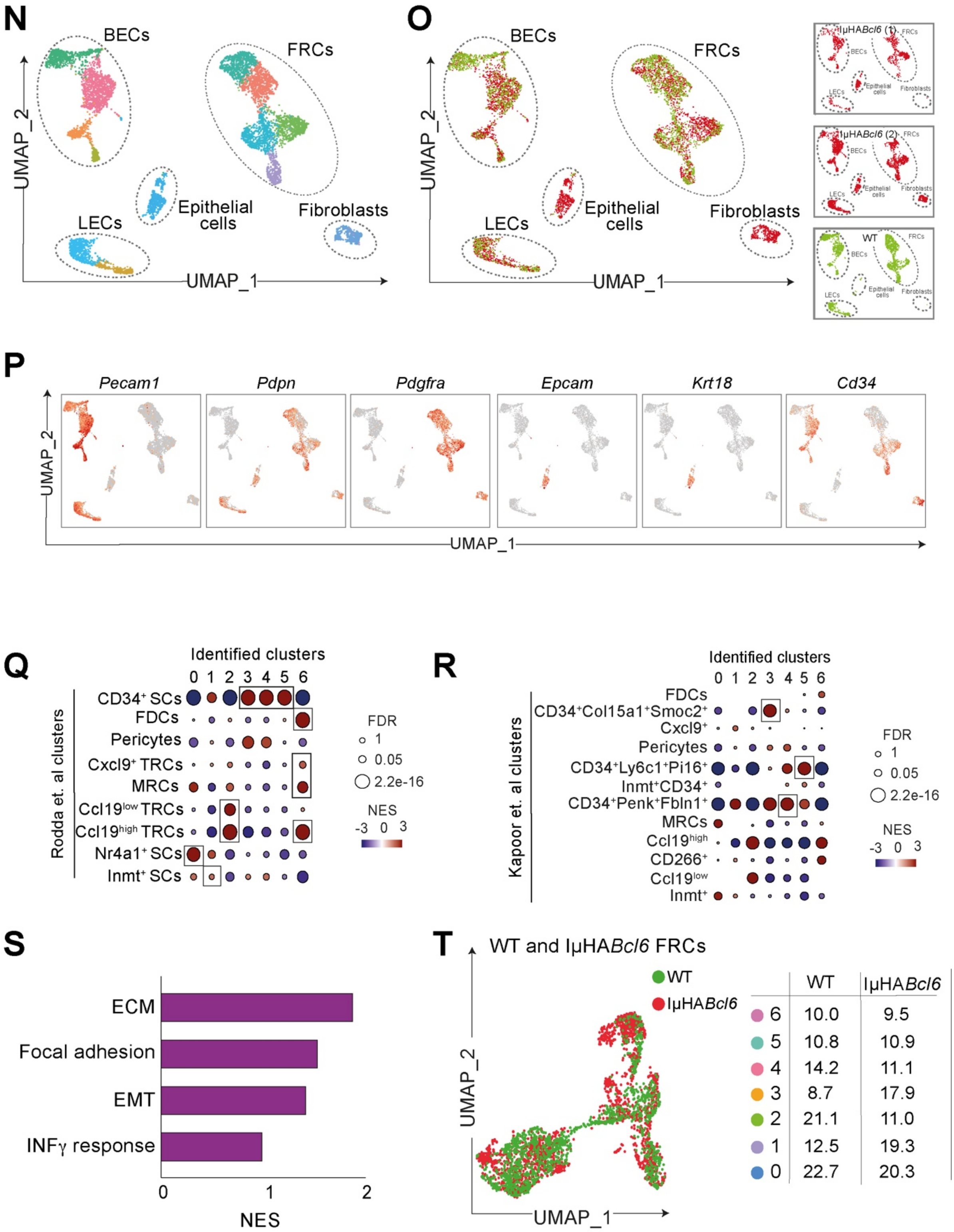

Supplemental Figure 5. Bulk and scRNA-seq transcriptome analysis of human and murine DLBCL-FRCs. (**A**) Principal component analysis (PCA) plot of FRCs (n=3) (white dots), DLBCL-FRCs(c) (following 48 hours conditioning with DLBCL-GCB cell lines (orange dots: SU-DHL4, SU-DHL-10, SU-DHL16, RL or WSH-NHL), DLBCL-ABC cell lines (blue dots OCI-Ly10, TMD8 or OCI-Ly18) and B cell lines (grey dots: JY, 00136 and 111BCL) in transwell culture). Numbers of significantly up- and down-regulated genes (DEGs) (adjusted p value ≤0.05) are shown for the comparison of DLBCL-FRCs(c)(cell lines) *vs* FRCs and B cell-FRCs(c)(cell lines) *vs* FRCs. (**B**) PCA plot of FRCs (n=3) (white dots), DLBCL-FRCs(c) (following 48 hours

conditioning with primary DLBCL B cells) (n=4 primary patient samples including ABC (blue dots) and GCB (orange dots) COO or not determined (red dot) as indicated, sample IDs E3, LN16, 5546, 8421) and B cell-FRCs(c) (conditioning with primary rLN-derived B cells, n=3 shown with grey dots, sample IDs: 8379, 8603, 8979). Numbers of up- and down-regulated DEGs (adjusted p value ≤0.05) are shown for the comparison of DLBCL-FRCs(c) (primary DLBCL B cells) *vs* FRCs and B cell-FRCs(c) (primary rLN B cells) *vs* FRCs. (**C**) Heatmap showing the log fold change of leading-edge genes identified by GSEA as major contributors to the enrichment of fibroblast activation pathways and differentially expressed in DLBCL-FRCs(c) (cell lines or primary DLBCL B cells) and B cell-FRCs(c) (cell lines or primary rLN B cells) vs FRCs. (**D**) PCA plot of FRCs (n=3) vs DLBCL-FRCs(p) (n=2 independent patient tissue samples). Numbers of significantly up- and down-regulated DEGs (adjusted p value ≤0.05) are shown for the comparison of DLBCL-FCRs(p) vs FRCs. (**E**) Heatmap showing the log fold change of leading-edge genes identified by GSEA as major contributors to the enrichment of fibroblast activation pathways and differentially expressed in DLBCL-FRCs(p) vs FRCs. (**F**) Heatmap showing the log fold change of leading-edge genes belonging to immunologically relevant pathways (identified as enriched in GSEA) and differentially expressed in DLBCL-FRCs(c) (cell lines or primary DLBCL B cells) *vs* FRCs and B cell-FRCs(c) (cell lines or primary rLN B cells) *vs* FRCs. (**G**) Heatmap showing the log fold change of leading-edge genes belonging to immunologically relevant pathways (identified as enriched by GSEA) and differentially expressed in DLBCL-FRCs(p) vs FRCs. (**H**) Gating strategy for the sorting of primary FRCs isolated from the spleens of age-matched WT and IμHA*Bcl6* mice. (**I**) PCA plot of WT-FRCs (n=5) *vs* IμHA*Bcl6*-FRCs (n=3) isolated from murine spleens. Numbers of significantly up- and down-regulated DEGs (adjusted p value ≤0.05) are shown for the comparison of IμHA*Bcl6*-FRCs *vs* WT-FRCs. (**J**) Gating strategy for the sorting of primary FRCs isolated from LNs of WT and IμHA*Bcl6* mice. (**K**) PCA plot of WT-FRCs (n=5) vs IμHA*Bcl6*-FRCs (n=7) isolated from murine LNs. Numbers of significantly up- and down-regulated DEGs (adjusted p value <=0.05) are shown for the comparison of IμHA*Bcl6*-FRCs vs WT-FRCs. (**L**) Heatmap showing the log fold change of leading-edge genes identified by GSEA as drivers of the enrichment of fibroblast activation pathways and differentially expressed in WT-FRCs vs IμHA*Bcl6*-FRCs in LN and spleen. (**M**) Heatmap showing the log fold change of leading-edge genes identified by GSEA as drivers of the enrichment of immunologically relevant pathways and differentially expressed in WT-FRCs vs IμHA*Bcl6*-FRCs in LN and spleen. (**N**) UMAP analysis relative to 7,465 stromal cells isolated from the LNs of WT (n=1) and IμHA*Bcl6* mice (n=2). Each color indicates a specific cluster. 5 main cellular populations were identified based on the expression of the lineage markers shown in panel (**P**): BECs, LECs, FRCs, a cluster of PDPN$^+$ epithelial cells and an additional cluster of PDPN$^+$ fibroblasts. The FRC population was subsequently analyzed (FRC-reclustered analysis) in this present study. (**O**) UMAP analysis showing cell distribution across WT (green) and IμHA*Bcl6* (red) samples. The additional fibroblast cluster was detected in only 1 of the 3 analyzed samples and for this reason it was not included for the downstream FRC reclustered analysis. (**P**) Feature plots showing the expression of selected lineage genes across LN stroma cellular clusters. (**Q**, **R**) GSEA results showing the transcriptional similarities between the identified FRC clusters (FRC-reclustered analysis) from our dataset and those previously published. Dot colors show the normalized enrichment score (NES), dot size the FDR. (**Q**) (20), (**R**) (21). (**S**) GSEA results of the pathways enriched in IμHA*Bcl6*-FRCs compared to WT-FRCs. Histograms show the NES. (**T**) Distribution of the FRC clusters in IμHA*Bcl6* vs WT LNs. Left panel: UMAP plot of the integrated FRC data. Each dot represents a single cell belonging either to WT (green) or to the IμHA*Bcl6* FRCs (red). Right panel: frequency of each identified FRC cluster in WT or IμHA*Bcl6* samples.

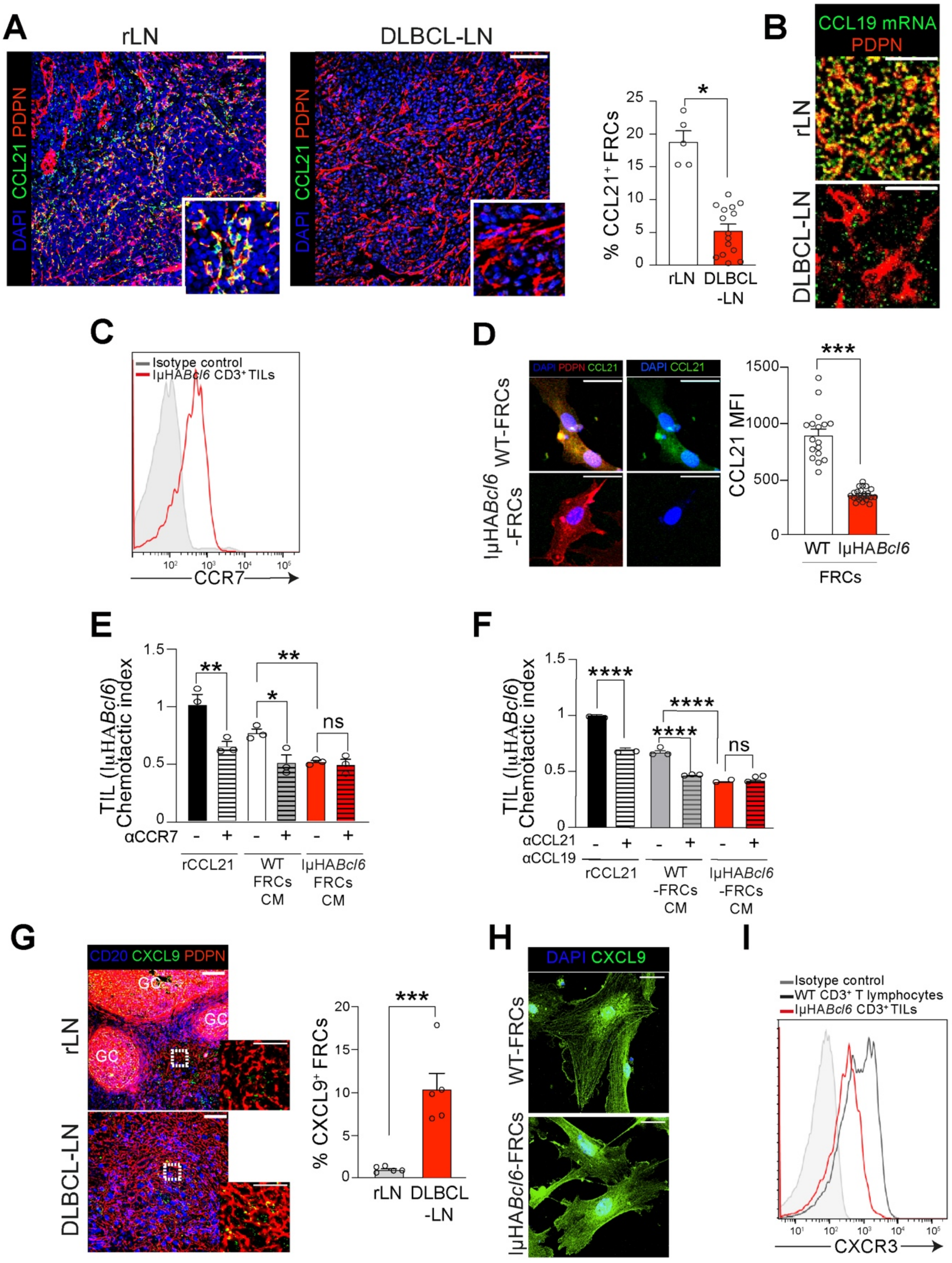
A
rLN
DLBCL-LN
DAPI CCL21 PDPN
% CCL21+ FRCs
rLN
DLBCL -LN
B
CCL19 mRNA PDPN
rLN
DLBCL-LN
C
Isotype control
IμHABcl6 CD3+ TILs
CCR7
D
DAPI PDPN CCL21
DAPI CCL21
WT-FRCs
IμHABcl6 -FRCs
CCL21 MFI
WT IμHABcl6
FRCs
E
TIL (IμHABcl6) Chemotactic index
αCCR7
rCCL21
WT FRCs CM
IμHABcl6 FRCs CM
F
TIL (IμHABcl6) Chemotactic index
αCCL21
αCCL19
rCCL21
WT -FRCs CM
IμHABcl6 -FRCs CM
G
CD20 CXCL9 PDPN
rLN
DLBCL-LN
GC
% CXCL9+ FRCs
rLN DLBCL -LN
H
DAPI CXCL9
WT-FRCs
IμHABcl6-FRCs
I
Isotype control
WT CD3+ T lymphocytes
IμHABcl6 CD3+ TILs
CXCR3

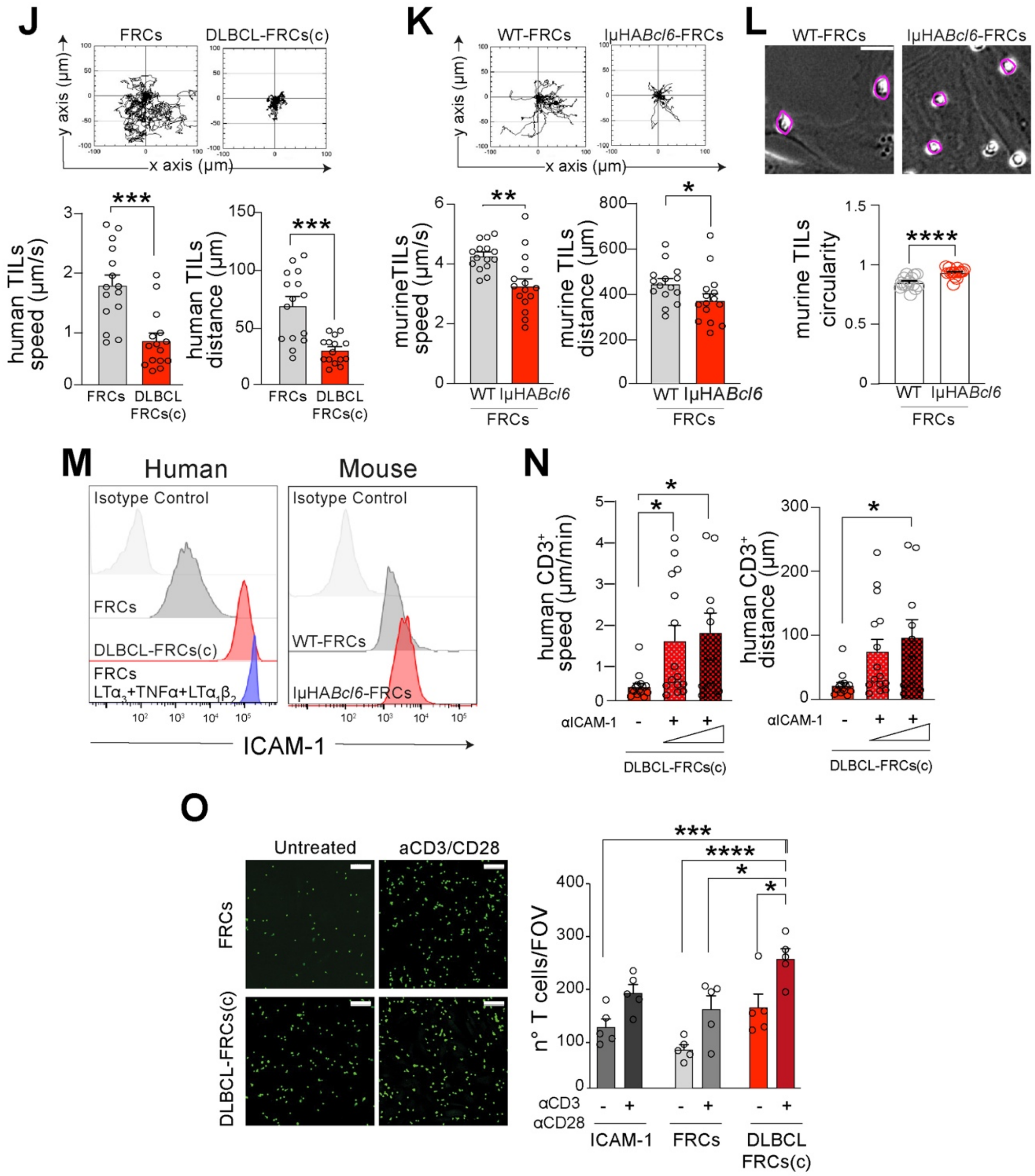
J
FRCs
DLBCL-FRCs(c)
y axis (µm)
x axis (µm)
human TILs speed (µm/s)
human TILs distance (µm)
FRCs
DLBCL FRCs(c)
K
WT-FRCs
IµHABcl6-FRCs
murineTILs speed (µm/s)
murine TILs distance (µm)
WT IµHABcl6
FRCs
L
WT-FRCs
IµHABcl6-FRCs
murine TILs circularity
WT IµHABcl6
FRCs
M
Human
Mouse
Isotype Control
FRCs
DLBCL-FRCs(c)
FRCs LTα3+TNFα+LTα1β2
WT-FRCs
IµHABcl6-FRCs
ICAM-1
N
human CD3+ speed (µm/min)
human CD3+ distance (µm)
αICAM-1
DLBCL-FRCs(c)
O
Untreated
aCD3/CD28
FRCs
DLBCL-FRCs(c)
n° T cells/FOV
αCD3
αCD28
ICAM-1
FRCs
DLBCL FRCs(c)

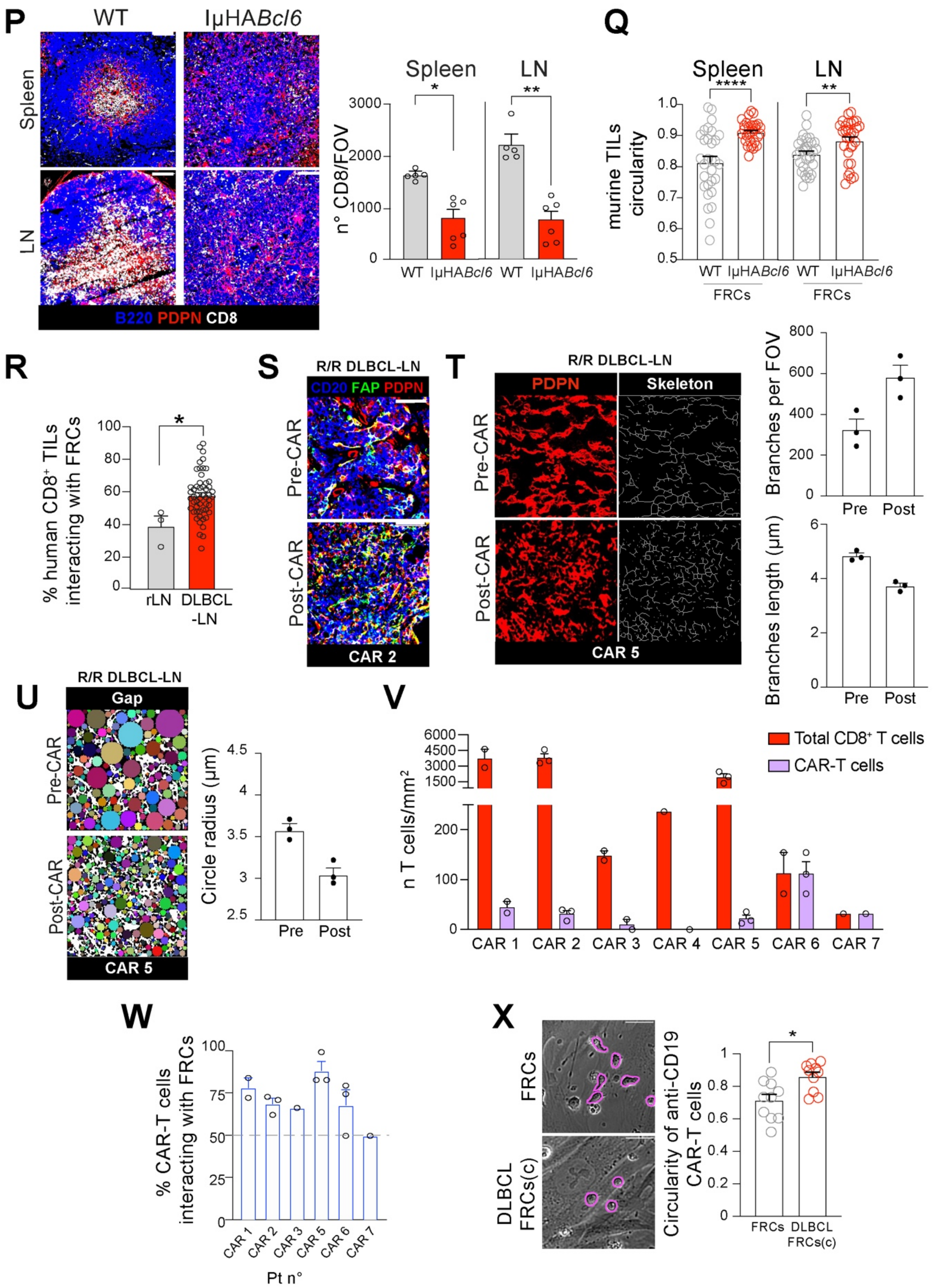

Supplemental Figure 6. DLBCL-FRCs exhibit diminished capacity to support T lymphocyte migration. (**A**) Representative confocal images of human rLN (n=5) and whole DLBCL-LNs (n=15) stained for DAPI, CCL21 and PDPN. Scale bar, 100μm. Bar chart shows the percentage of $CCL21^{+}$ FRCs. Note: rLN image shows the same tissue sample presented in Supplementary Figure 1G that was stained as part of a multi-marker IF panel. (**B**) Representative RNAscope confocal images for the FRC-associated *Ccl19* transcript in rLN (n=5) and whole DLBCL-LNs (n=10). Scale bar, 50μm. (**C**) Flow histogram showing

expression CCR7 expression by $CD3^+$ TILs (IµHA*Bcl6*) (representative sample, n=5 IµHA*Bcl6*). (**D**) Representative confocal images of LN-derived WT-FRCs (n=3) and IµHA*Bcl6*-FRCs (n=3) in culture (48 hours after isolation) and stained for DAPI, CCL21 and PDPN. Scale bar, 50µm. (**E, F**) $CD3^+$ TIL (IµHA*Bcl6*) chemotaxis towards recombinant CCL21 (rCCL21, positive control) or conditioned media (CM) from WT-FRCs or IµHA*Bcl6*-FRCs in presence of isotype control or a CCR7 blocking (**E**) or CCL19/CCL21 neutralizing antibodies (**F**). (**G**) Representative confocal images of rLN (n=5) and whole DLBCL-LNs (n=5) stained for DAPI, CXCL9 and PDPN. GC: germinal center. Scale bar, 100µm. Bar chart: percentage of $CXCL9^+$ FRCs. (**H**) Representative confocal images of LN-derived WT-FRCs and IµHA*Bcl6*-FRCs in culture (48 hours after isolation) and stained for DAPI and CXCL9. Scale bar, 50µm. (**I**) Flow histogram showing expression CXCR3 expression by $CD3^+$ TIL (IµHA*Bcl6*) in comparison to WT $CD3^+$ T lymphocytes (representative sample, n=5 mice samples). (**J**) Human $CD3^+$ TIL motility on a monolayer (2D) of FRCs and DLBCL-FRCs(c) (primary DLBCL cells with autologous TILs). Upper panels show representative TIL migratory tracks. Charts show the quantification of TIL migratory speed and distance (lower panel). (**K**) Motility of $CD3^+$ TILs (IµHA*Bcl6*) on a monolayer (2D) of WT-FRCs or IµHA*Bcl6* FRCs. Upper panels show representative TIL migratory tracks. Charts show the quantification of TIL migratory speed and distance (lower panel). (**L**) $CD3^+$ TIL (IµHA*Bcl6*) cell shape (circularity) analysis during 2D motility (K). Upper panels show a still frame image of motile TILs (representative TIL morphology highlighted in purple). Quantification of TIL cell shape is shown in the chart (higher values represent increased circularity). (**M**) Left flow histogram: representative expression analysis of ICAM-1 on FRCs, DLBCL-FRCs(c) (SU-DHL16), and FRCs treated with LTβR activating antibody, recombinant $LT\alpha_3$ and TNFα for 3 days. FRCs were gated on the $PDPN^+$ population. Right flow histogram: ICAM-1 expression on WT-FRCs and IµHA*Bcl6*-FRCs. (**N**) Human $CD3^+$ T cell motility on FRCs and DLBCL-FRCs(c) (SU-DHL16) pre-treated with isotype control (-) or an ICAM-1 blocking antibody (5 ng/ml and 50 ng/ml). Bar charts show TIL speed and distance. (**O**) Representative confocal images showing human $CD3^+$ T cell adhesion (unstimulated or pre-stimulated with anti-CD3/CD28 antibodies) on a monolayer of human FRCs or DLBCL-FRCs(c) (SU-DHL16). Bar chart shows the number of $CD3^+$/FOV across the different conditions. ICAM-1 coated plate was used as positive control. (**P**) Representative confocal images of WT and IµHA*Bcl6* spleen and LN tissues stained for B220, PDPN and CD8. Scale bar, 100µm. Bar chart shows quantification of the number of $CD8^+$/FOV. Spleen: WT n=5, IµHA*BCL6* n=6, LN: WT n=5, IµHA*Bcl6* n=6. (**Q**) Cell shape measurement (circularity) of $CD8^+$ TILs in one representative murine spleen and LN from WT and IµHA*Bcl6* mice. (**R**) IMC quantification of the % $CD8^+$ TILs interacting with FRCs in rLN (n=3) and DLBCL-LNs (n=53). (**S**) Representative confocal images of the FRC network in DLBCL tissues pre- and post-(at relapse) CAR T-cell infusion stained for FAP. Scale bar, 100µm. (**T**) $PDPN^+$ network characterization in one DLBCL patient (Pt5), pre- and post- (partial remission) CAR T-cell infusion, using skeleton analysis. Left panels: original PDPN signal, right panels: skeletonized PDPN signals. Bar charts show skeleton quantification: number of branches per FOV and branch lengths. (**U**) Gap analysis of the FRC network in one representative DLBCL patient (Pt5), pre- and post- (partial remission) CAR T-cell infusion. The graph shows the radii of the circles that fit into the gaps of the PDPN network. (**V**) Quantification of endogenous $CD8^+$ TILs and CAR-T cells in the 7 post-infusion biopsies (Pt: Patient). (**W**) Quantification of the percentage of CAR T-cells interacting with FRCs in post-CAR T-cell infusion patient biopsies (n=7). (**X**) Measurement of anti-CD19 CAR-T cell shape (circularity) during 2D motility on FRCs and DLBCL-FRCs(c) (SU-DHL16). Left panels show a still image of the 2D motility, CAR T-cell shape is highlighted in purple. Right graph shows circularity quantification. (**E**, **F**, **H**, **J**, **K**, **L**, **M**, **N**, **O**, **Q**, **V**) representative data from n=3 independent experiments. Data in (**A**, **D**-**G**, **J**-**L**, **N**-**R**, **T**-**X**) are shown as mean ± SEM, $****P < .0001$, $***P<.001$, $**P < .01$, $*P < .05$, one-way Anova with Tukey's multiple comparisons test (**E**, **F**, **N**, **O**) or Mann–Whitney U-test (**A**, **D**, **G**, **J**-**L**, **P**-**R**, **T**, **U**, **W**).

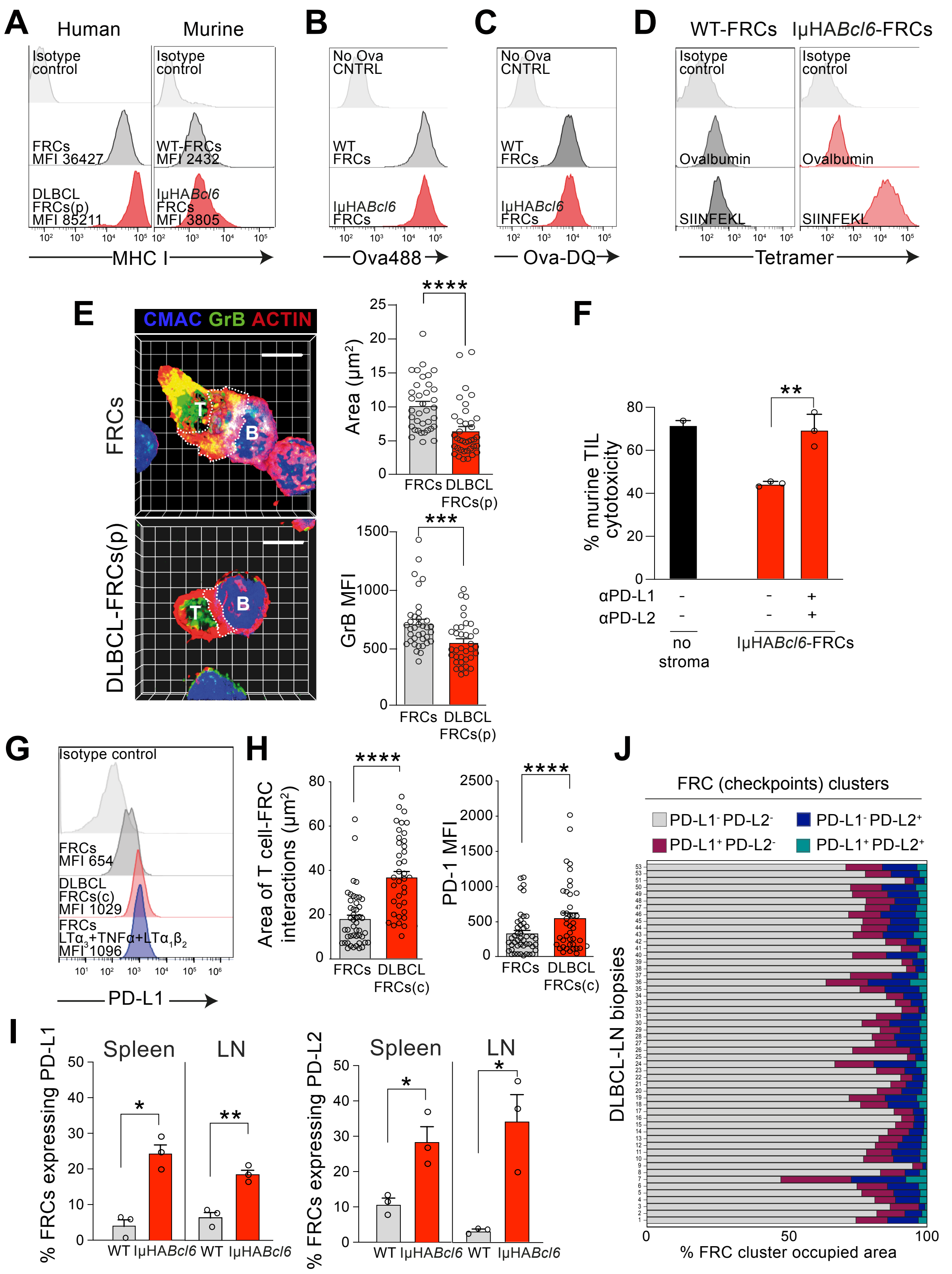

Supplemental Figure 7. DLBCL-activated FRCs dampen CD8$^+$ TIL killing function via the aberrant expression of co-inhibitory ligands. (**A**) Flow histogram analysis of MHC I expression in human (left panel) and murine (right panel) FRCs or WT-FRCs and DLBCL-FRCs(p) and IμHA*Bcl6*-FRCs. (**B**, **C**, **D**) Flow cytometric analysis of the ability of WT-FRCs and IμHA*Bcl6*-FRCs to internalize fluorescent Ovalbumin peptides (Ova-488) (**B**), to process fluorescent Ovalbumin peptides (Ova-DQ) (**C**) and to internalize, process and present Ovalbumin antigens on MHC I (**D**). The SIINFEKL Ova peptide was used as positive

control. (**E**) Left panels: representative confocal images of immune synapse formation between CD8$^+$ TILs and DLBCL B cells following exposure of TILs to either FRCs or DLBCL-FRCs(p) (n=1 patient). TILs, DLBCL B cells and DLBCL-FRCs(p) were autologous and isolated from patient DLBCL-LN tissue. Scale bar, 10μm. Right panels: upper graph: quantitation of the immune synapse area formed between CD8$^+$ TILs and tumor B cells. Lower graph: quantitation of Granzyme B (GrB) mean fluorescence intensity (MFI) at TIL synapses. (**F**) Measurement of CD8$^+$ TIL (IμHA*Bcl6*) cytolytic activity against DLBCL B cells in absence of FRCs or in presence of IμHA*Bcl6*-FRCs pre-treated with control isotype or blocking antibodies against PD-L1 and PD-L2. (**G**) Flow histograms showing PD-L1 expression on FRCs, DLBCL-FRCs(c) (SU-DHL16) or FRCs treated with recombinant TNFα, LTα_3 and LT$\alpha_1\beta_2$. (**H**) Quantitation of CD8$^+$ T cell:FRC contact areas between CD8$^+$ T cells and FRCs or DLBCL-FRCs(c) (SU-DHL16). Left graph: contact site area. Right graph: PD-1 MFI at T cell-FRC interaction site. (**I**) Quantitation of the area occupied by PDPN$^+$ FRCs expressing PD-L1 (left) or PD-L2 (right) in spleens and LNs from WT (n=3) and IμHA*Bcl6* (n=3) mice. (**J**) Frequency of FRCs expressing PD-L1 and/or PD-L2 within the FRC network of 53 DLBCL-LNs (analyzed IMC data). Data in (**E**, **F**, **H**, **I**) are shown as mean ± SEM, $****P < .0001$, $***P<.001$, $**P < .01$, $*P < .05$, one-way Anova with Tukey's multiple comparisons test (**F**) or Mann–Whitney U-test (**E**, **H**, **I**).

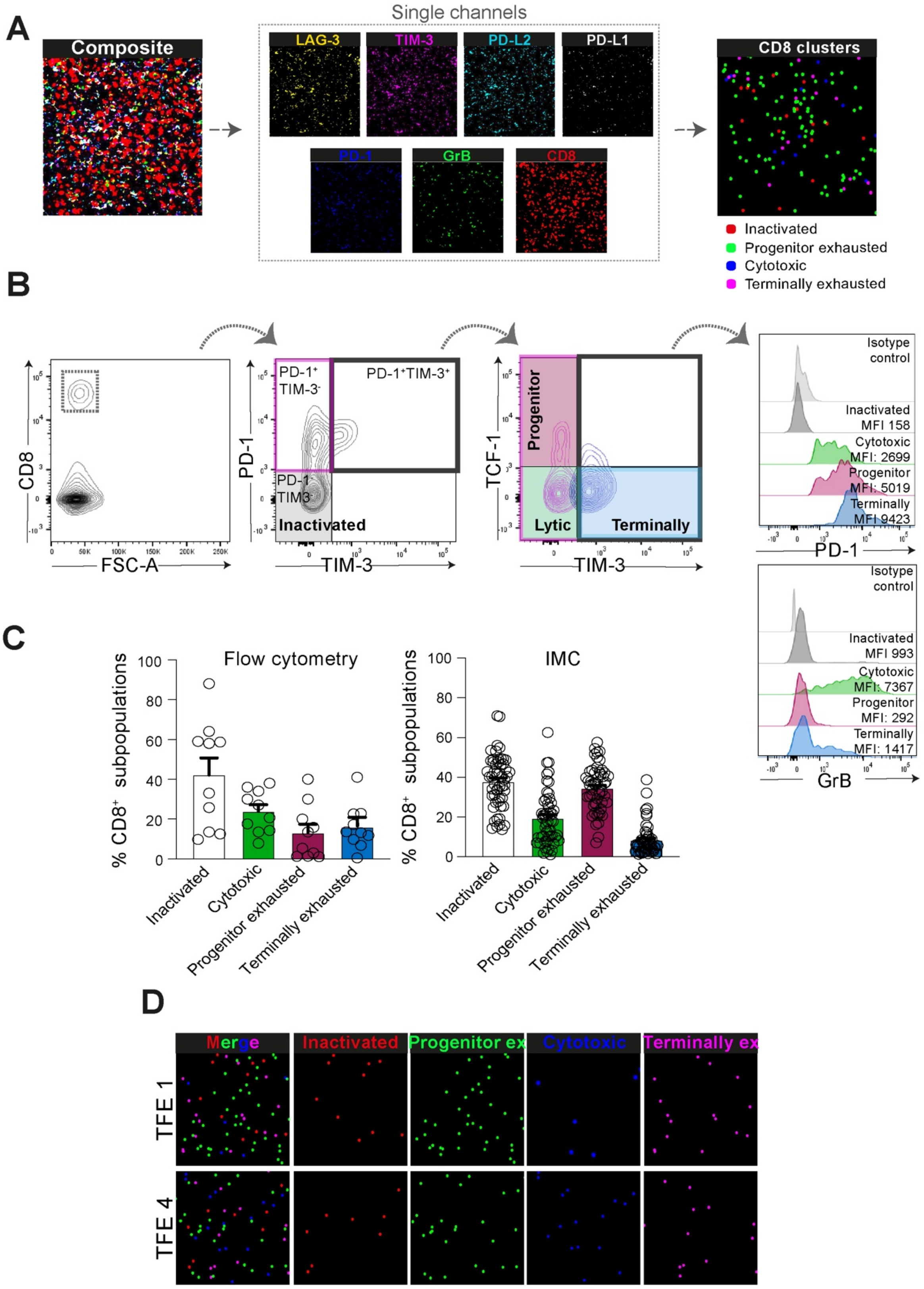

Supplemental Figure 8. IMC analysis of the CD8$^+$ TIL:FRC network defines distinct DLBCL microenvironments. (**a**) CD8$^+$ TIL phenotypic clusters identification in DLBCL-LNs. Left: composite IMC image of the 7 markers used to cluster single CD8$^+$ T cells in the 53 DLBCL-LN patient samples. Middle: single channel IMC images for the analyzed markers: LAG-3, TIM-3, PD-L2, PD-L1, PD-1, GrB and CD8. Right: schematic image showing the center of the cell for each identified CD8$^+$ T cell. The different colors

correspond to the identified $CD8^+$ phenotypic identities: inactivated (red), progenitor exhausted (green), cytotoxic (blue), terminally exhausted (pink). (**B**) Flow cytometry gating strategy used to identify the different $CD8^+$ T cell phenotypic subpopulations present in DLBCL-LN biopsies based on their expression of immune checkpoints (PD-1, TIM-3), the transcription factor TCF-1 and cytolytic molecule (GrB). Histograms show the MFI of PD-1 (top panel) and GrB (bottom panel) across the identified CD8 subpopulations (inactivated: dark grey, progenitor exhausted: pink, cytotoxic: green and terminally exhausted: blue). This analysis confirmed the presence of a non-activated $PD\text{-}1^-$ $TIM\text{-}3^-$, GrB^{low} $CD8^+$ T cell population. Analysis of $PD\text{-}1^+$ TILs identified a $TCF\text{-}1^+$ population lacking GrB and TIM-3 expression, consistent with a progenitor exhausted population. In contrast, $PD\text{-}1^+$ $TCF\text{-}1^-$ subsets were distinguished by their expression of TIM-3 that identified terminally exhausted ($PD\text{-}1^+$ $TIM\text{-}3^+$ $TCF\text{-}1^-$ GrB^{int}) and cytotoxic ($PD\text{-}1^+$ $TIM\text{-}3^-$ $TCF\text{-}1^-$ GrB^{high}) $CD8^+$ subsets. (**C**) Frequency of the different $CD8^+$ phenotypic identities present in DLBCL-LN biopsies identified by flow cytometry (left bar chart) and IMC (right panel) (each dot represents a DLBCL patient sample). (**D**) Representative IMC images of a patient belonging to TFE1 (upper panels) and TFE4 (lower panels) showing the distribution of the different $CD8^+$ TIL phenotypic identities: inactivated (red), progenitor exhausted (green), cytotoxic (blue), terminally exhausted (pink).

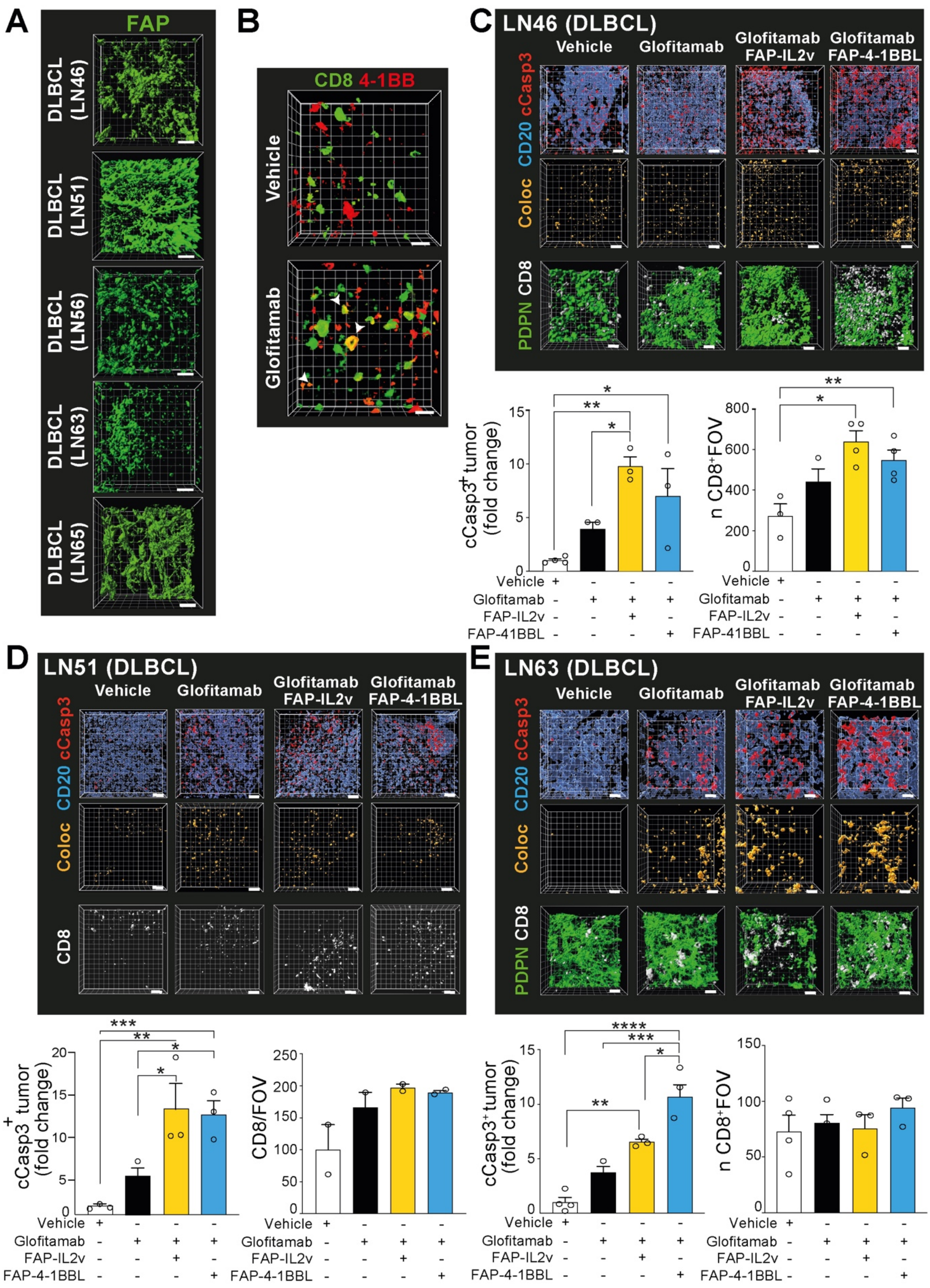
A
FAP
DLBCL (LN46)
DLBCL (LN51)
DLBCL (LN56)
DLBCL (LN63)
DLBCL (LN65)
B
CD8 4-1BB
Vehicle
Glofitamab
C
LN46 (DLBCL)
Vehicle
Glofitamab
Glofitamab FAP-IL2v
Glofitamab FAP-4-1BBL
CD20 cCasp3
Coloc
PDPN CD8
cCasp3+ tumor (fold change)
n CD8+FOV
Vehicle
Glofitamab
FAP-IL2v
FAP-41BBL
D
LN51 (DLBCL)
CD8
CD8/FOV
FAP-4-1BBL
E
LN63 (DLBCL)

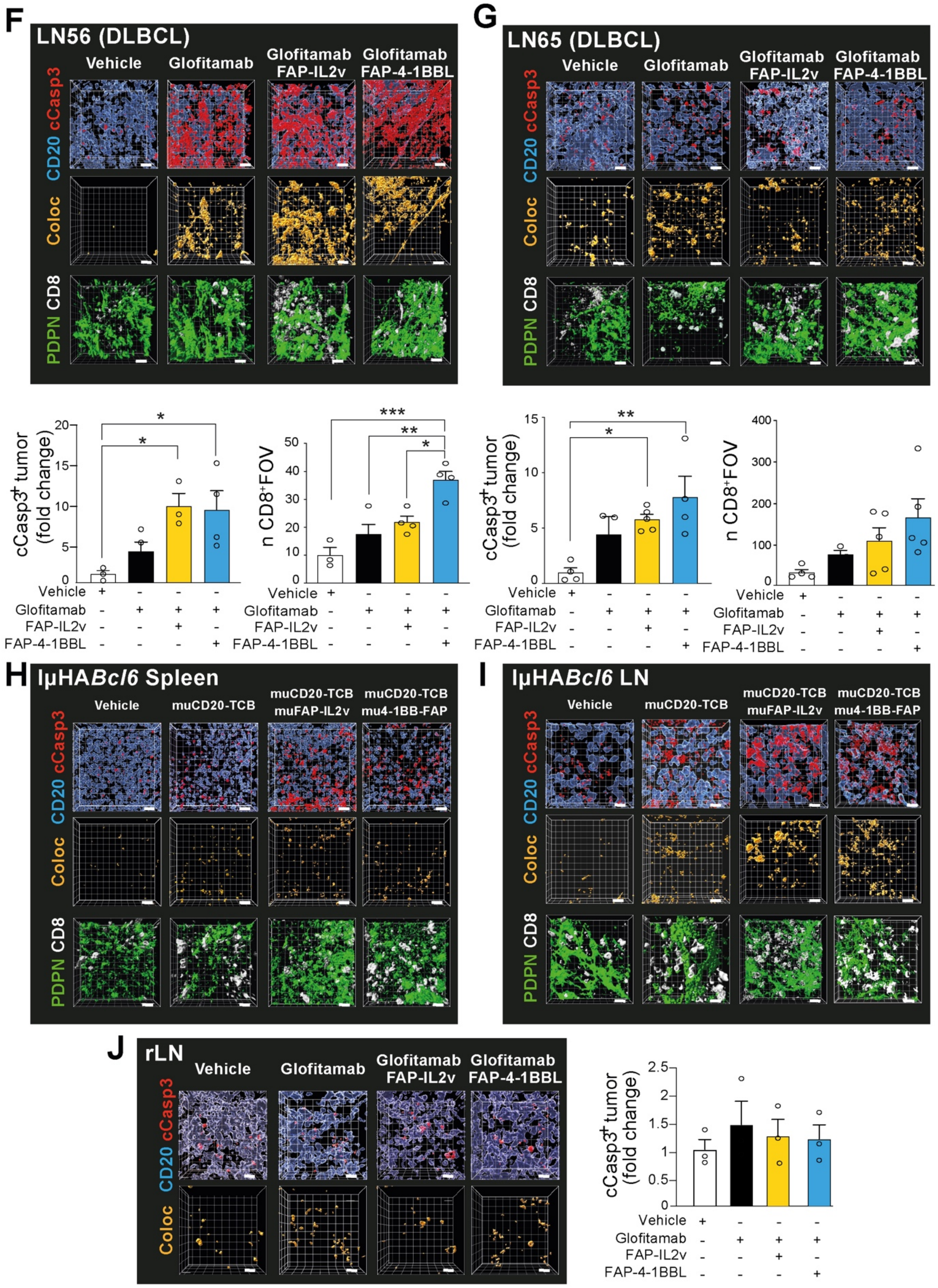

Supplemental Figure 9. Glofitamab-induced anti-tumor T cell activity is enhanced with the addition of FRC-targeted immunotherapy in human and murine DLBCL. (**a**) 3D image reconstruction of DLBCL organotypic specimens (n=5) stained after collection and analyzed using confocal microscopy. (**B**) 4-1BB expression on $CD8^{+}$ TILs at baseline and after 48h treatment with glofitamab. (**C**-**G** and **K**) Confocal 3D reconstructions of 5 patient DLBCL (**C**-**G**) or rLN (**J**) organotypic cultures treated for 48 hours with vehicle

control antibodies (DP47-TCB, DP47-4-1BBL, FAP-PGLALA) or with glofitamab alone or in combination with FAP-IL2v or FAP-4-1BBL. Bar charts show quantification of c-Casp3^{+} tumor cells (fold change compared to vehicle treatment) and the number of CD8^{+} TILs/FOV for each treatment group. (**H**, **I**) Confocal 3D image reconstructions of representative IμHA*Bcl6* spleen (**H**) and LN (**I**) organotypic cultures treated for 48 hours with vehicle or surrogate murine (mu) muCD20-TCB alone or in combination with muFAP-IL2v or mu4-1BB-FAP immunotherapy drugs (n=3). Data in (**C**-**G**, **J**) are shown as mean ± SEM, ****P < .0001, ***P<.001, **P < .01, *P < .05, one-way Anova with Tukey's multiple comparisons test.

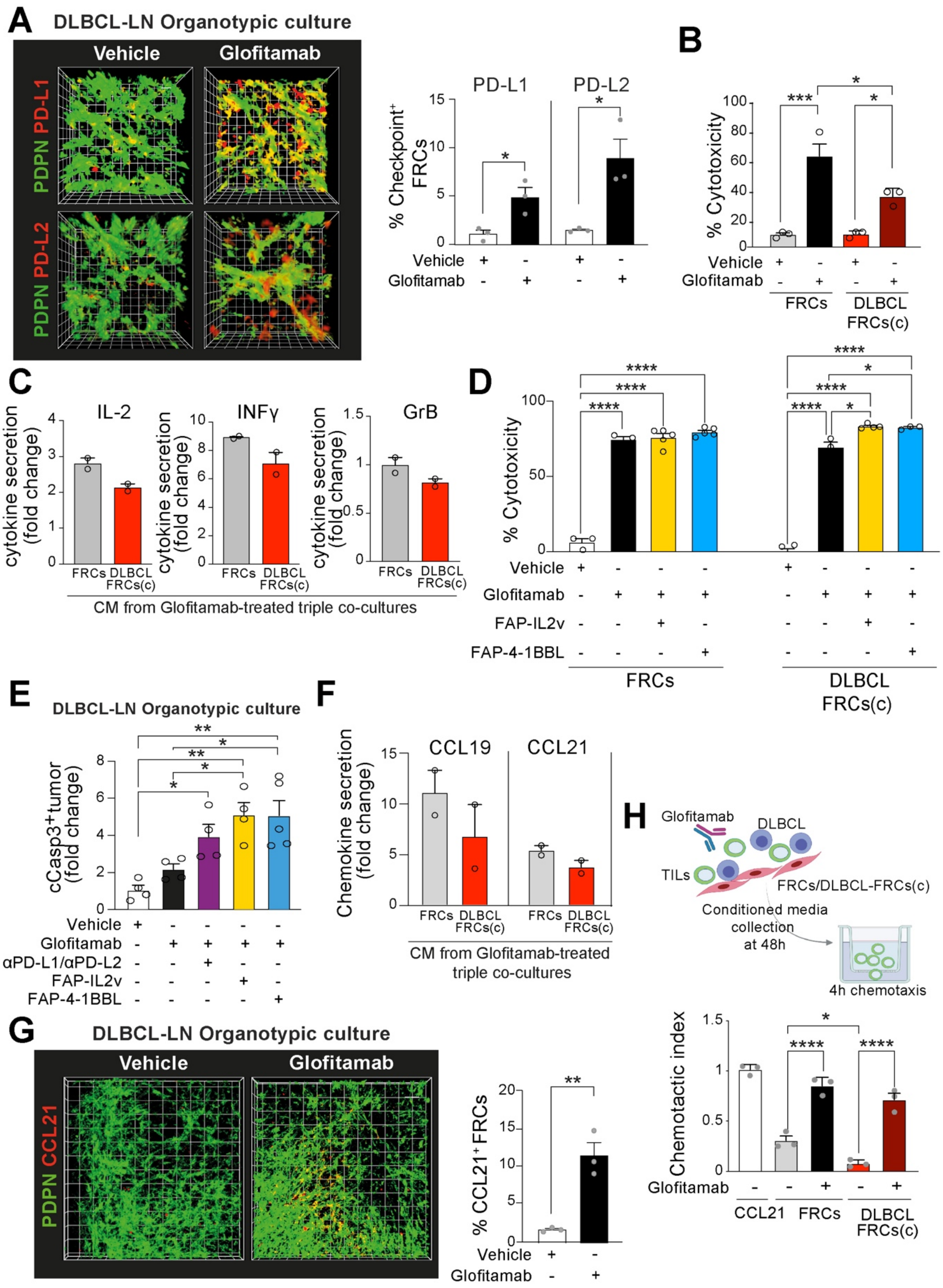
A
DLBCL-LN Organotypic culture
Vehicle
Glofitamab
PDPN PD-L1
PDPN PD-L2
B
PD-L1
PD-L2
% Checkpoint+ FRCs
Vehicle
Glofitamab
% Cytotoxicity
FRCs
DLBCL FRCs(c)
C
IL-2
INFγ
GrB
cytokine secretion (fold change)
CM from Glofitamab-treated triple co-cultures
D
% Cytotoxicity
FAP-IL2v
FAP-4-1BBL
E
DLBCL-LN Organotypic culture
cCasp3+tumor (fold change)
αPD-L1/αPD-L2
F
CCL19
CCL21
Chemokine secretion (fold change)
CM from Glofitamab-treated triple co-cultures
H
Glofitamab
DLBCL
TILs
FRCs/DLBCL-FRCs(c)
Conditioned media collection at 48h
4h chemotaxis
Chemotactic index
CCL21
G
DLBCL-LN Organotypic culture
PDPN CCL21
% CCL21+FRCs

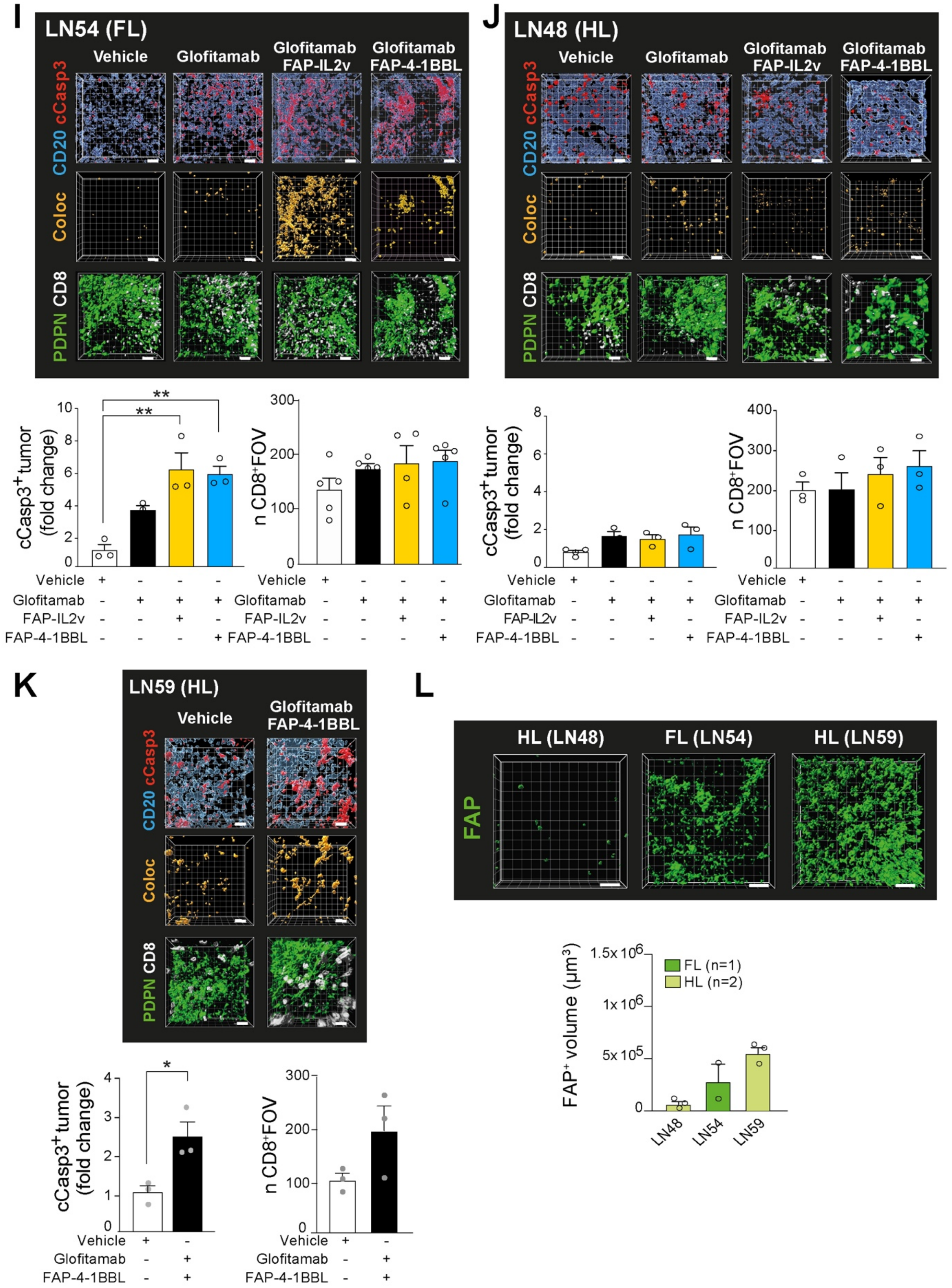

Supplemental Figure 10. DLBCL-FRCs suppress T cell bispecific antibody-induced TIL killing function that can be overcome using FRC-targeted immunostimulatory drugs. (**A**) Left panels: 3D confocal image reconstruction of an organotypic DLBCL culture treated for 48 hours with control antibody (vehicle, DP47-TCB) or glofitamab (CD20-TCB) and stained for PDPN and PD-L1 or PD-L2. Bar charts show

quantification of PDPN^{+} FRCs expressing PD-L1 and PD-L2. (**B**) Human CD8^{+} TIL cytotoxicity against DLBCL cells in triple culture assays. TILs and autologous DLBCL cells were treated with control antibody (DP47-TCB) or glofitamab for 48 hours in the presence of FRCs or DLBCL-FRCs(c) (primary DLBCL cells). Primary DLBCL cells and TILs were autologous (representative patient data shown from n=3 primary DLBCL patient samples). (**C**) Effector cytokines (IL-2, INFγ, GrB) quantification (fold change increase compared to untargeted control DP47-TCB antibody treatment) in the conditioned media (CM) of glofitamab-treated (48 hours) human triple co-cultures set-up and described in (**B**). (**D**) Human CD8^{+} TIL cytotoxicity against DLBCL cells in triple culture assays. TILs and autologous DLBCL cells were treated with the drugs indicated for 48 hours in the presence of FRCs or DLBCL-FRCs(c) (primary DLBCL cells). Primary DLBCL cells and TILs were autologous (representative patient data shown from n=3 primary DLBCL patient samples). (**E**) Quantification of c-Casp3^{+} DLBCL B cells (fold change compared to vehicle treatment) from an organotypic culture treated for 48 hours with control antibodies (Vehicle: DP47-TCB, FAP-PGLALA, isotype control) or with glofitamab alone or in combination with anti-PD-L1 + anti-PD-L1 or FAP-IL2v or FAP-4-1BBL. (**F**) T cell chemokines (CCL19, CCL21) quantification (fold change increase compared to untargeted control DP47-TCB antibody treatment) in the CM of glofitamab-treated (48 hours) human triple co-cultures set-up and described in (**B**). (**G**) Representative 3D confocal image reconstruction of a DLBCL organotypic culture treated for 48 hours with control antibody (DP47-TCB) or glofitamab. Bar chart shows quantification of PDPN^{+} FRCs expressing CCL21. (**H**) Human CD3^{+} TIL chemotaxis towards the CM of triple culture assays. CM was harvested from cultures where TILs and autologous DLBCL cells were treated with DP47-TCB (control) or glofitamab for 48 hours in the presence of FRCs or DLBCL-FRCs(c) (primary DLBCL cells). Separate untreated TILs were added to the upper chambers of transwell inserts for chemotaxis assays. Primary DLBCL cells and TILs were autologous (representative patient data shown from n=3 primary DLBCL patient samples). (**I**-**K**) Confocal image 3D reconstructions of one relapsed follicular lymphoma (**I**), and 2 Hodgkin lymphoma (**J**, **K**) organotypic cultures treated for 48 hours with vehicle control antibodies (DP47-TCB, FAP-PGLALA) or with glofitamab alone or in combination with FAP-IL2v or FAP-4-1BBL as indicated. Bar charts show quantification of c-Casp3^{+} B cells (fold change compared to vehicle treatment) and the number of CD8^{+} TILs/FOV for each treatment group. (**L**) Confocal analysis of FAP expression in the lymphoma (**I**-**K**) organotypic tissues showing representative 3D image reconstruction and quantification of the volume occupied by FAP^{+} FRCs. Data in (**A**-**L**) are shown as mean ± SEM, *****P* < .0001, ****P*<.001, ***P* < .01, **P* < .05, one-way Anova with Tukey's multiple comparisons test (**B**, **D**, **E**, **H**, **I**-**K**) or Mann–Whitney U-test (**A**, **C**, **F**, **G**).

List of Movies

Movie S1. Time-lapse video microscopy showing the motility of human $CD3^+$ TILs on a monolayer of unconditioned FRCs. Time of imaging 1h.

Movie S2. Time-lapse video microscopy showing the motility of human $CD3^+$ TILs on a monolayer of DLBCL-FRCs(c) (conditioning with primary DLBCL B cells). Time of imaging 1h.

Movie S3. Time-lapse video microscopy showing the motility of murine $CD3^+$ TILs isolated from the spleen of IµHA*Bcl6* mice on a monolayer of WT-FRCs. Time of imaging 1h.

Movie S4. Time-lapse video microscopy showing the motility of murine $CD3^+$ TILs isolated from the spleen of IµHA*Bcl6* on a monolayer of IµHA*Bcl6*-FRCs. Time of imaging 1h.

Movie S5. 3D time-lapse video microscopy showing the motility of human $CD3^+$ TILs on a 3D network of FRCs. Time of imaging 3h.

Movie S6. 3D time-lapse video microscopy showing the motility of human $CD3^+$ TILs on a 3D network of DLBCL-FRCs(c) (conditioning with primary DLBCL B cells). Time of imaging 3h.

REFERENCES

1. Abdulla M, Laszlo S, Triumf J, Hedstrom G, Berglund M, Enblad G, et al. A population-based study of cellular markers in R-CHOP treated diffuse large B-cell lymphoma patients. *Acta Oncol.* 2016;55(9-10):1126-31.
2. Enblad G, Karlsson H, Gammelgard G, Wenthe J, Lovgren T, Amini RM, et al. A Phase I/IIa Trial Using CD19-Targeted Third-Generation CAR T Cells for Lymphoma and Leukemia. *Clin Cancer Res.* 2018;24(24):6185-94.
3. Cattoretti G, Pasqualucci L, Ballon G, Tam W, Nandula SV, Shen Q, et al. Deregulated BCL6 expression recapitulates the pathogenesis of human diffuse large B cell lymphomas in mice. *Cancer Cell.* 2005;7(5):445-55.
4. Bacac M, Colombetti S, Herter S, Sam J, Perro M, Chen S, et al. CD20-TCB with Obinutuzumab Pretreatment as Next-Generation Treatment of Hematologic Malignancies. *Clin Cancer Res.* 2018;24(19):4785-97.
5. Claus C, Ferrara C, Xu W, Sam J, Lang S, Uhlenbrock F, et al. Tumor-targeted 4-1BB agonists for combination with T cell bispecific antibodies as off-the-shelf therapy. *Sci Transl Med.* 2019;11(496).
6. Waldhauer I, Gonzalez-Nicolini V, Freimoser-Grundschober A, Nayak TK, Fahrni L, Hosse RJ, et al. Simlukafusp alfa (FAP-IL2v) immunocytokine is a versatile combination partner for cancer immunotherapy. *MAbs.* 2021;13(1):1913791.
7. Poirot L, Philip B, Schiffer-Mannioui C, Le Clerre D, Chion-Sotinel I, Derniame S, et al. Multiplex Genome-Edited T-cell Manufacturing Platform for "Off-the-Shelf" Adoptive T-cell Immunotherapies. *Cancer Res.* 2015;75(18):3853-64.
8. Graham CE, Jozwik A, Quartey-Papafio R, Ioannou N, Metelo AM, Scala C, et al. Gene-edited healthy donor CAR T cells show superior anti-tumour activity compared to CAR T cells derived from patients with lymphoma in an in vivo model of high-grade lymphoma. *Leukemia.* 2021.
9. Fletcher AL, Malhotra D, Acton SE, Lukacs-Kornek V, Bellemare-Pelletier A, Curry M, et al. Reproducible isolation of lymph node stromal cells reveals site-dependent differences in fibroblastic reticular cells. *Front Immunol.* 2011;2:35.
10. Salmon H, Rivas-Caicedo A, Asperti-Boursin F, Lebugle C, Bourdoncle P, and Donnadieu E. Ex vivo imaging of T cells in murine lymph node slices with widefield and confocal microscopes. *J Vis Exp.* 2011(53):e3054.
11. Tomei AA, Siegert S, Britschgi MR, Luther SA, and Swartz MA. Fluid flow regulates stromal cell organization and CCL21 expression in a tissue-engineered lymph node microenvironment. *J Immunol.* 2009;183(7):4273-83.
12. Ramsay AG, Clear AJ, Fatah R, and Gribben JG. Multiple inhibitory ligands induce impaired T-cell immunologic synapse function in chronic lymphocytic leukemia that can be blocked with lenalidomide: establishing a reversible immune evasion mechanism in human cancer. *Blood.* 2012;120(7):1412-21.
13. Acton SE, Farrugia AJ, Astarita JL, Mourão-Sá D, Jenkins RP, Nye E, et al. Dendritic cells control fibroblastic reticular network tension and lymph node expansion. *Nature.* 2014;514(7523):498-502.
14. Riedel A, Shorthouse D, Haas L, Hall BA, and Shields J. Tumor-induced stromal reprogramming drives lymph node transformation. *Nat Immunol.* 2016;17(9):1118-27.
15. Berg S, Kutra D, Kroeger T, Straehle CN, Kausler BX, Haubold C, et al. ilastik: interactive machine learning for (bio)image analysis. *Nat Methods.* 2019;16(12):1226-32.
16. Chen H, Lau MC, Wong MT, Newell EW, Poidinger M, and Chen J. Cytofkit: A Bioconductor Package for an Integrated Mass Cytometry Data Analysis Pipeline. *PLoS Comput Biol.* 2016;12(9):e1005112.
17. Dobin A, Davis CA, Schlesinger F, Drenkow J, Zaleski C, Jha S, et al. STAR: ultrafast universal RNA-seq aligner. *Bioinformatics.* 2013;29(1):15-21.
18. Subramanian A, Tamayo P, Mootha VK, Mukherjee S, Ebert BL, Gillette MA, et al. Gene set enrichment analysis: a knowledge-based approach for interpreting genome-wide expression profiles. *Proc Natl Acad Sci U S A.* 2005;102(43):15545-50.

19. Stuart T, Butler A, Hoffman P, Hafemeister C, Papalexi E, Mauck WM, 3rd, et al. Comprehensive Integration of Single-Cell Data. *Cell.* 2019;177(7):1888-902 e21.
20. Rodda LB, Lu E, Bennett ML, Sokol CL, Wang X, Luther SA, et al. Single-Cell RNA Sequencing of Lymph Node Stromal Cells Reveals Niche-Associated Heterogeneity. *Immunity.* 2018;48(5):1014-28 e6.
21. Kapoor VN, Muller S, Keerthivasan S, Brown M, Chalouni C, Storm EE, et al. Gremlin 1(+) fibroblastic niche maintains dendritic cell homeostasis in lymphoid tissues. *Nat Immunol.* 2021;22(5):571-85.
